# WNT/β-catenin signaling regulates mitochondrial activity to alter the oncogenic potential of melanoma in a PTEN-dependent manner

**DOI:** 10.1038/onc.2016.450

**Published:** 2017-01-16

**Authors:** K Brown, P Yang, D Salvador, R Kulikauskas, H Ruohola-Baker, A M Robitaille, A J Chien, R T Moon, V Sherwood

**Affiliations:** 1 School of Pharmacy, University of East Anglia, Norwich Research Park, Norwich, UK; 2 Department of Pharmacology, Howard Hughes Medical Institute, Institute for Stem Cell and Regenerative Medicine, Seattle, WA, USA; 3 Division of Cancer Research, Jacqui Wood Cancer Centre, Ninewells Hospital and Medical School, University of Dundee, Dundee, UK; 4 Department of Biochemistry, University of Washington, Seattle, WA, USA; 5 Division of Dermatology, University of Washington, Seattle, WA, USA

## Abstract

Aberrant regulation of WNT/β-catenin signaling has a crucial role in the onset and progression of cancers, where the effects are not always predictable depending on tumor context. In melanoma, for example, models of the disease predict differing effects of the WNT/β-catenin pathway on metastatic progression. Understanding the processes that underpin the highly context-dependent nature of WNT/β-catenin signaling in tumors is essential to achieve maximal therapeutic benefit from WNT inhibitory compounds. In this study, we have found that expression of the tumor suppressor, phosphatase and tensin homolog deleted on chromosome 10 (PTEN), alters the invasive potential of melanoma cells in response to WNT/β-catenin signaling, correlating with differing metabolic profiles. This alters the bioenergetic potential and mitochondrial activity of melanoma cells, triggered through regulation of pro-survival autophagy. Thus, WNT/β-catenin signaling is a regulator of catabolic processes in cancer cells, which varies depending on the metabolic requirements of tumors.

## Introduction

The importance of WNT signaling in metazoans cannot be underestimated; the pathways are essential in embryonic development, coordinating correct tissue patterning and for homeostasis in adult tissues. As such, aberrant WNT signaling can lead to a host of embryonic malformations, degenerative diseases and cancer. Thus, understanding how WNT signaling affects cells and tissues has far reaching implications in animal biology. WNT proteins comprise a large family of secreted glycoproteins (19 members in vertebrates) that modulate a number of signal transduction pathways in a highly tissue context-dependent manner.^1^ The pathways can be subdivided into two categories based on their capacity to transduce signals via the pleiotropic protein, β-catenin (encoded by the *CTNNB1* gene), and are therefore referred to as either eliciting β-catenin-dependent or -independent signaling (sometimes also referred to as canonical or non-canonical WNT signaling, respectively). However, the pathways do not function in an autonomous manner, but rather exist in a signaling network where there is concomitant cross-talk and regulation between the β-catenin-dependent, and -independent pathways.^2^ The WNT/β-catenin pathway results in the stabilization of a cytoplasmic pool of β-catenin that would otherwise be marked for proteasomal-mediated degradation by a destruction complex, composed of (among other proteins) APC (encoded by *APC*), AXIN-1/2 (encoded by *AXIN1/2*) and GSK-3β (encoded by *GSK3B*). Upon binding to receptors of the Frizzled (FZD) family and the co-receptors, LRP5/6, extracellular WNT stimulation transmits a signal to the intracellular Disheveled (DVL) adaptor, which inhibits the destruction complex and allows nuclear translocation of stabilized β-catenin to activate gene transcription. In the nucleus, β-catenin acts as a cofactor with transcription factor/lymphoid enhancer-binding element transcription factors to stimulate expression of WNT target genes such as *AXIN2* and *MYC.*^3, 4, 5^

Since the 1980s researchers have known that the WNT pathways have an influential role in carcinogenesis, after WNT1 was shown to induce the formation of mammary tumors in mice.^6, 7, 8^ The WNT signaling network is now known to regulate oncogenesis in a diverse array of cancers including (in addition to breast), tumors derived from the blood, gastro-intestinal tract, brain, prostate, ovaries, liver and skin, to name a few. Traditionally, the WNT/β-catenin pathway has been viewed as oncogenic, where hyperactivity of the pathway promotes carcinogenesis. For example, in colorectal cancer loss-of-function mutations in *APC* can be detected in up to 80% of tumors,^9, 10^ leading to increased β-catenin signaling and prompting researchers to develop inhibitory compounds for the pathway.^11^ However, it is not that simple in all tumor contexts, as high levels of nuclear β-catenin does not always correlate with poor prognosis for all tumor types, including medulloblastoma,^12^ ovarian cancer,^13^ prostate cancer^14^ and melanoma.^15^

Melanoma is a malignancy derived from the pigment-producing cells, melanocytes, and alarmingly has some of the fastest growing incidence rates among human cancers worldwide.^16, 17^ WNT/β-catenin signaling in melanoma tumors has previously been shown to correlate with improved survival in patients, which was corroborated by murine xenograft models where melanoma cells overexpressing WNT3A exhibit reduced tumor volumes and metastasis compared with parental cells.^15^ Furthermore, reduced β-catenin expression has been associated with progression of melanoma in additional clinical cohorts.^18, 19, 20, 21^ However, other experimental work challenges the assumption that increased WNT/β-catenin signaling reduces the growth and spread of melanocytic tumors. Engineered murine models of melanoma that express melanocyte-specific phosphatase and tensin homolog deleted on chromosome 10 (PTEN) loss and the constitutively activating BRAF^V600E^ mutation (two mutations commonly associated with melanocytic tumors in patients), exhibit highly metastatic and aggressive tumors when β-catenin is stabilized.^22^ These observations suggest that subsets of melanoma tumors containing distinct mutational contexts, respond to stabilized β-catenin with potentially differing effects on disease progression, highlighting the need to better understand the role of the WNT/β-catenin pathway in melanoma cell behavior.

Interest in the topic of cancer metabolism has been revived in recent years as accumulating evidence has demonstrated the contribution that these metabolic alterations have on the establishment and progression of tumors.^23^ Indeed, metabolic reprogramming is a hallmark of cancer,^24^ which has been demonstrated in a number of tumor types to be regulated by WNT signaling (as we have recently reviewed, Sherwood^25^), including in melanoma.^26^ We have previously shown that WNT5A (signaling in a β-catenin-independent manner) promotes aerobic glycolysis in melanoma cells,^26^ which may contribute to the pro-metastatic effects of this signaling pathway in melanoma. Furthermore, the WNT/β-catenin pathway has also been shown to promote aerobic glycolysis in colorectal cancer and promote cell proliferation.^27^ Currently it is unknown if WNT/β-catenin signaling can also reprogram melanoma cell metabolism and if so, what effect this may have on tumorigenic state.

Here we compare melanomas expressing wild-type PTEN (PTEN^WT^) with those expressing genetic deletions in this tumor suppressor and analyze the phenotypic effects of the WNT/β-catenin pathway in both subsets. We demonstrate that the WNT/β-catenin pathway has profound effects on melanoma cell invasion, metastasis and metabolic status between tumor cells depending on PTEN expression status. Moreover we provide evidence that in melanoma cells expressing PTEN, alterations in cellular metabolism are associated with the control of mitochondrial activity and remodeling. Taken together, these results support a model whereby WNT/β-catenin signaling controls subsets of melanoma cells based on PTEN expression status, which regulates metabolic behavior in the cells to alter their invasive potential.

## Results

### WNT3A increases apoptosis in melanoma cells

**Figure 1:**
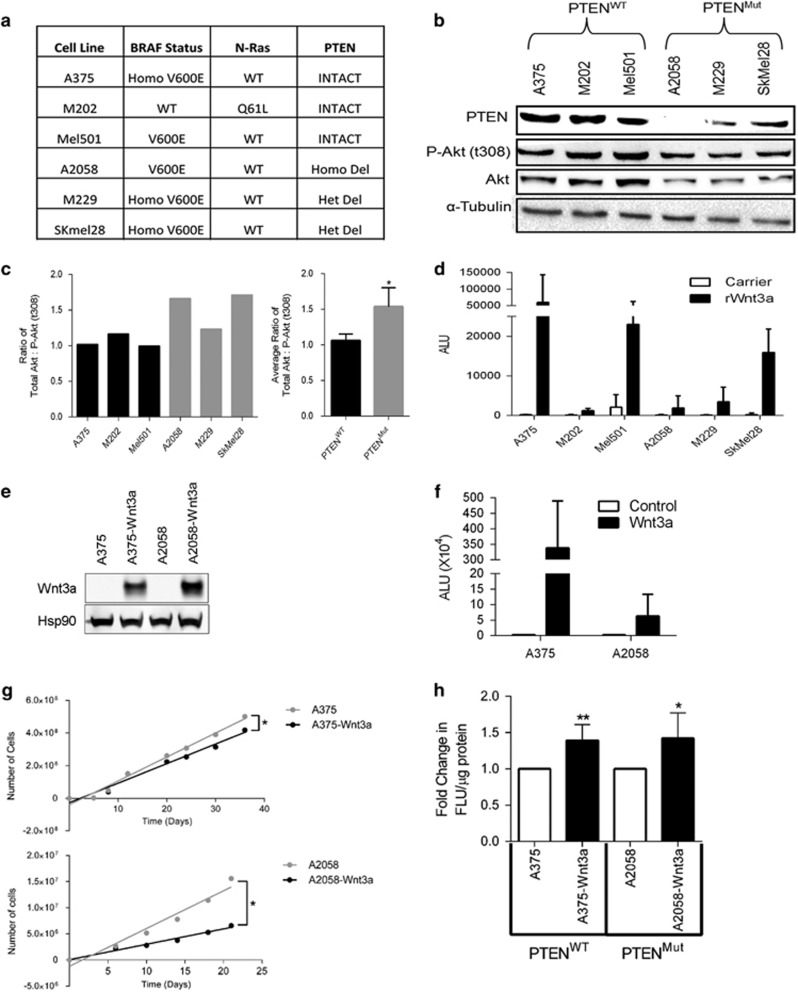
Melanoma cells exhibit increased apoptosis in response to WNT/β-catenin signaling. (**a**) Panel of chosen melanoma cells describing their associated genetic backgrounds for *BRAF*, *NRAS* and *PTEN* status. (**b**) Hetero- and homozygous mutations in the *PTEN* gene, result in depleted PTEN and phosphorylated-AKT (P-Akt) expression. In all, 10 μg of whole-cell lysate of PTEN^WT^ and PTEN^Mut^ lines were blotted for PTEN, AKT and P-AKT (t308) expression. α-Tubulin served as the loading control. (**c**) Ratio of the protein expression of AKT relative to P-AKT in individual melanoma cells and the average ratio of PTEN^WT^ and PTEN^Mut^ cell lines. Calculated from data presented in **b**. (**d**) WNT3A induces β-catenin-transcription factor/lymphoid enhancer-binding element reporter activity in melanoma cells. Results were normalized to the inverted promoter reporter plasmid (FOPFlash) after 24 h stimulation with carrier or rWNT3A (50 ng/ml). (**e**) Western blot analysis of WNT3A expression levels in A375 and A2058 control and WNT3A overexpression cells. Heat shock protein 90 (Hsp90) served as a loading control. (**f**) Stable overexpression of WNT3A in melanoma cells induces WNT/β-catenin signaling as measured using the TOPFlash β-catenin-transcription factor/lymphoid enhancer-binding element reporter. Results normalized to FOPFlash after 24 h of serum starvation. (**g**) Stable WNT3A overexpression reduces melanoma cell numbers. Live population cell counts of A375 and A2058 control, and WNT3A overexpression cells were carried out over 36 and 21 days, respectively. A paired Student's *t*-test was carried out on the total cell number, as indicated (*). (**h**) WNT3A increases apoptosis in melanoma cells. Fold-change in Apo-TRACE staining following 48 h serum starvation in A375 and A2058 control, and WNT3A overexpression cells. Fluorescence was normalized to total protein concentration. For all panels, mean±s.d. *P<0.05 or **P<0.01.

Driver mutations that result in hyper-activation of the mitogen-activated protein kinase pathway exist in the majority of melanoma tumors and occur predominantly through activating mutations of either BRAF (approximately 50%) or NRAS (approximately 20%),^28, 29, 30^ which tend to be mutually exclusive.^31^ Although less prevalent than mitogen-activated protein kinase pathway alterations, loss of PTEN (the lipid and protein phosphatase that negatively regulates the phosphatidylinositol-3-kinase (PI3K)-AKT pathway) through deletion, mutation or reduced expression, occurs in approximately 5–40% of melanocytic tumors.^31, 32, 33^ We selected a panel of cell lines with known status of these common mutations in melanoma (Figure 1a) to investigate the context-dependent nature of WNT/β-catenin signaling in melanocytic tumors. Half of the lines had PTEN^WT^ expression, whereas the other 50% had homo- or heterozygous deletions in PTEN (PTEN^Mut^; Figure 1a). The PTEN^Mut^ cells were confirmed to have reduced expression of PTEN compared with the PTEN^WT^ lines (Figure 1b) and higher levels of phosphorylated-AKT (P-AKT) on Thr308, showing that PI3K signaling is activated in the PTEN^Mut^ lines (Figures 1b and c).

WNT3A has been previously shown to signal through β-catenin in human melanoma cells.^15^ To confirm this finding in our melanoma panel, we ensured that recombinant WNT3A (rWNT3A) could activate a β-catenin-responsive reporter in the cells (Figure 1d). Furthermore, constitutive overexpression of WNT3A using a WNT3A-iresGFP reporter construct in two of the lines (one PTEN^WT^ line, A375; and one PTEN^Mut^ line, A2058; Figure 1e) also led to enhanced signaling from this reporter construct (Figure 1f), demonstrating that WNT3A activates β-catenin signaling the selected melanoma panel.

It is well known that the WNT/β-catenin pathway is associated with deregulated cell proliferation and neoplasia through multiple mechanisms including cell cycle control, regulation of apoptosis and mitotic control, as previously reviewed.^34^ To investigate the role of the WNT/β-catenin pathway in melanoma cells, we first analyzed long-term cell proliferation in response to forced WNT3A signaling. By monitoring population doublings over several weeks, we found that hyperactive WNT3A signaling resulted in reduced cell counts (Figure 1g), which is comparable with previous findings.^15^ Analysis of cell division showed no significant difference in G_0_/G_1_, S and G_2_/M phases of the cell cycle in response to overexpressed WNT3A in the melanoma cells (Supplementary Figure 1a), demonstrating that the differences in cell numbers during long-term proliferation assays identified in Figure 1g were not due to cell cycle arrest. The cell cycle analysis did, however, show a marked shift in the SubG_1_ population in response to WNT3A (Supplementary Figure 1a), suggesting there may be an accumulation of apoptotic cells. To confirm this, we analyzed the amount of cells committed to apoptosis through annexin V staining and found that WNT3A-activated signaling significantly increased apoptosis in melanoma cells (Figure 1h). This observation was further supported by the finding that WNT3A overexpression also led to a significant increase in cytochrome c release from the mitochondria to the cytosol (Supplementary Figure 1b). Taken together, our data show that WNT/β-catenin signaling increases apoptosis in melanoma cells (irrespective of PTEN mutational status).

### WNT3A regulates melanoma cell invasion in a PTEN-dependent manner

**Figure 2:**
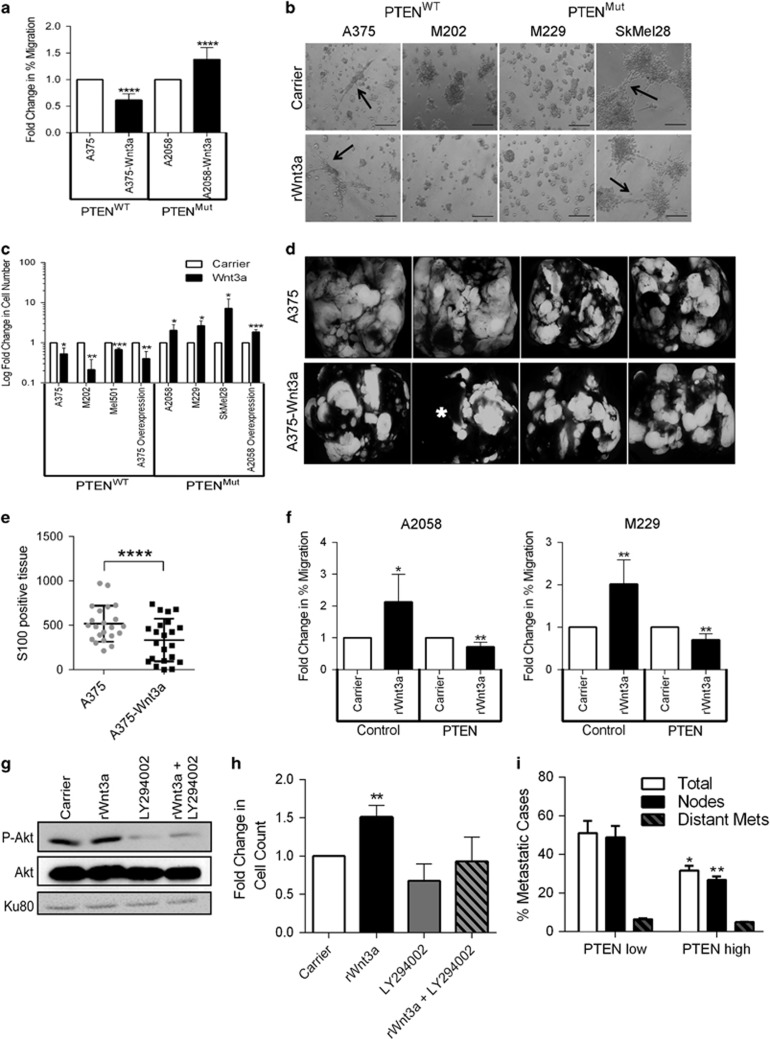
PTEN^WT^ melanoma cells exhibit reduced motility and metastasis in response to WNT/β-catenin signaling. (**a**) Fold-change in cell migration over 24 h in PTEN^WT^ and PTEN^Mut^ control and WNT3A overexpression cells. Cells were grown to confluency before the monolayer was wounded and scratch size measured before, and after 24 h. (**b**) Representative images of 3D cell growth following 48h stimulation with carrier or 50 ng/ml rWNT3A. Arrows highlight connected cell clusters, which are indicative of invasive phenotypes in melanoma cells. Scale bar, 200 μm. (**c**) Fold-change in cell invasion after 48 h compared with control cells. Parental lines were stimulated with carrier or 50 ng/ml rWNT3A. For panels **a** and **c**, mean shown±s.d. *P<0.05, **P<0.01, ***P<0.001 and ****P<0.0001. (**d**) × 0.75 magnification images of mouse lungs 4 weeks after tail vein injections of A375 control or WNT3A overexpression cells. Star highlights metastasis-free lung lobe. Example images shown. (**e**) Quantitative scoring of S100 staining on mouse lung sections. Mean shown±s.d. ****P<0.0001. **(f)** Fold-change in cell migration over 24 h in control and PTEN overexpressing A2058 and M229 cells, following 48 h stimulation with carrier or 50 ng/ml rWNT3A. Cells were seeded to confluency before the monolayer was wounded and scratch size measured before, and after 24 h. Mean shown±s.d. *P<0.05, **P<0.01. (**g**) 2 μM of LY294002 inhibits AKT activity in PTEN^Mut^ A2058 cell, after 48 h of stimulation. In total, 15 μg of whole-cell lysate was blotted for AKT and P-AKT, following 48 h stimulation with carrier or 50 ng/ml rWNT3A. Ku80 served as the loading control. (**h**) Fold-change in A2058 cell invasion after 48 h of treatment with 50 ng/ml rWNT3A/carrier with/without 2 μM LY294002, as indicated. LY294002 was kept on the cells throughout the experiment. **(i)** Percentage of metastatic tumors in cutaneous melanoma patients (TCGA data set) with high *AXIN2* expression (*n*=209). *P<0.05 or **P<0.01. Distant Mets, distant metastasis; Nodes, nodal metastasis.

In addition to the effects on tumor growth, WNT signaling pathways are also regulators of cell motility, able to enhance or inhibit the migratory capacity of cancer cells in a cell-type-specific manner.^35, 36, 37, 38, 39, 40, 41^ Using the two WNT3A overexpression lines (Figure 1e), we evaluated the cells' ability to fill a wound scratch and found an inverse correlation in cell migration in response to high WNT/β-catenin signaling between the two lines (Figure 2a and Supplementary Figure 2a). Specifically, cell migration was reduced by WNT3A signaling in the PTEN^WT^ A375 cells, whereas in contrast, this effect was enhanced in the PTEN^Mut^ A2058 cells (Figure 2a and Supplementary Figure 2a). This result suggested that WNT/β-catenin signaling may control melanoma cell motility in a PTEN expression-dependent manner.

Interestingly it has previously been reported that in melanoma, depending on the cell line used, β-catenin signaling can either enhance or reduce cell invasion,^18, 42^ therefore we investigated the effects of WNT3A on the invasive capacity of our panel of melanoma cells. WNT signaling has been linked to the metastatic spread of melanoma cells by driving phenotype switching, a process that forces the cells to switch between invasive and proliferative states, which can be assessed by three-dimensional (3D) culture of melanoma cells.^43^ Proliferative phenotypes form round, individual colonies and invasive phenotypes form interconnected colonies.^43^ We were unable to switch the 3D phenotypes of a subset of our melanoma panel (two PTEN^WT^ and two PTEN^Mut^ lines) in response to rWNT3A stimulation (Figure 2b), suggesting that activation of WNT/β-catenin signaling is insufficient to drive phenotype switching in 3D culture in the selected panel. We did, however, find that WNT3A signaling could markedly alter the invasive properties of the melanoma cell panel in Matrigel^TM^, where PTEN^WT^ cells responded with significantly reduced invasive capacity and PTEN^Mut^ lines with enhanced invasion in response to WNT3A-activated signaling (Figure 2c and Supplementary Figure 2b).

Our *in vitro* invasion findings are supportive of previously described results from melanocyte-specific PTEN loss and BRAF^V600E^ mice, where stabilization or loss of β-catenin led to enhanced or reduced melanoma metastasis, respectively.^22^ However, it is currently unknown how WNT/β-catenin signaling can affect metastasis on a PTEN^WT^ background. To address this question, we performed intravenous injection of GFP-labeled, PTEN^WT^ WNT3A overexpression cells and monitored metastatic tumor formation in lung tissue. We found there was reduced tumor burden in the lungs of mice injected with the WNT3A overexpression cells compared with control cells, where distinct areas of metastatic-free tissue could be observed in the WNT3A animals compared with control lungs (Figure 2d). To confirm these findings, we used S100 as a melanoma marker in the lung tissue sections, to identify metastatic lesions (Supplementary Figure 2c). Importantly, quantification of the tumor burden in the lungs was found to be significantly reduced in the WNT3A overexpression treated animals (Figure 2e).

To confirm that these WNT/β-catenin-mediated effects on melanoma cell motility are PTEN dependent, we overexpressed PTEN in the mutant lines (A2058 and M229; Supplementary Figure 2d) and found that re-establishment of PTEN expression could the switch the effect of rWNT3A stimulation from pro- to anti-migratory (Figure 2f and Supplementary Figure 2e). PTEN is known to possess both PI3K-dependent and -independent activities.^44^ To investigate if the WNT/β-catenin-mediated effects on melanoma cell motility are PI3K dependent, we used the PI3K inhibitor, LY294002, in PTEN^Mut^ cells at a concentration that could inhibit PI3K-AKT signaling (Figure 2g), but could not significantly alter basal cell migration (Figure 2h). LY294002 blocks WNT3A-mediated increased cell migration in A2058 cells (Figure 2h), demonstrating that the effect of WNT/β-catenin signaling on melanoma cell motility is PI3K-dependent.

Using The Cancer Genome Atlas (TCGA) network cutaneous melanoma data set, we identified 209 patient samples with high *AXIN2* transcript expression (based on RNA-seq transcriptome analysis) as a marker of active WNT/β-catenin signaling and divided the samples into two cohorts as PTEN low or high expressing (*n*=47 and *n*=162, respectively; Supplementary Table S1), based on ~20% of uncultured tumors expected to have reduced PTEN expression.^31, 45, 46^ Analysis of the associated clinical data (Supplementary Table S1) showed that total and lymph node metastases in the PTEN high cohort is significantly reduced compared with PTEN low expressing tumors (Figure 2i). Distant organ metastases were not significantly different, albeit the number of patients presenting these lesions was low in this cohort (*n*=11; Figure 2h and Supplementary Table S1). Importantly, *AXIN2* low expressing patients (*n*=127) for which PTEN expression data were available, do not follow the same trend when subdivided into PTEN high (*n*=97) or low (*n*=30) cohorts (Supplementary Figure 2f and Supplementary Table S2), suggesting that this effect on metastasis is not because of a change in PTEN expression in isolation, but rather is specific to patients with high WNT/β-catenin signaling. Taken together, these data show that WNT/β-catenin signaling can reduce melanoma cell migration, invasion and metastasis in PTEN^WT^ cells, which is in contrast to the effects observed in PTEN^Mut^ cells.

### WNT3A regulates the bioenergetics of melanoma cells in a PTEN-dependent manner

**Figure 3:**
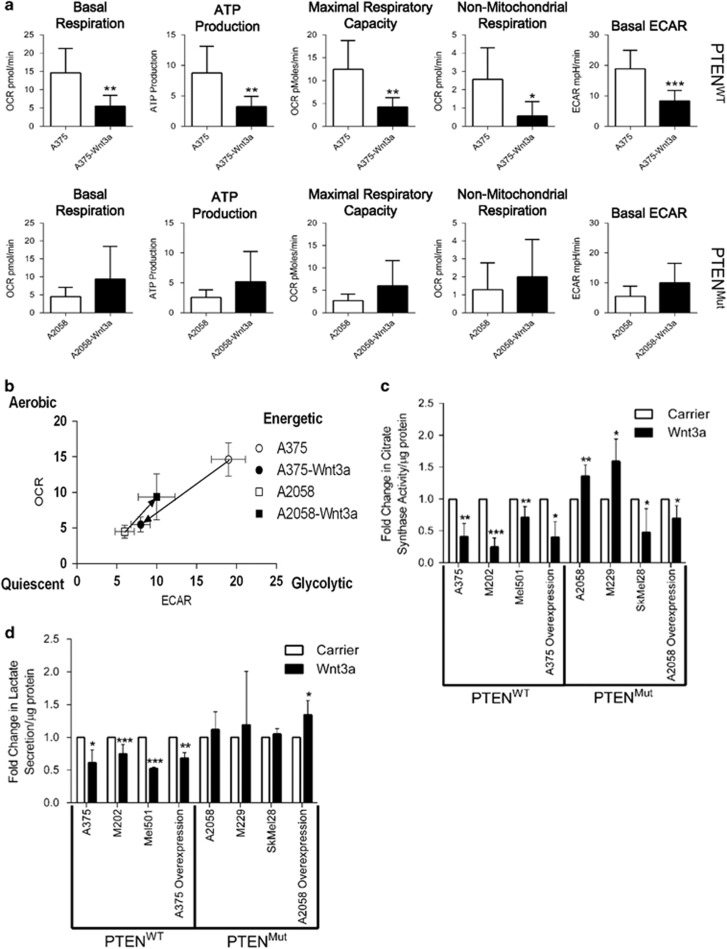
PTEN^WT^ cells have reduced metabolism in response to WNT/β-catenin signaling. (**a**) Bioenergetic metrics were calculated from the XF^e^96 extracellular flux bioanalyzer OCR and ECAR traces (see Supplementary Figure 3 for example traces). Upper panel PTEN^WT^ A375 cells, Lower panel PTEN^Mut^ A2058 cells. Basal respiration was calculated from the OCR data at 26 min, subtracting the non-mitochondrial respiration. ATP production was calculated by the change in OCR before and after injection of oligomycin. Maximal respiratory capacity was calculated after injection of FCCP, as the maximum OCR reading subtracting non-mitochondrial respiration. Non-mitochondrial respiration was calculated from the OCR data following injection of complex I and III inhibitors; antimycin A and rotenone, respectively. Basal ECAR was calculated from the ECAR data at 26 min, by subtracting the non-glycolytic acidification. Mean shown±s.d. *P<0.05 or **P<0.01 and ***P<0.001. (**b**) The shift in cellular energy phenotypes was calculated from basal OCR and basal ECAR readings for control and WNT3A overexpressing PTEN^WT^ A375, and PTEN^Mut^ A2058 cells. (**c**) Fold-change in CS activity normalized to total protein concentration. In parental lines, readings were taken following 48 h of stimulation with carrier or 50 ng/ml rWNT3A. (**d**) Fold-change in lactate secretion normalized to total protein concentration, using the same experimental conditions as described for **c**. For panels **c** and **d**, mean shown±s.d. *P<0.05 or **P<0.01 and ***P<0.001.

Given the previously identified role of WNT signaling in regulating cancer metabolism,^25^ we decided to investigate the bioenergetic properties of PTEN^WT^ and PTEN^Mut^ melanoma cells in response to WNT3A signaling. To test this, we used a Seahorse XF^e^96 extracellular flux analyzer to investigate whether oxygen consumption rates (OCRs) or extracellular acidification rates (ECARs) were affected by WNT3A, allowing us to study oxidative phosphorylation and indirectly also glycolytic rates in the melanoma cells, respectively. Using the overexpression melanoma cells, we found that both OCR and ECAR rates were altered in response to WNT3A signaling, which interestingly differed between the PTEN^WT^ and PTEN^Mut^ lines (Supplementary Figure 3). In A375 (PTEN^WT^) cells, WNT3A overexpression led to reduced rates of OCR and ECAR, which when analyzed for basic parameters of cell respiratory control,^47^ showed significantly reduced rates of basal respiration, ATP production, maximal respiratory capacity, non-mitochondrial respiration and basal ECAR (Figure 3a). In the PTEN^Mut^ A2058 cells, however, this reduction in cell respiratory control upon WNT3A stimulation was not detected (Figure 3a and Supplementary Figure 3). Overall, this represents a highly energetic to quiescent metabolic shift in the A375 cells in response to WNT3A, which is not observed in A2058 cells (Figure 3b).

To test whether the observations made using the XF^e^96 analyzer were due to differences in the way PTEN^WT^ and PTEN^Mut^ melanoma cells respond bioenergetically to WNT3A signaling, we analyzed metabolic responses to rWNT3A stimulation/WNT3A overexpression in the whole panel of melanoma cells. First, we measured citrate synthase (CS) activity, which is the initial enzyme of the tricarboxylic acid cycle, to determine mitochondrial and oxidative phosphorylation activity in the cells. In all the PTEN^WT^ cell types tested, CS activity was significantly reduced in response to hyperactive WNT3A signaling (Figure 3c). This was not reciprocated in all the in PTEN^Mut^ lines, which demonstrated unpredictable effects showing either hyper- or hypo-activity of CS in response to WNT3A treatment (Figure 3c). Next, we measured the glycolytic capacity of the melanoma cells using secreted lactate as a marker. In response to WNT3A, lactate secretion was significantly reduced in all the PTEN^WT^ lines tested (Figure 3d). Conversely this reduction was not observed in any of the PTEN^Mut^ cells when treated with rWNT3A (no significant effect detected), however, stable overexpression of WNT3A led to enhanced lactate secretion in these cells (Figure 3d). These data are consistent with the findings of the XF^e^96 analyzer (Supplementary Figure 3 and Figure 3a), showing that in response to enhanced WNT3A signaling, PTEN^WT^ melanoma cells exhibit significantly reduced cellular metabolism, including both oxidative phosphorylation and glycolytic capacity. However, this is not the case for PTEN^Mut^ melanoma cells, which do not respond consistently to WNT3A-mediated metabolic reprogramming. Taken together, these data show that melanoma cells respond to WNT3A signaling with differing metabolic signatures based on PTEN expression, which for the PTEN^WT^ cells results in significantly reduced cellular metabolism.

### WNT3A remodels mitochondria in melanoma cells in a PTEN-dependent manner

**Figure 4:**
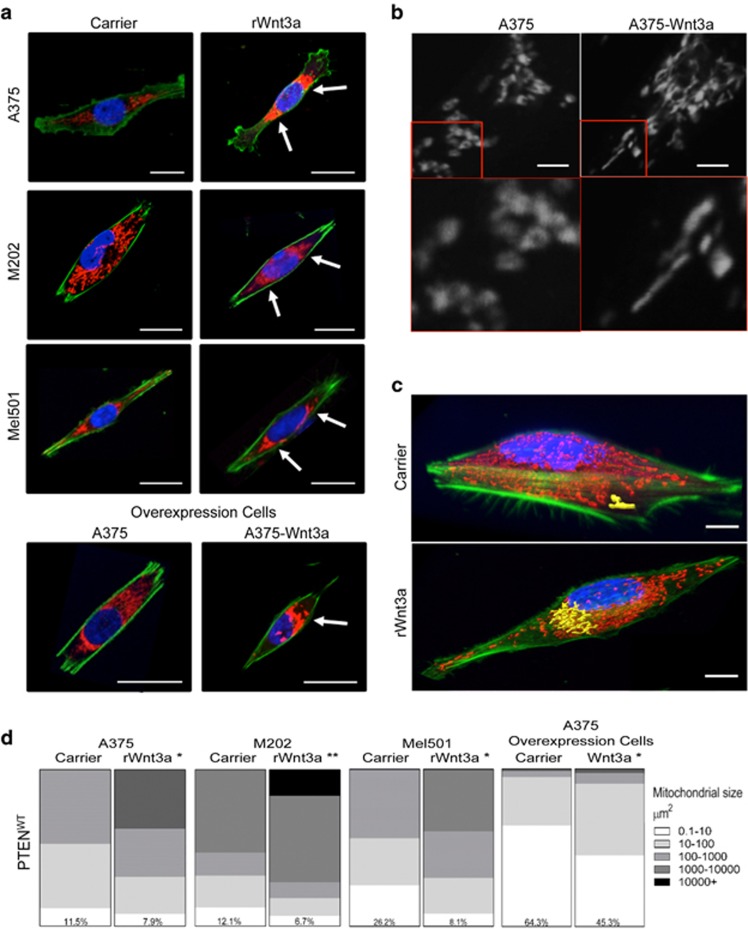
PTEN^WT^ melanoma cells exhibit increased mitochondrial networking in response to WNT3A signaling. (**a**) Representative images of individual PTEN^WT^ melanoma cells stained with; MitoTracker Deep Red (red), phalloidin (green) and Hoechst (blue). Arrows highlight peri-nuclear clustering of mitochondria. Scale bar, 50 μm. (**b**) Cells stained with MitoTracker Deep Red (250 nM) were imaged at high power (63 × ; upper panel). Scale bar, 5 μm. Lower panel; zoomed-in images (150 ×) of mitochondria from red inset square as shown in upper panel images. (**c)** Representative 3D images of Imaris (Bitplane AG, Zurich, Switzerland) analysis of M202 cells with example mitochondrial networks highlighted in yellow. Cells were stained with the following markers: MitoTracker Deep Red (red), phalloidin (green) and Hoechst (blue). Scale bar, 15 μm. (**d**) Quantified Imaris data for PTEN^WT^ melanoma cells. Numbers in each chart represent the percentage of mitochondria that range in size from 0.1 to 10 μM^2^ for each treatment. *P<0.05 or **P<0.01.

It is now known that adaptations of bioenergetic activity and mitochondrial-derived ATP production, results in the remodeling of mitochondrial morphology, as previously reviewed.^48^ Given the metabolic-induced effects of WNT3A in melanoma cells (Figure 3), we decided to investigate mitochondrial architecture in these cells using the cell-permeant, MitoTracker mitochondrion-selective probe (Invitrogen, Life Technologies Ltd, Paisley, UK). This led us to the intriguing observation that in PTEN^WT^ melanoma cells in response to WNT3A, the mitochondria amassed in dense peri-nuclear clusters (Figure 4a), which was not apparent in the PTEN^Mut^ cells (Supplementary Figure 4a). Higher power images of mitochondrial regions in PTEN^WT^ melanoma cells in response to forced WNT3A signaling showed that the mitochondria appeared to be elongated in shape compared with the control cells (Figure 4b). To quantify this, we generated Z-stacks across the depth of the cells, to provide 3D imaging of mitochondrial morphology and used Imaris software (Bitplane AG, Zurich, Switzerland) to measure the surface area of interconnected structures (Figure 4c). This showed that in PTEN^WT^ cells there was a significant increase in mitochondrial sizes in response to WNT3A signaling (Figure 4d), suggesting that the dense mitochondrial staining observed in the peri-nuclear region of these cells (Figure 4a) is composed of larger mitochondrial networks. Interestingly, we did not see this effect in PTEN^Mut^ cells, where there was no significant effect of WNT3A on mitochondrial networking (Supplementary Figure 4b). These results showed that in PTEN^WT^ melanoma cells, WNT3A signaling remodels mitochondrial morphology, which does not occur in PTEN^Mut^ cells.

### WNT3A-mediated metabolic and mitochondrial effects are β-catenin-dependent

**Figure 5:**
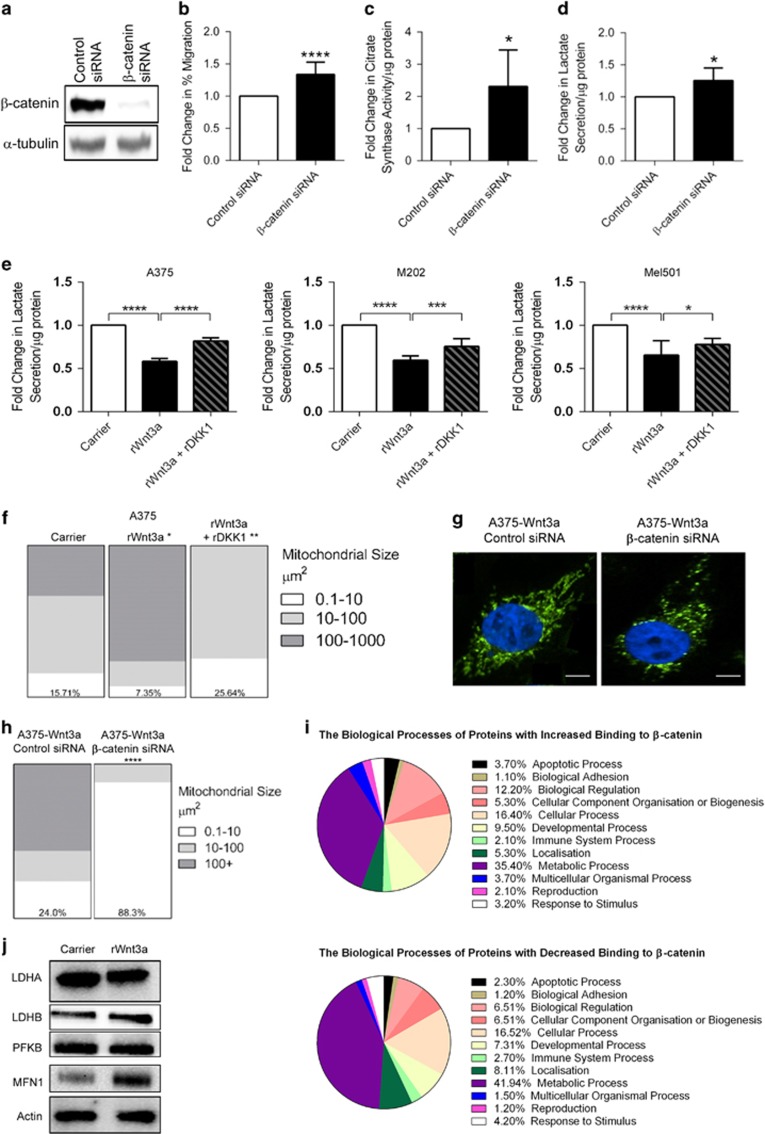
WNT3A-mediated effects in PTEN^WT^ melanoma cells are β-catenin dependent. (**a**) Western blot analysis of β-catenin expression levels in A375 WNT3A overexpression cells following transfection with β-catenin siRNA or scrambled control siRNA for 72 h. α-tubulin served as the loading control. (**b**) Fold-change in cell migration in A375 WNT3A overexpression cells treated with scrambled or β-catenin siRNA. (**c**) Fold-change in CS activity in A375 WNT3A overexpression cells treated with scrambled or β-catenin siRNA. (**d**) Fold-change in lactate secretion from A375 WNT3A overexpression cells treated with scrambled or β-catenin siRNA, normalized to total protein concentration. (**e**) Fold-change in lactate secretion from PTEN^WT^ cells treated with rWNT3A (50 ng/ml)±rDKK1 (50 ng/ml) or carrier treatment, normalized to total protein concentration. (**f)** Quantified Imaris data for A375 overexpression cells treated with rWNT3A (50 ng/ml)±rDKK1 (50 ng/ml) or carrier control. Numbers in each chart represents the percentage of mitochondria that range in size from 0.1 to 10 μM^2^ for each treatment. For panels (**b**–**f**), mean shown±s.d. *P<0.05, **P<0.01, ***P<0.001 and ****P<0.0001 (as compared with control). (**g**) A375 overexpression cells stained with MitoTracker Green (10 nM; green) and counterstained with Hoechst (blue). siRNA treatment as described above and in main text. Example images shown. Scale bar, 10 μm. (**h**) Quantified Imaris data for A375 overexpression cells transfected with β-catenin siRNA or a scrambled control siRNA. Numbers in each chart represent the percentage of mitochondria that range in size from 0.1 to 10 μM^2^ for each treatment. ****P<0.0001. (**i)** PANTHER analysis of proteins identified by MS-IP with increased (upper panel) and decreased (lower panel) binding to β-catenin. See main text for experimental detail of the MS-IP approach used. (**j**) Western Blot for enzymes identified by MS-IP analysis; LDHA, LDHB, PFKB. MFN1 was used as a positive control (see later text and Figure 7). Parental lines were stimulated with carrier or 50 ng/ml rWnt3a, 48 h before analysis.

Findings from our reporter assay experiments suggest that in melanoma cells, WNT3A is signaling in a β-catenin-dependent manner (Figures 1d and f). Given that this is consistent with previous findings,^15^ we hypothesized that the novel metabolic and mitochondrial effects we have noted in PTEN^WT^ melanoma cells in response to WNT3A signaling, is dependent upon β-catenin activity. We therefore examined the effects of transient knockdown of β-catenin in PTEN^WT^ WNT3A overexpression melanoma cells using small interfering RNA (siRNA) and found efficient reduction of *CTNNB1* gene expression in response to treatment (Figure 5a). To ensure this knockdown approach was able to reduce β-catenin signaling, we also confirmed that the WNT/β-catenin target gene, *AXIN2,*^49^ was effectively reduced (Supplementary Figure 5a).

Using this β-catenin knockdown approach, we began to evaluate whether we could confirm some of our previous findings made using hyperactive WNT3A signaling, starting with cell migration in the PTEN^WT^ melanoma cells. This showed that as expected, reduced β-catenin expression led to increased migration of the PTEN^WT^ cells (Figure 5b). Furthermore, a reduction in β-catenin expression could significantly increase cellular metabolism in the PTEN^WT^ melanoma cells as demonstrated by increased CS activity (Figure 5c) and lactate secretion (Figure 5d), compared with control siRNA-treated cells. In addition to its role in WNT/β-catenin signaling, β-catenin also constitutes part of the cell–cell adhesion adherens junction complex,^50^ which may also be effected by knockdown in *CTNNB1* expression. To rule out that the metabolic and mitochondrial changes induced by β-catenin knockdown may be due to a depletion of the adherens junction pool, we used recombinant Dickkopf WNT signaling pathway inhibitor-1 (rDKK1), which is a well-known secreted inhibitor of the WNT/β-catenin signaling pathway.^51^ By forming a ternary complex with LRP5/6 along with another receptor, Kremen, DKK1 stimulates the endocytic removal of LRP5/6 from the cell surface thereby preventing WNT ligand-mediated stimulation of the WNT/β-catenin pathway.^52^ We found that rDKK1 treatment of WNT3A-stimulated A375 (PTEN^WT^) melanoma cells not only inhibited WNT/β-catenin signaling (Supplementary Figure 5b), but importantly could also elevate WNT3A-mediated reduction in lactate secretion in PTEN^WT^ melanoma cells (Figure 5e).

Next, we evaluated whether inhibition of β-catenin signaling resulted in mitochondrial remodeling in the PTEN^WT^ melanoma cells. Using the MitoTracker imaging and Imaris software analysis (as previously shown in Figure 4 and Supplementary Figure 4c), we found that rDKK1 blocked WNT3A-mediated increased mitochondrial networking (Figure 5f) and that knockdown of β-catenin led to fragmentation of the mitochondrial network (Figure 5g), which resulted in a marked increase in the number of mitochondria that ranged in size from 0.1 to 10 μM^2^, compared with scrambled siRNA-treated cells (Figure 5h). Taken together, these finding show that β-catenin signaling is essential for mitochondrial remodeling induced by WNT3A (Figure 4), but also for the WNT3A-mediated bioenergetic reprogramming of PTEN^WT^ melanoma cells (Figure 3).

To investigate how β-catenin regulates these processes, we assessed target proteins in the cells that bind β-catenin in the absence and presence of the WNT3A ligand, using a proteomics approach of immunoprecipitation followed by mass spectrometry. We unbiasedly analyzed proteins with increased and decreased binding to β-catenin in WNT3A PTEN^WT^ overexpression cells compared with control cells. Immunoprecipitated proteins were digested and the corresponding peptides were quantified using label-free methods,^53^ a method that was demonstrated to be highly reproducible (Supplementary Figure 5c). This immunoprecipitation mass spectrometry approach showed that in melanoma cells, the majority of β-catenin-binding proteins compared with the IgG control were involved in metabolic processes (Figure 5i), but the composition of the metabolism proteins were vastly altered upon WNT3A-mediated stabilization of β-catenin (Supplementary Tables S3 and S4). For example, a number of key glycolysis enzymes exhibit reduced binding to β-catenin in the presence of WNT3A ligand stimulation (including glyceraldehyde-3-phosphate dehydrogenase, lactate dehydrogenase, phosphofructokinase-1 and glucose-6-phosphate isomerase; Supplementary Table S4), which suggests that upon WNT/β-catenin signaling in PTEN^WT^ melanoma cells, β-catenin no longer binds to glycolysis enzymes, which may lead to metabolic reprogramming events such as reduced glycolytic capacity. This is supported by the finding that a representative number of these metabolic enzymes do not alter upon rWNT3A treatment, suggesting that the observed decrease in β-catenin binding is unlikely to result from the downregulation of total enzyme levels (Figure 5j). Overall, these findings indicate that melanoma cells expressing high levels of PTEN respond to WNT3A signaling with metabolic reprogramming and mitochondrial remodeling, which is dependent upon β-catenin activity.

### WNT/β-catenin signaling does not alter mitochondrial numbers in PTEN^WT^ melanoma cells, but can increase mitochondrial membrane potential

**Figure 6:**
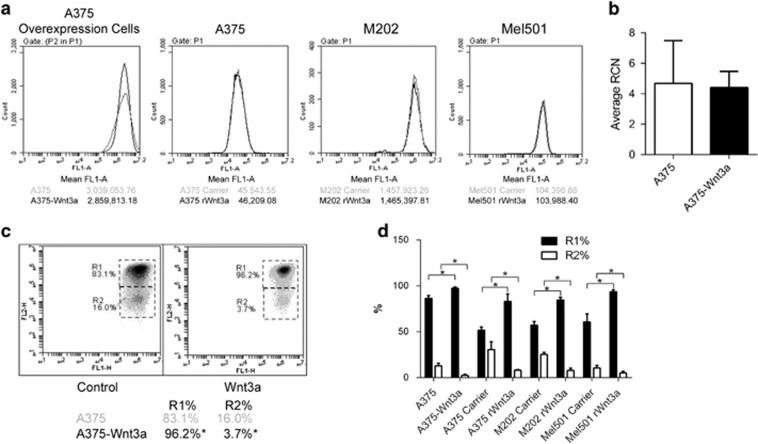
WNT3A does not alter mitochondrial numbers in PTEN^WT^ melanoma cells, but does increase ΔΨm. (**a**) Flow cytometric analysis of MitoTracker Green staining in PTEN^WT^ melanoma cells. Parental lines were stimulated with carrier or 50 ng/ml rWNT3A, 48 h before analysis. (**b**) Total mitochondrial amount on A375 overexpression cells was monitored by analyzing mitochondrial DNA content normalized to nuclear DNA using PCR, to provide a relative copy number (RCN) ratio. Mean shown±s.d. (**c**) JC-1 analysis of ΔΨm in PTEN^WT^ overexpression cells or following stimulation with carrier control or rWNT3A (50 ng/ml) for 48 h. (**d**) Percentage change in JC-1 expression levels in gates R1 and R2 in PTEN^WT^ overexpression cells or following stimulation with carrier control or rWNT3A (50 ng/ml) for 48 h. Mean shown±s.d. *P<0.05.

In murine myoblasts, WNT3A/β-catenin signaling has previously been shown to increase mitochondrial biogenesis,^54^ therefore it is reasonable to presume that the mitochondrial effects noted in PTEN^WT^ melanoma cells in response to hyperactive WNT/β-catenin signaling (Figures 4 and 5), could be due to alterations in mitochondrial numbers in these cells. To test this, we first used MitoTracker dye and quantitatively analyzed uptake in the PTEN^WT^ melanoma cells using flow cytometry as an indication of the total amount of mitochondria in the cells, however, we did not detect a difference in cells either treated with rWNT3A or overexpressing WNT3A, compared with control-treated cells (Figure 6a). These results suggest that the total number of mitochondria is not affected by WNT/β-catenin signaling in PTEN^WT^ melanoma lines. To confirm this, we analyzed mitochondrial DNA copy number and found no increase in response to hyperactive WNT3A signaling (Figure 6b). As expected nor did we see an effect of WNT3A signaling on mitochondrial content in PTEN^Mut^ melanoma cells (Supplementary Figure 6a). Taken together, these results show that WNT/β-catenin signaling does not increase mitochondrial biogenesis in these cells and the effects noted on increased mitochondrial networking in PTEN^WT^ melanoma lines (Figures 4 and 5) are independent of changes in total mitochondrial numbers.

Despite not affecting total mitochondrial content, we did find that WNT3A was capable of altering the mitochondrial membrane potential (ΔΨm) of PTEN^WT^ melanoma cells, using fluorescent probes (Figures 6c and d and Supplementary Figure 6b). Specifically, WNT3A signaling was able to increase the population of mitochondria exhibiting increased levels in ΔΨm compared with control A375 cells, using both the ΔΨm-dependent JC-1 (Figures 6c and d) and TMRM (Supplementary Figure 6b) fluorescence indicators. Changes in ΔΨm are closely linked to mitochondrial morphology, therefore these results suggest that the increase in mitochondrial networking induced by WNT/β-catenin signaling in PTEN^WT^ cells (Figures 4 and 5) could be related to the increase in ΔΨm also noted.

### Mitochondrial dynamics and mitophagy are regulated by WNT/β-catenin signaling in PTEN^WT^ melanoma cells

**Figure 7:**
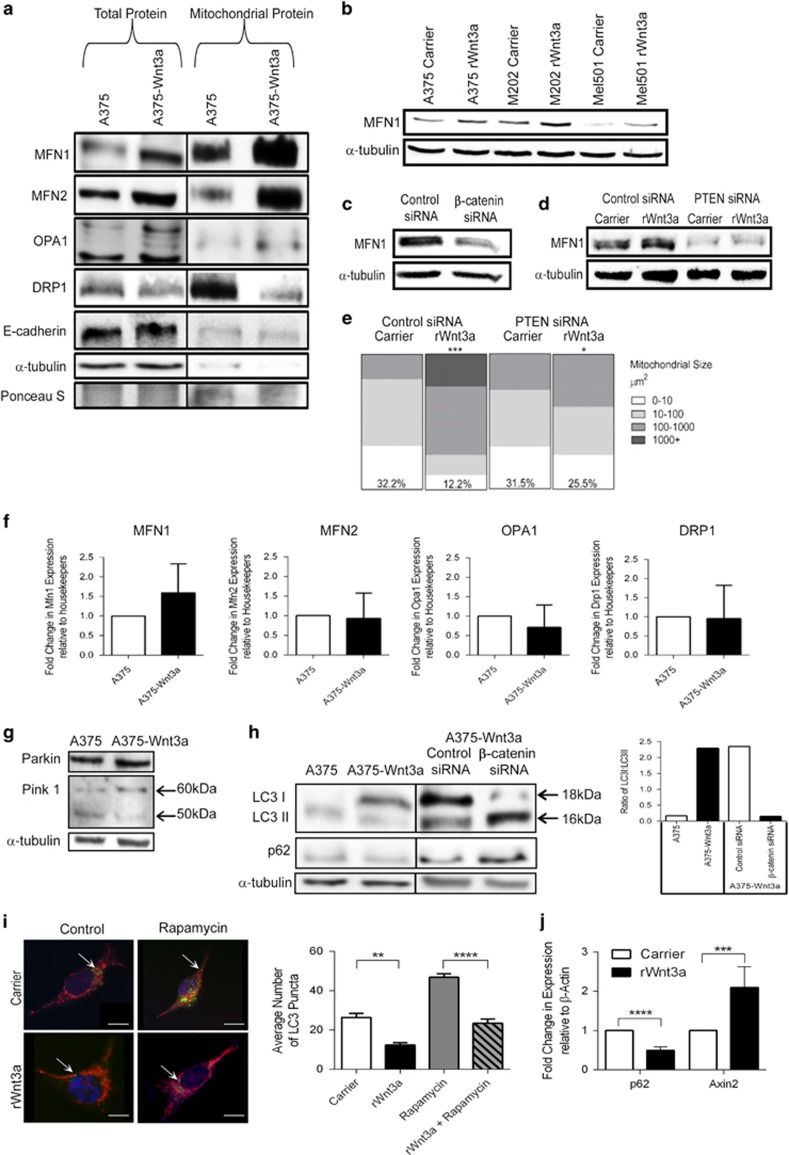
WNT/β-catenin signaling regulates mitochondrial dynamics machinery in PTEN^WT^ melanoma cells. (**a**) Western blot for mitochondrial dynamic genes, MFN1, MFN2, OPA1 and DRP1 in total protein extracts and mitochondrial sub-fractions of A375 control and WNT3A overexpression cells. E-cadherin and α-tubulin were used to judge the purity of the mitochondrial extraction, whereas Ponceau staining was used to judge equal loading across all sample sets. (**b**) Western blot analysis of MFN1 expression in PTEN^WT^ melanoma cells stimulated with carrier control or rWNT3A (50 ng/ml) for 48 h. Densitometry data for this blot are shown in Supplementary Figure 7a. (**c**) Western blot analysis of MFN1 in A375 WNT3A overexpression cells treated with scrambled and β-catenin siRNA for 72 h. (**d**) Western blot analysis of MFN1 expression in A375 cells treated with scrambled and PTEN siRNA for 72 h followed by stimulation with carrier control or rWNT3A (50 ng/ml) for 48 h. (**e**) Quantified Imaris data of mitochondrial size in A375 cells transfected with PTEN siRNA or a scrambled control siRNA for 72 h before stimulation with carrier control or rWNT3A (50 ng/ml) for 48 h. Numbers in each chart represents the percentage of mitochondria that range in size from 0.1 to 10 μM^2^ for each treatment. *P<0.05 and ***P<0.001 (as compared with control). (**f**) Reverse transcription (RT)–quantitative PCR (qPCR) for *MFN1*, *MFN2*, *OPA1* and *DNM1L* (encoding for DRP1) transcripts in A375 control and WNT3A overexpression cells. mRNA expression levels were normalized based on the expression of three housekeepers, *YWHAZ*, *UBC* and *ACTB*. (**g**) A375 control and WNT3A overexpression cells were analyzed by western blot for Parkin and PINK1 expression. FL-PINK1 indicted by the 60 kDa band and the cleaved isoform by the 50 kDa band. (**h**) A375 control and WNT3A overexpression cells were analyzed by western blot for autophagy markers LC3 and p62 (left panel), and following treatment with β-catenin or scrambled siRNA (right panel). For all western blot panels, α-tubulin served as the loading control. Graph shows the ratio of LC3I (18 kDa) to LC3II (16 kDa) in total protein and following treatment with β-catenin or scrambled siRNA in A375 control and WNT3A overexpression cells (calculated from data shown). (**i**) Representative images of A375 cells following stimulation with carrier control or rWnt3a (50 ng/ml) and Rapamycin (as a positive control^103^; 200 nM) or control for 48 h. Cells stained with; MitoTracker Deep Red (red), LC3 (green) and Hoechst (blue). Scale bar, 50 μm. Graph shows the number of LC3 puncta, mean shown±s.d. **P<0.01 and ****P<0.0001. (**j**) Reverse transcription (RT)–quantitative PCR (qPCR) for *SQSTM1* (encoding p62) and *AXIN2* transcripts in A375 cells following stimulation with carrier control or rWnt3a (50 ng/ml) for 48 h. mRNA expression levels were normalized based on the expression of the housekeeper *ACTB*. ***P<0.001 and ****P<0.0001.

Given that the WNT/β-catenin signaling pathway increased mitochondrial networking in PTEN^WT^ melanoma cells (Figures 4 and 5), we hypothesized that the mitochondrial fusion and fission mechanisms (the so-called mitochondrial dynamics machinery) could be regulated to permit increased networks. Mitochondria cycle through repetitive cycles of fusion and fission that determines the architecture of the mitochondrial network, to ultimately influence every aspect of mitochondrial function (including intermediary metabolism), where these processes are mediated by a number of GTPase pro-fusion (Mitofusin-1 (MFN1), Mitofusin-2 (MFN2) and Optic atrophy-1 (OPA1)), and pro-fission (Dynamin-1-like protein (DRP1) and Mitochondrial fission-1 (FIS1)) proteins.^55^ We tested whether hyperactive WNT/β-catenin signaling was capable of regulating expression of some of these mitochondrial dynamics proteins in PTEN^WT^ melanoma cells and found that it increased expression of pro-fusion proteins, whereas concomitantly reducing the pro-fission, DRP1 levels (Figures 7a–c and Supplementary Figure 7a). As increased expression of mitochondrial fusion proteins MFN1, MFN2 and OPA1 would explain why mitochondrial networks are increased in PTEN^WT^ melanoma cells, we verified their increased expression in response to WNT/β-catenin signaling by immunofluorescence (Supplementary Figure 7b) and flow cytometry (Supplementary Figure 7c) to further corroborate our findings.

As we were unable to detect increased mitochondrial networking in PTEN^Mut^ melanoma cells in response to WNT/β-catenin signaling (Supplementary Figure 4), we hypothesized that reduction of PTEN in PTEN^WT^ cells (Supplementary Figure 7d) would block WNT3A-mediated mitochondrial fusion. Indeed transient knockdown of PTEN in PTEN^WT^ melanoma cells reduced MFN1 expression, thereby antagonizing WNT3A-mediated increased MFN1 expression and of note PTEN depletion appears to reduce MFN1 expression in these cells (Figure 7d). Crucially PTEN knockdown also antagonized increased mitochondrial networking in response to WNT/β-catenin signaling, in PTEN^WT^ melanoma cells (Figure 7e). We were unable to identify altered mRNA expression levels of mitochondrial dynamics genes in PTEN^WT^ cells in response to hyperactive WNT/β-catenin signaling (Figure 7f), suggesting that rather than being transcriptionally regulated by β-catenin signaling, expression levels of these proteins are regulated in a post-transcriptional manner.

Parkin is a RING-between-RING-type E3 ubiquitin ligase involved in the mitophagy quality control pathway to remove damaged mitochondria, which ubiquitinates multiple outer mitochondrial membrane (OMM) proteins (marking them for degradation) including MFNs^56^ and is targeted to depolarized mitochondria by PTEN-induced putative kinase-1 (PINK1). We investigated PINK1-Parkin accumulation in PTEN^WT^ melanoma cells in response to WNT/β-catenin signaling to determine whether impairment of either could lead to increased levels of MFN proteins and found that while differences in Parkin levels were modest (slightly increased in response to WNT3A signaling; Figure 7g and Supplementary Figure 7e; which fits with the recent finding that WNT3A increases endogenous Parkin in glioblastoma cells^57^), we did detected a marked difference in PINK1 expression (Figure 7g). PINK1 undergoes voltage-dependent proteolysis in healthy mitochondria, but is stabilized in a full-length (FL) form on the OMM of damaged mitochondria,^58^ where we found that WNT3A increased FL-PINK1 expression (Figure 7g), suggesting that PTEN^WT^ melanoma cells harbor defective mitochondria in response to WNT/β-catenin signaling that are tagged for selective autophagy (mitophagy). Importantly, the uncoupler, FCCP (which dissipates ΔΨm), also stabilizes FL-PINK1 in PTEN^WT^ melanoma cells, demonstrating that these cells are capable of proteolytic cleaving of PINK1 (Supplementary Figure 7f). Further investigations demonstrated that autophagy markers are markedly reduced in PTEN^WT^ melanoma cells in response to hyperactive WNT3A signaling (Figure 7h), but not in PTEN^Mut^ cells (Supplementary Figure 7g) suggesting that autophagy (and thus the mitophagy process), is impaired in PTEN^WT^ melanoma cells in response to WNT/β-catenin signaling. This has been further corroborated by analyzing LC3 punctae staining in the presence of an autophagy inducer and found that WNT3A can significantly reduce autophagosome function in PTEN^WT^ melanoma cells (Figure 7i). WNT/β-catenin signaling has previously been found to be a negative regulator of autophagy, by repressing p62 (encoded by *SQSTM1*) expression, which is a protein that targets cargos for autophagy.^59^ Importantly, we also detect repression of *SQSTM1* expression in PTEN^WT^ melanoma cells following treatment with rWNT3A (Figure 7j).

**Figure 8:**
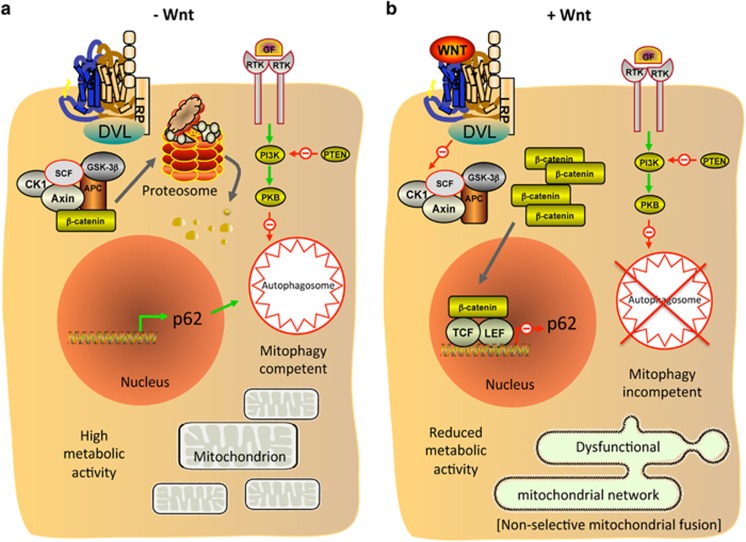
Working model portraying the effects of WNT/β-catenin signaling in PTEN^WT^ melanoma cells under nutrient starvation conditions. (**a**) In the absence of a WNT ligand that signals via the WNT/β-catenin pathway, β-catenin is targeted by a destruction complex (comprising CK1, SCF, GSK-3β, AXIN and APC) for proteasome-mediated degradation. Under nutrient starvation conditions (such as in large solid tumors), PTEN drives increased autophagy. PTEN blocks PI3K-mediated activation of AKT (PKB), where AKT negatively regulates autophagy in response to mitogens (in reality occurring through activation of mTOR; not shown). This triggers autophagy addiction in PTEN expressing tumors, which does not occur in PTEN^Mut^ melanomas. (**b**) In response to a WNT/β-catenin signaling ligand, the seven-transmembrane domain FZD receptors (blue/brown color in schematic) and LRP5/6 receptor complex, results in activation of DVL, which in turn inactivates the destruction complex. Cytoplasmic β-catenin then accumulates and is eventually translocated to the nucleus, where it interacts with transcription factor/lymphoid enhancer-binding element transcription factors to activate/repress expression of WNT target genes including *SQSTM1* (gene encoding p62), which is transcriptionally repressed in response to β-catenin signaling. p62 is an autophagy adaptor that facilitates the autophagic degradation of ubiquitinated protein aggregates. We propose that inhibition of autophagy by WNT/β-catenin signaling in autophagy addicted cancer cells, inhibits mitophagy leading to nonselective mitochondrial fusion. This results in a highly networked, yet dysfunction mitochondrial population with significantly reduced cellular metabolic capacity, which negatively affects tumor cell behavior such as motility and metastasis.

Overall, our findings show that cells harboring deletion mutations of PTEN increase their invasive capacity in response to WNT/β-catenin signaling, whereas PTEN^WT^ cells exhibit a significant reduction in invasion and metastasis (Figure 2), the latter of which is accompanied by a marked reduction in cellular metabolism (Figure 3) and mitochondrial remodeling (Figure 4). This remodeling involves the formation of fused networks of mitochondria, which is achieved by increased expression of mitochondrial fusion and reduced fission proteins, resulting from reduced mitophagy (Figure 7). We propose a model whereby WNT/β-catenin signaling in PTEN^WT^ melanoma cells leads to the accumulation of MFNs on damaged mitochondria, directing them toward reengagement in fusion events (nonselective mitochondrial fusion) due to defective mitophagy (Figure 8). This will likely lead to the accumulation of damaged mitochondria through nonselective fusion with limited fission events, leading to a static hyperfused mitochondrial state that has previously been associated with reduced mitochondrial respiration,^60^ a situation that could ultimately affect cellular activities such as motility.

## Discussion

In melanoma, the role of the WNT/β-catenin pathway is controversial,^15, 18, 22, 42, 61, 62, 63^ where differing tumor mutational status may account for these discrepancies. Indeed, β-catenin signaling has been shown to increase or repress melanoma cell invasion depending on the cell line tested.^18, 42^ Here we provide evidence that a reduction in PTEN expression in melanoma cells markedly alters their phenotypic response to WNT/β-catenin signaling by reprogramming distinct metabolic signatures, which is underpinned by changes in mitochondrial activity in PTEN^WT^, but not in PTEN^Mut^ cells.

PTEN can be frequently lost in melanoma,^64^ where it functions as a tumor suppressor to negatively regulate PI3K-AKT signaling through the dephosphorylation of the plasma membrane bound phospholipid, phosphatidylinositol-3,4,5-trisphosphate, which functions downstream of PI3K to activate AKT. Increased AKT activity promotes glycolysis through multiple mechanisms, including upregulated glucose transport^65, 66^ and increased hexokinase activity.^67, 68^ Conversely, PTEN can promote oxidative phosphorylation and reduce glycolysis,^69^ suggesting that tumor cells harboring loss of or reduced PTEN expression will have markedly differing metabolic profiles to cells without PTEN loss. We have previously reviewed the critical role WNT signaling has in cancer cell metabolism,^25^ so our current finding that WNT/β-catenin signaling imparts altered metabolic profiles in melanoma cells dependent on PTEN expression levels, demonstrates that cross-talk between the PI3K-AKT and WNT/β-catenin pathways exists to induce metabolic reprogramming in tumor cells.

Hyper-activation of the mitogen-activated protein kinase signaling pathway through constitutively activating mutations in BRAF or NRAS causes oncogene-induced senescence in melanocytes,^70, 71^ which can be circumvented by the further acquisition of genes that allow for bypass of the senescence program, including PTEN and β-catenin;^71, 72^ albeit NRAS and PTEN mutations have been suggested to be mutually exclusive as oncogenic RAS can activate the PI3K-AKT pathway in melanoma.^73^ This is unlike activating mutations in BRAF, which are frequently found co-occurring with loss of PTEN in melanoma.^74^ Similar to PTEN loss, activation of β-catenin also occurs in melanoma, where nuclear β-catenin has been reported in a third to two-thirds of all tumors,^75, 76^ suggesting it may have a role in tumor progression (at least in some patients). Our findings show that the effects of β-catenin signaling in melanoma cells differs depending on PTEN expression; in the absence of PTEN loss, cellular bioenergetics is compromised by β-catenin and tumor invasion/metastasis reduced, whereas cells exhibiting reduced expression of PTEN have increased invasion in response to β-catenin signaling, which does not appear to be due to a definable metabolic reprogramming event in these cells, suggesting the pro-invasive effects of WNT/β-catenin signaling are potentially independent of metabolic reprogramming in this particular context. Our findings are supported by a transgenic mouse model, where stabilized β-catenin combined with PTEN loss and activating BRAF^V600E^ mutation in cells of melanocytic origin, resulted in increased metastasis.^22^ β-Catenin stabilization in this model was recently shown to block T-cell recruitment to the tumors, providing resistance to immunotherapies and showing that WNT/β-catenin signaling can promote progression of melanoma through a variety of mechanisms in this specific mutational background,^77^ where (unlike in melanoma cells expressing PTEN) maintenance of high metabolic activity would be essential for WNT/β-catenin signaling to promote tumor progression in this context.

PTEN antagonizes nuclear accumulation of β-catenin,^78, 79^ suggesting that in PTEN^WT^ melanoma cells, WNT/β-catenin signaling may be restricted compared with the PTEN^Mut^ cells, which may explain the phenotypic differences observed between the two cell types. However, the WNT/β-catenin pathway is not blocked in PTEN^WT^ melanoma cells and can still be strongly activated by exogenous WNT treatment (Figures 1c and d), demonstrating that the pathway is at least partially functional in the cell lines tested here. Another possible explanation for the differing effects of WNT/β-catenin signaling on cell migration and invasion of PTEN^Mut^ and PTEN^WT^ melanoma cells, could be a difference in expression of the Microphthalmia-associated transcription factor (MITF), which is a target of WNT/β-catenin signaling and can regulate aggressive behavior in melanoma cells.^80, 81^ However, we were unable to identify differences in expression of MITF between the PTEN^Mut^ and PTEN^WT^ melanoma cells (data not shown) and inhibition of cell migration by β-catenin has only been shown to be partially dependent on MITF in melanocyte lineage cells,^61^ suggesting that differing MITF expression levels are unlikely to account for the difference in cell migration and invasion noted here for the PTEN^Mut^ and PTEN^WT^ melanoma cells in response to WNT/β-catenin signaling. Rather, we suggest that the differences in cell motility identified between these two cells types are due to a fundamental change induced by the WNT/β-catenin pathway on the metabolic status of the melanoma cells.

In melanoma, mitochondria remain functional^82, 83^ and we have discovered distinct mitochondrial activity between PTEN^WT^ and PTEN^Mut^ cells in response to WNT/β-catenin signaling. Specifically, we found that in PTEN^WT^ melanoma cells, WNT/β-catenin signaling increases mitochondrial networking by upregulating expression of mitochondrial fusion proteins and concomitant downregulation of mitochondrial fission machinery, indicating that this pathway is an important regulator of mitochondrial dynamics at least in a subset of melanoma cells. Interestingly, this WNT pathway is not the only one that can control mitochondrial morphology, as the WNT5A/Ca^2+^ signaling pathway has also been found to modulate mitochondrial dynamics in normal cells by promoting fission^84, 85, 86^ (at least in part through DRP1 activation^84^). However, in these same cells, the WNT/β-catenin pathway was unable to regulate mitochondrial aggregation, failing to alter mitochondrial morphology nor antagonize WNT/β-catenin-independent-mediated mitochondrial fission,^85, 86^ highlighting the context-dependent nature of the WNT signaling network on mitochondrial dynamics. It is tempting to speculate that the effects of the WNT/β-catenin pathway in regulating mitochondrial dynamics in PTEN^WT^ melanoma cells, may highlight a potentially novel tumor-specific role of this pathway in certain cancer contexts. It remains to be determined which additional tumor types may also be affected, however, other cancer cells that exhibit augmented metabolic profiles in response to WNT/β-catenin signaling, such as colorectal cancer,^27^ represent a logical extension to further such studies.

PINK1-Parkin signaling represents a mitochondrial quality control pathway that is able to tag defective mitochondria for selective degradation by the autophagosomal machinery, where the current model states that PINK1 is selectively stabilized on the OMM of defective mitochondria, facilitating Parkin recruitment to poly-ubiqutinated OMM proteins (including MFN1 and MFN2) and isolate defective mitochondria in the autophagosome.^56, 87, 88, 89, 90, 91, 92^ In this model, PINK1 is turned over by proteolysis in well-coupled mitochondria, but when the ΔΨm drops, FL-PINK1 is stabilized on the OMM, allowing for selective accumulation on impaired mitochondria and marking these organelles for degradation.^93^ However, our data contrast with this current model where we detected high expression of FL-PINK1 in response to WNT/β-catenin signaling in PTEN^WT^ cells, despite a concomitant increase in ΔΨm (which may be effected by altered mitochondrial dynamics). Interestingly there are a couple of alternative, ΔΨm-independent mechanisms can also result in FL-PINK1 accumulation, which include overwhelming of the mitochondrial import channels with excess PINK1 and preventing PINK1 cleavage by reducing mitochondrial protease activity.^93, 94^ One of these or an alternative mechanism could be responsible for the increased FL-PINK1 levels observed in PTEN^WT^ melanoma cells in response to WNT/β-catenin signaling. Mitophagy is defined as the degradation of mitochondria through the macroautophagic pathway, thus defects in the autophagic machinery inhibit mitophagy. We found an increase in p62 protein following β-catenin knockdown and β-catenin-mediated repression of the *SQSTM1* transcript, as well as increased autophagosome numbers (Figure 7), highlighting p62 regulation as a WNT/β-catenin-mediated mitophagy regulation mechanism in PTEN^WT^ melanoma cells (Figure 8). However, we cannot rule out that another autophagy regulator is also involved, given that p62 accumulation itself is also a marker of autophagy.^95^ Interestingly, DKK3 (a WNT/β-catenin signaling regulator) was recently found to be a marker of autophagy in melanoma, suggesting that inhibition of WNT/β-catenin signaling could increase autophagy in melanoma cells.^96^

Our model (Figure 8) can also explain why this mitophagy-dependent phenotype is not observed in PTEN^Mut^ melanoma cells: It has long been known that PTEN can increase autophagy by inhibiting AKT activity^97^ (and independently of AKT-mTOR activity^98^), and that loss of AKT in PTEN-null cancer cells induces autophagy,^99^ suggesting that tumor cells with reduced PTEN expression will have reduced autophagy in general. Indeed our data support this where the PTEN^Mut^ A2058 cells exhibit low basal autophagy as seen by low expression of the autophagy marker, LC3II, which does not alter markedly in response to WNT3A signaling (Supplementary Figure 7g). PTEN^WT^ cancer cells, however, are autophagy proficient, conferring a major mechanism of stress tolerance in these cells (such as during nutrient deprivation) for tumor cell survival, which makes them particularly susceptible to autophagy inhibition (such as that induced by WNT/β-catenin signaling).

Small-molecule WNT antagonists have been developed for cancer treatment, however, because the role of the WNT/β-catenin pathway is controversial, it is difficult to predict how such drugs could be implemented in the treatment of melanoma. Here we show that the effects of the WNT/β-catenin pathway differ vastly in melanoma depending on PTEN expression levels; when PTEN levels are high, β-catenin signaling reduces metastasis, inhibits mitophagy and reduces the bioenergetic status of the cells (none of this occurs in melanoma cells with depleted PTEN levels). Genomic subtyping in cancer is needed for the implementation of molecular therapies and recent TCGA stratification of cutaneous melanomas provides a potential guideline for therapeutic decisions.^100^ Our results suggest that PTEN expression level could represent a potential biomarker for the implementation of WNT inhibitors in melanoma.

## Materials and methods

### Reporter gene assay

Details of all cells, culture conditions, reagents and statistical analyses used are provided in the Supplementary Methods section of the Supplementary Material, along with western blot details and all additional methodology used to generate the data presented in the Supplementary Figures.

Melanoma cells were plated in 24-well plates and transiently transfected with 150 ng of either Super8TOPFlash or Super8FOPFlash reporter constructs^101^ for 6 h in media without serum or antibiotics. A Renilla luciferase expression plasmid was co-transfected for normalization of transfection efficiency. Transfected cells were then stimulated for 24 h with either rWNT3A (50 ng/ml) or carrier control. Cell extracts were assayed for luciferase activity using the dual luciferase kit (Promega UK, Southampton, UK), according to the manufacturer's instructions. Renilla-normalized TOPFlash results were then normalized to FOPFlash expression and the data presented as actual luminescence units.

### Apoptosis assay

Apo-TRACE Apoptotic Cell Staining Kit (Sigma-Aldrich, Dorset, UK) was used according to the manufacturer's instructions. Briefly, cells were serum starved for 48 h, washed in phosphate-buffered saline and Apo-TRACE added at 75 μg/ml for 1 h in the dark. Cell lysates were read at ex 328 nm, em 563 nm on a POLARstar Optima microplate reader (BMG Labtech, Aylesbury, UK). Results were normalized to total protein concentration, as assessed by BCA assay (Fisher Scientific UK Ltd, Loughborough, UK).

### *In vitro* wound assay

This was done as previously described.^26^ Briefly, a wound was scratched across a confluent monolayer of melanoma cells and the wells rinsed with fresh media to remove floating cells. Images were taken of the scratched area at × 10 magnification, where the wound-healing process was monitored at 24 and 48 h in the same location. Images were analyzed using Image J software (NIH, Bethesda, MD, USA) to measure the open area of cells and the data presented as fold-change in wound confluence over the control.

### Transwell assay

Melanoma cells were either stimulated in serum-free media or serum-starved for 48 h before the assay (as indicated for each experiment), resuspended in serum-free medium and added to the upper chambers of 8.0 μm pore inserts (Corning, Corning Optical Communications, Flintshire, UK) coated with Matrigel (BD Biosciences, Oxford, UK). Growth media were added to the lower chamber and the cells incubated for 48 h. Migrating cells were quantified after crystal violet staining. Each experiment was performed in triplicate. The insert was then removed and the number of invaded cells were analyzed as previously described.^36^

### Mouse xenograft experiments

Low passage, lentiviral transduced WNT3A overexpression or control cancer cells were suspended in phosphate-buffered saline at low passage. 10 × 10^5^/ml cells were injected into 11-week-old, male NOD *scid* gamma (NSG) mice through the tail vein using a 26G needle. Six mice were injected per group (as determined by G*Power3 analytical software^102^) and experiments performed in a blinded manner to ensure randomization of the approach. Lungs were harvested 4 weeks post injection and fixed with 10% neutral-buffered formalin (Fisher Scientific UK Ltd). All animal studies were performed using Institutional Animal Care and Use Committee protocols, as approved by a review board at the University of Washington.

### Measurements of OCR and ECAR

OCR and ECAR were measured using the XF^e^96 flux analyzer (Seahorse Bioscience, Agilent Technologies LDA UK Ltd, Stockport, UK) and the XF Cell Mito Stress Test kit (Seahorse Bioscience, Agilent Technologies LDA UK Ltd), according to the manufacturer's instructions. In all, 8x10^4^ cells were seeded onto poly-d-lysine-coated XF microplates. Briefly, metabolic flux measurements were assessed under basal conditions and in response to the ATP synthase inhibitor, oligomycin (2.5 μM), the electron transport chain (ETC) accelerator, FCCP (1 μM), and finally the ETC complex 1 and 3 inhibitors, antimycin A and rotenone (2.5 μM), respectively. Data were analyzed using the XF software (Seahorse Bioscience, Agilent Technologies LDA UK Ltd).

### CS activity assay

The CS Assay Kit (Sigma-Aldrich) was used according to the manufacturer's instructions. Cells were seeded and stimulated for 48 h before analysis and results normalized to total protein concentration for each sample.

### Lactate production assay

The Lactate Assay Kit (BioVision Inc., Milpitas, CA, USA) was used according to the manufacturer's instructions and experiments conducted as previously described.^26^ Melanoma cells were seeded and stimulated or not (as indicated for each experiment), 48 h before the analysis.

### Immunofluorescent and mitochondrial staining

Cells were grown and stimulated on 18-mm sterile coverslips for 48 h. MitoTracker stains (Invitrogen, Life Technologies Ltd; 250 nM) were diluted in phenol-red-free media and incubated with the cells for 15 min at 37 **°**C. The stain was then removed and replaced with 1 ml phenol-red-free media and incubated at 37 **°**C for 10 min. Following staining, cells were fixed using 5% paraformaldehyde for 10 min, permeabilized using 0.5% Triton X-100 for 5 min and then counterstained with Phalloidin 488 (0.25 units) and Hoechst 33342 (10 nM).

For immunostaining, MFN1, MFN2, OPA1 and LC3 antibodies were incubated with fixed/permeabilized cells before detection using fluorescent secondary antibodies (5 μg/ml), and counterstained with Hoechst 33342. After washing, cells were mounted with Vectashield mounting medium (Vector Laboratories Ltd, Peterborough, UK) and analyzed (as for the MitoTracker stained cells) by LSM 510 (Carl Zeiss Ltd, Cambridge, UK) to capture Z-stack images at optimum slice depths for each sample. Gain settings and exposure time were kept constant between samples. For Imaris (Bitplane AG) analysis, sampling was carried out using 3D images to calculate surface area staining by setting the background threshold to 0.5 μm. The area in defined size ranges was then calculated by Imaris software as a percentage of the total.

### Reverse transcription and quantitative PCR

The Human Mitochondrial to Nuclear DNA Ratio Kit (NovaQUANT, EMD Millipore, Watford, UK) was used according to the manufacturer's protocol for whole-cell lysis. In all, 50 cells per well in DNase/RNase-free water were used with a 2X Fast SYBR green mastermix. For reverse transcription–quantitative PCR, RNA was purified using the GeneJet RNA purification kit (Fisher Scientific UK Ltd.) following the manufacturer's protocol. In total, 2 μg of complementary DNA was synthesized using RevertAid M-MuLV Reverse Transcriptase (Fermentas, Fisher Scientific UK Ltd) and diluted to 100 ng/ml, followed by quantification using SYBR green reagent (Fermentas, Fisher Scientific UK Ltd) and a LightCycler 480 system (Roche, Welwyn Garden City, UK). *YWHAZ*, *UBC* and *ACTB* were used as reference genes for relative quantification of the transcripts under investigation. All oligos used for quantitative PCR are shown in Supplementary Table S5.

### Immunoprecipitation mass spectrometry

For sample preparation, non-glycoslyated β-catenin was removed using conA beads (BioWorld, Dublin, OH, USA). Samples were pre-cleared with rabbit IgG before incubation with β-catenin-coated Dynabeads G (Novex, Fisher Scientific UK Ltd). Immunoprecipitated proteins were solubilized in 1 m urea, 50 mM ammonium bicarbonate, pH 7.8, and heated to 50 °C for 20 min. Proteins were reduced with 2 mM DTT, alkylated with 15 mM iodoacetamide and digested overnight with a 1:50 ratio of trypsin to total protein. The resulting peptides were desalted on Waters Sep-Pak C18 cartridges (Waters, MA, USA). Peptides were measured by nano-LC-MS/MS on a Thermo Scientific Q Exactive (QE). Peptides were separated online by reverse-phase chromatography using heated 50 °C 30 cm C18 columns (75 mm ID packed with Magic C18 AQ 3 μM /100 A beads) in a 180 min gradient (1% to 45% acetonitrile with 0.1% formic acid) and separated at 250 nl/min. The QE was operated in data-dependent mode with the following settings: 70 000 resolution, 400–1600 m/z full scan, Top 10, and an 1.8 m/z isolation window. Identification and label-free quantification of peptides was done with MaxQuant 1.5 (Max Planck Institute of Biochemistry, Martinsried, Germany), using a 1% false discovery rate against the human Swiss-Prot/TrEMB (Swiss Institute of Bioinformatics, Geneva, Switzerland) database. Three replicates per condition were analyzed. Peptides were searched using a 5 p.p.m. mass error and a match between run window of 2 min. Proteins that were significantly regulated between conditions were identified using a permutation-based *t*-test (S1, false discovery rate 5%) in Perseus 1.4.1.3. Gene ontology (GO)-terms were analyzed using Protein ANalysis THrough Evolutionary Relationships (PANTHER) 9.0 (http://pantherdb.org).

### Membrane potential measurements

Mitochondrial membrane potential measurements were made using flow cytometry protocols. Cells were grown and stimulated for 48 h before incubation with JC-1 (eBioscience Ltd, Altrincham, UK) stain (2 μM) or TMRM (Fisher Scientific UK Ltd; 20 μM) for 2 h. All samples were diluted in phenol-red-free media. The stain was removed and replaced with 1 ml phenol-red-free media and incubated at 37 **°**C for 10 min before re-suspension in FACS buffer (phosphate-buffered saline+0.5% fetal calf serum) and analysis on the Accuri C6 flow cytometer (BD Biosciences).

## Supplementary Material

Supplementary Inforamtion

Supplementary Figure 8i

Supplementary Figure 8ii
