# Supplementary material for: WNT/β-catenin signaling regulates mitochondrial activity to alter the oncogenic potential of melanoma in a PTEN-dependent manner
Source: Oncogene. 2017 Jan 16;36(22):3119–36. doi: 10.1038/onc.2016.450 (PMC5467017; doi:10.1038/onc.2016.450)
Supplement: Supplementary Inforamtion [file onc2016450x1.docx]

WNT/β-catenin signaling regulates mitochondrial activity to alter the oncogenic potential of melanoma in a PTEN-dependent manner.

K. Brown *et al*.

**Supplementary Methods**

**Cell culture.** A2058, A375, M202, M229, Mel501 and SkMel28 melanoma cells were obtained from ATCC. Cells were maintained in RPMI (HyClone) containing 10% foetal bovine serum (FBS; Hyclone), 1% Penicillin–Streptomycin (Sigma) and 1% L-Glutamine (Sigma). To generate stable WNT3A over-expression cultures, the sequence for human *WNT3A* was amplified by PCR and cloned into third-generation Lentiviral vectors to generate WNT3A-iresGFP overexpression cells as previously described^15^. WNT3A overexpressing and control cells were sorted by FACS to obtain populations expressing equal levels of GFP. These stable, lentivirally-transduced, over-expression cells were cultured using the same conditions as described above for the other tumor lines, with the exception that 5% FBS was used. Recombinant human WNT3A (rWNT3A; R&D) and recombinant human DKK1 (rDKK1; R&D) were used at 50 ng/ml. Stimulation of cells with recombinant proteins or carrier controls were carried out in serum-free media for a period of 48 hours, unless otherwise stated. siRNA knockdown cells were reverse transfected with 20 nM siRNA in 6-well plates using RNAiMAX reagent, according to the manufacturer’s instructions (Invitrogen). Sequences used for siRNA oligos are shown in Supplementary Table S6. Cells were incubated for 72 hours following transfection before further analysis. siRNAs were obtained from Ambion (Applied Biosystems). Cells were never cultured beyond passage 25. All cells used were verified as mycoplasma-free and authenticated using SNP analysis.

**Antibodies and reagents.** All antibodies used were as follows (Western blot, WB; immunofluorescence, IF): Akt (Cell Signaling) 1:1000, phospho-Akt (pAKT, t308; Cell Signaling), Anti-Mouse IgG HRP (NEB) 1:10,000, Anti-mouse AF633 (Invitrogen) 5 µg/ml, Anti-Rabbit IgG HRP (NEB) 1:5000, Rabbit IgG Isotype control (Invitrogen) 1:1000, Actin (Santa Cruz) 1:2000, α-tubulin (Cell Signaling) 1:10,000, ß-catenin (Cell Signaling) 1:1000, Cytochrome c (AbD Serotec) 20 µg/ml, DRP1 (Abcam) 1:1000 for WB or 1:100 for IF, E-Cadherin (Cell Signaling) 1:1000, Hsp90 (Santa Cruz) 1:10,000, 1:1500, Ku80 (AbCam), Lamin A/C (Cell Signaling) 1:1000, LC3 (Sigma) 1:4000 for WB or 1:100 for IF, LDHA (Cell Signaling) 1:500, LDHB (Cell Signaling) 1:500, MFN1 (Abcam) 1:1000 for WB or 1:100 for IF, MFN2 (Abcam) 1:1000 for WB or 1:100 for IF, OPA1 (Abcam) 1:400 for WB or 1:100 for IF, P62 (Abcam) 1:1000, Parkin (Abcam) 1:500, PFKB (Cell Signaling) 1:500, PINK-1 (Novus) 1:200 or (Cell Signaling) 1:500, PTEN (Santa Cruz) 1:500, S-100 antibody (Invitrogen) 46.8 mg/mL, 1:1000 and WNT3A (R&D) 1:2000. LY294002 (Sigma), was used at 2 µM. Rapamycin (Sigma) was used at 200 nM. Details of recombinant proteins and siRNA oligos used are provided in the ‘cell culture’ section. All chemicals and reagents/kits used were of analytical grade. Unless otherwise stated, reagents were sourced from Sigma.

**Western blot.** Western analysis was conducted as previously described^1^. Briefly, cells were lysed using RIPA buffer (50 mM Tris–HCl, 10 mM MgCl_2_, 20% Glycerol and 1% Triton X-100) containing protease and phosphatase inhibitors (Roche). Protein concentration was determined by BCA assay (Pierce) and 10-30 μg used for Western blot analysis. Western blot samples were combined with NuPAGE LDS sample buffer (Life Technologies) containing 0.05% β-mercaptoethanol, incubated at 95°C for 5 minutes and then resolved using by SDS-PAGE (12% acrylamide). The resolved proteins were blotted onto nitrocellulose membrane and blocked with 5% milk or BSA (weight/volume). Primary antibodies were incubated at 4°C overnight, followed by incubation at room temperature with the respective horseradish peroxidase-conjugated secondary for 30 minutes. Details of the antibodies used are shown in the ‘antibodies and reagents’ section. Immunoreactive proteins were detected by enhanced chemiluminescence using a ChemiDoc XRS Molecular Imager (BioRad). Blots were quantified using the software available with this instrument. Uncropped images of the Western blots depicted in the main figures are shown in Supplementary Fig. 8. For all figures, representative blots are shown from replicate experiments. Mitochondrial proteins were extracted using a Mitochondrial Isolation Kit (ThermoScientific) according to the manufacturer’s instructions.

**Cell cycle analysis.** 20,000 ‘live events’ were analysed for cell cycle phases by flow cytometry. Following fixation in ice cold 70% ethanol at 4°C for 24-hours, cells were stained with propidium iodide/RNase A solution (Cell Signaling Technology) for 45 minutes at 37°C. Single cells were analysed for subG1, S and G2 peaks using an Accuri C6 flow cytometer (BD Biosciences). First gating on the live population in a fsc/ssc scatter was done and then (to exclude any doublets) in a fsc-a/fsc-h scatter. Analysis was performed with CFlow^®^ software (BD Biosciences).

**Cytochrome c release assay.** Apoptosis in melanoma cells was analysed by monitoring the release of Cytochrome c from the mitochondria, as previously described^2^. Briefly, cells were fixed using 5% paraformaldahyde, permeabilised using 0.5% Triton X-100 and incubated with an anti-cytochrome c antibody (AbD Serotec) at 20 µg/ml for an hour. An anti-mouse secondary antibody conjugated to AF633 (Invitrogen) was used and live events were measured on the C6 Accuri flow analyser in far-red channel. Fold-change in FLuorescent Units (FLU) was calculated.

**Three-dimensional cell culture.** Matrigel^TM^ (BD Biosciences) basement membrane matrix was coated at a volume of 100 µl/cm^2^ in a 48-well plate. 4x10^4^ cells previously stimulated with rWNT3A for 48-hours were seeded in 400 µl of growth media and left at 37°C for 48-hours before analysis. Cultures were analysed by phase contrast at 10X magnification using an inverted light microscope.

**Immunohistochemistry (IHC).** IHC was performed on paraffin-embedded sections from mouse tumour tissue. Briefly, sections were deparaffinized in Leica Bond Dewax Solution (Leica) and rehydrated through 100% ETOH. After, antigen retrieval was done using EDTA buffer pH 9.0 (Lieca Bond Epitope Retrieval Solution 2) at 100°C for 20 minutes. Samples were blocked for endogenous peroxidase activity with 3% H_2_O_2_ for 5 minutes and 10% Normal Goat Serum in TBS for 20 minutes before being incubated with Rabbit polyclonal S-100 antibody or Normal Rabbit IgG (isotype control, Invitrogen) for 30 minutes at room temperature. Sections were then incubated with Goat anti-Rabbit Poly-HRP polymer secondary detection (Leica) for 8 minutes at room temperature and Leica Bond Mixed Refine DAB substrate detection for 10 minutes at room temperature (Leica). After washing with H_2_O the sections were counter stained with Hematoxylin solution (Leica Bond Refine Kit) dehydrated through 100% ETOH, cleared in Xylene and mounted. Slides were scanned using a Nanozoomer Digital Pathology slide scanner (Olympus America). The digital images were then imported into Visiopharm software (Hoersholm) for analysis. Using the Visiomorph Digital Pathology module, regions of interest were applied around relevant areas using a tissue detect protocol and manual clean-up. The software was then programmed to label positively stained areas versus normal tissue areas, using project-specific configurations created for each staining set. Images were processed in batch using these configurations to generate the desired output calculations.

**Statistical analyses.** All data were obtained from at least three independent experiments. Results were plotted and analysed using GraphPad Prism software v.6.0 (GraphPad Software). Unless otherwise stated, data are presented as mean ± standard deviation (SD) and two group comparisons made using a two-tailed Student’s *t*-test. Statistical differences in S100 ratio staining in lung metastases from xenograft experiments were determined using Chi-squared correlation. Imaris data was analysed from the whole data set using a two-way ANOVA test. For TCGA data, 95% confidence intervals were plotted and a *z*-score for two-population proportions was calculated. For all tests, *p*-values <0.05 were taken as significant, as indicated in the figure legends.

**Supplementary Figure legends.**

**Figure 1s⏐Characterization of cell death in melanoma WNT3A overexpression cells. (a)** Flow cytometry analysis of the cell cycle in A375 and A2058 control and WNT3A overexpression cells. Live events were gated and cell doublets excluded. The % SubG1 count is statistically different in both lines in response to WNT3A over-expression, *p* = <0.05*. **(b)** Fold-change in cellular Cytochrome c measured by flow cytometry. A375 and A2058 control and WNT3A overexpression cells were fixed and stained with mouse anti-human cytochrome c antibody at 20 μg/ml. Mean ± SD. *p* = <0.05* or <0.01**.

**Figure 2s⏐PTEN^WT^ melanoma cells have reduced invasion, migration and metastasis in response to WNT/β-catenin signaling. (a)** Representative 10X images of wound closure after 24-hours in PTEN^WT^ and PTEN^Mut^ cells. 10X magnification images were taken at time 0 and after 24-hours of wound healing. Lines represent site of scratch in the monolayer. Scale bar, 100 μm. **(b)** Representative images of cell invasion after 48-hours in PTEN^WT^ and PTEN^Mut^ cells. Arrows highlight examples of crystal violet stained melanoma cells. Scale bar, 200 μm. **(c)** Example IHC images of S100 staining in lung tissue of NSG xenograft mice at 4X and 10X magnification. Metastatic lesions are circled by dash lines. 10X magnified regions are marked in the 4X magnification image as squares. Scale bar, 100μm. **(d)** Western blot analysis of PTEN expression levels in M229 and A2058 cells following transfection with PTEN or control vector for 72-hours. Loading control, α-tubulin. **(e)** Representative 10X images of wound closure after 24 hours in PTEN overexpression cells following treatment with rWNT3A (50 ng/ml) or carrier treatment. 10X magnification images were taken at time 0 and after 24 hours of wound healing. Lines represent site of scratch in the monolayer. Scale bar, 100μm. **(f)** Percentage of metastatic tumours in cutaneous melanoma patients (TCGA dataset) with low *AXIN2* expression (n=127).

**Figure 3s⏐XF^e^96 extracellular flux analysis of melanoma cells in response to WNT3A over-expression.** Seahorse XF^e^96 XF Cell Mito Stress Test Kit analysis of A375 and A2058 melanoma cells. **(a)** OCR of A375 control and WNT3A overexpression cells. **(b)** OCR of A2058 control and WNT3A overexpression cells. **(c)** ECAR of A375 control and WNT3A overexpression cells. **(d)** ECAR of A2058 control and WNT3A overexpression cells. For all panels from left to right, arrows represent addition of metabolic-modulating drugs; oligomycin (2.5 μM), FCCP (1 μM) and antimycin A (2.5 μM)+rotenone (2.5 μM). OCR, Oxygen Consumption Rate. ECAR, Extra Cellular Acidification Rate.

**Figure 4s⏐ WNT3A does not remodel mitochondrial architecture in PTEN^Mut^ melanoma cells. (a)** Representative images of individual PTEN^Mut^ melanoma cells stained with MitoTracker^®^ Deep Red (red), phalloidin (green) and Hoechst (blue). Scale bar, 50 μm. **(b)** Quantified Imaris data for PTEN^Mut^ melanoma cells. Numbers in each chart represents the percentage of mitochondria that range in size from 0.1-10 μM^2^ for each treatment.

**Figure 5s⏐ WNT3A-mediated metabolic and mitochondrial remodelling is dependent on active β-catenin signaling. (a)** RT-qPCR for *AXIN2* mRNA levels in A375 control and WNT3A overexpression cells following transfection with β-catenin siRNA or scrambled control siRNA for 72-hours. **(b)** rDKK1 (50 ng/ml) antagonizes WNT3A induction of β-catenin-TCF/LEF reporter activity in A375 overexpression cells. Results were normalized to the inverted promoter reporter plasmid (FOPFlash). **(c)** Label-free quantification of β-catenin-associated proteins is reproducible. Tryptic digestions of β-catenin or IgG immunoprecipitations (IP) were analyzed in triplicate by nano-LC MS/MS, with an average Pearson correlation of 0.93. For all panels, mean ± SD. *p* = <0.05*, <0.01** and <0.001***.

**Figure 6s⏐WNT3A does not increase mitochondrial numbers in PTEN^Mut^, but does increase ΔΨm in PTEN^WT^ melanoma cells. (a)** Total mitochondrial amount in A2058 overexpression cells was monitored by analysing mitochondrial DNA content normalised to nuclear DNA using PCR, to provide a relative copy number (RCN) ratio. Mean shown ± SD**.** **(b)** TMRM (20 μM) analysis of ΔΨm in A375 control and WNT3A overexpression cells by flow cytometry. *p* = <0.05*.

**Figure 7s⏐WNT3A increases mitochondrial fusion in a PTEN-dependent manner. (a)** Quantification of the protein expression of MFN1 in PTEN^WT^ melanoma cells stimulated with carrier control or rWNT3A (50 ng/ml) for 48-hours. Calculated from data presented in Fig. 7b. **(b)** IF images of MFN1, MFN2 and OPA1 expression (red) in A375 control and WNT3A overexpression cells. Images counterstained with Hoechst (blue). Scale bar 50 μm. **(c)** Flow cytometric analysis of mitochondrial fusion protein expression in A375 control and WNT3A overexpression cells, as indicated. **(d)** Western blot analysis of PTEN expression levels in A375 WNT3A overexpression cells following transfection with PTEN siRNA or a scrambled control siRNA for 72-hours. **(e)** Extracted mitochondria from A375 control and WNT3A overexpression cells were analysed by western blot for Parkin expression. Ponceau S staining served as a loading control. **(f)** Western blot analysis of PINK1 (Cell Signaling) in A375 cells following stimulation with carrier control or rWNT3A (50 ng/ml) for 48-hours and carrier control or FCCP (10 μM) for 2 hours. **(g)** A2058 control and WNT3A overexpression cells were analyzed by western blot for the autophagy marker LC3 (left panel). α-tubulin served as the loading control. Right panel, densitometry assessment of the ratio of LC3I to LC3II, based on the data shown in left panel. For all panels, mean ± SD. *p* = <0.05*, <0.01** and <0.001***.

**Figure 8s⏐Uncropped western blot analysis of figures used throughout manuscript.** Boxes indicate bands of interest. Arrows indicate protein name and molecular weight. **(a)** Fig. 1b. **(b)** Fig. 1e. **(c)** Fig. 2g. **(d)** Supplementary Fig. 2d. **(e)** Fig. 5a. **(f)** Fig. 5j. **(g)** Fig. 7a. **(h)** Fig. 7b. **(i)** Fig. 7c. **(j)** Fig. 7d. **(k)** Fig. 7g. **(l)** Fig. 7h. **(m)** Supplementary Fig. 7d. **(n)** Supplementary Fig. 7e. **(o)** Supplementary Fig. 7f. **(p)** Supplementary Fig. 7g.

**Supplementary Table S1⏐Summary of TCGA data for high AXIN2 mRNA expressing cutaneous melanomas^3^.** 63% of the highest *AXIN2* expressing samples were selected, as this is the percentage of tumors expected to have active β-catenin signaling^4^. Patient cohorts were classified as either PTEN low expressing (highlighted in yellow) or PTEN high expressing tumors, based on reverse phase protein lysate microarray analysis. Clinical information shown includes; vital status, Breslow thickness, Clark level, ulceration status, mitotic rate and American Joint Committee on Cancer (AJCC) staging system information. N/A: data not available/not applicable. All data was collected August 2016.

| **TCGA Patient identifier** | **Normalised Axin2** | **PTEN Fold Change** | **Age** | **Vital status** | **Breslow thickness** | **Clark level** | **Ulceration** | **Mitotic rate** | **AJCC stage** | **AJCC nodes** | **AJCC mets** |
| --- | --- | --- | --- | --- | --- | --- | --- | --- | --- | --- | --- |
| TCGA-GN-A262 | 105.9678 | 0.208074955 | 47 | Alive | 3 | N/A | N/A | N/A | N/A | N/A | N/A |
| TCGA-EB-A4IQ | 171.0983 | 0.254448668 | 42 | Dead | 15 | N/A | Yes | N/A | Stage IIIB | N1 | M0 |
| TCGA-EB-A5UM | 106.1489 | 0.288696805 | 48 | Alive | 5 | N/A | Yes | N/A | Stage IIC | N0 | M0 |
| TCGA-XV-A9VZ | 541.5901 | 0.297121293 | 48 | Alive | 5 | IV | N/A | N/A | Stage II | N0 | M0 |
| TCGA-DA-A95W | 132.6379 | 0.306411123 | 52 | Alive | 4.1 | IV | Yes | N/A | Stage IIIC | N1b | M0 |
| TCGA-EB-A6QZ | 125.8329 | 0.307113718 | 76 | Dead | 3 | N/A | No | N/A | Stage IIA | N0 | M0 |
| TCGA-RP-A693 | 87.8119 | 0.318349996 | 77 | Alive | N/A | N/A | N/A | N/A | Stage IV | N1 | M1c |
| TCGA-EB-A3XD | 657.5 | 0.318839125 | 53 | Alive | N/A | N/A | N/A | N/A | Stage IIIC | NX | M0 |
| TCGA-D9-A4Z2 | 223.4799 | 0.320014107 | 50 | Dead | 25 | IV | Yes | 10 | Stage IIIC | N3 | M0 |
| TCGA-EB-A5VU | 183.6 | 0.320048694 | 56 | Dead | 15 | N/A | Yes | N/A | Stage IIIB | N1 | M0 |
| TCGA-FW-A3I3 | 187.8335 | 0.323472754 | 59 | Alive | N/A | N/A | N/A | N/A | Stage IV | N0 | M1 |
| TCGA-BF-A3DM | 273.8629 | 0.328433748 | 63 | Alive | 1.5 | V | Yes | N/A | Stage IIA | N0 | M0 |
| TCGA-EB-A4IS | 95.6173 | 0.331272347 | 77 | Alive | 2.5 | N/A | Yes | N/A | Stage IIIB | N1 | M0 |
| TCGA-RP-A6K9 | 334.2015 | 0.344865379 | N/A | Alive | N/A | N/A | N/A | N/A | N/A | N/A | N/A |
| TCGA-BF-A3DN | 85.5382 | 0.355960162 | 81 | Alive | 3 | IV | Yes | N/A | Stage IIIC | N3 | M0 |
| TCGA-DA-A95V | 159.8408 | 0.37333161 | 83 | Alive | 4.1 | IV | Yes | N/A | Stage IIC | N0 | N/A |
| TCGA-EB-A3XF | 133.5777 | 0.374259811 | 57 | Alive | 10 | N/A | Yes | N/A | Stage IIC | N0 | M0 |
| TCGA-WE-AAA3 | 152.3627 | 0.400120404 | 84 | Alive | 7 | N/A | Yes | 12 | Stage IIIC | N2b | M0 |
| TCGA-EB-A3Y6 | 240.865 | 0.405609323 | 56 | Alive | 4.5 | N/A | Yes | N/A | Stage IIC | N0 | M0 |
| TCGA-EE-A2MI | 208.1252 | 0.405808598 | 43 | Dead | 0.4 | II | N/A | N/A | Stage IIB | N0 | M0 |
| TCGA-ER-A42K | 108.2045 | 0.40622691 | 40 | Dead | 5.5 | IV | Yes | 18.3 | Stage IIIC | N3 | M0 |
| TCGA-D9-A1JX | 270.4467 | 0.407273228 | 80 | Dead | N/A | III | No | N/A | N/A | NX | M0 |
| TCGA-D3-A8GE | 180.3028 | 0.414747288 | 62 | Alive | 10 | N/A | Yes | N/A | Stage IIC | N0 | M0 |
| TCGA-EB-A44N | 256.8593 | 0.424877816 | 59 | Dead | 5 | III | Yes | N/A | Stage IIC | N0 | M0 |
| TCGA-EE-A3JE | 98.5663 | 0.428366572 | 75 | Alive | 3.7 | IV | Yes | 6 | Stage IIIB | N1a | M0 |
| TCGA-WE-A8ZT | 260.858 | 0.438174999 | 25 | Alive | 4 | N/A | Yes | 14 | Stage IV | N1b | M1b |
| TCGA-EB-A85I | 190.3922 | 0.445981592 | 66 | Alive | 10 | N/A | Yes | N/A | Stage IIC | N0 | M0 |
| TCGA-XV-AAZW | 186.456 | 0.454016488 | 62 | Dead | 13 | V | N/A | N/A | Stage II | N0 | M0 |
| TCGA-D3-A3MO | 191.5696 | 0.4665669 | 47 | Dead | N/A | N/A | N/A | N/A | Stage III | N2c | M0 |
| TCGA-EB-A4OY | 738.3881 | 0.474585921 | 52 | Alive | 0.9 | III | No | 4 | Stage IA | N0 | M0 |
| TCGA-BF-AAP6 | 176.7324 | 0.477365948 | 55 | Alive | 20 | IV | Yes | N/A | Stage III | N2 | M0 |
| TCGA-ER-A2NC | 279.1379 | 0.479094183 | 50 | Dead | 1.97 | N/A | No | N/A | Stage IB | N0 | M0 |
| TCGA-D3-A8GV | 355.233 | 0.486661041 | 25 | Dead | N/A | N/A | N/A | N/A | Stage IIB | N0 | M0 |
| TCGA-WE-A8ZQ | 97.7113 | 0.495398866 | 48 | Alive | 2.3 | N/A | No | 6 | Stage IIA | N0 | M0 |
| TCGA-EE-A2A0 | 106.7312 | 0.500793177 | 77 | Dead | 2.2 | IV | No | 2 | Stage IIA | N0 | M0 |
| TCGA-D9-A6E9 | 192.0981 | 0.501267064 | 75 | Alive | 4 | IV | No | N/A | Stage IIIA | N1 | M0 |
| TCGA-BF-A5EP | 234.2362 | 0.514857301 | 75 | Alive | 70 | V | Yes | N/A | Stage IIIC | N3 | M0 |
| TCGA-BF-AAOU | 95.5208 | 0.529906711 | 73 | Alive | 12 | IV | Yes | N/A | Stage IIC | N0 | M0 |
| TCGA-D3-A1Q4 | 100.593 | 0.535359091 | 53 | Alive | 1.45 | IV | Yes | 0 | Stage IIIC | N1b | M0 |
| TCGA-D3-A2JC | 179.2491 | 0.548183821 | 53 | Dead | 3.2 | IV | Yes | 13 | Stage IIB | N0 | M0 |
| TCGA-DA-A3F8 | 1257.4639 | 0.556453009 | 39 | Alive | 1.9 | IV | No | N/A | Stage IIIB | N2b | M0 |
| TCGA-BF-A5EO | 197.6264 | 0.560704817 | 65 | Alive | 8 | III | Yes | N/A | Stage IIC | N0 | M0 |
| TCGA-D3-A3MR | 97.64 | 0.580926884 | 42 | Alive | N/A | N/A | N/A | N/A | Stage III | N1b | M0 |
| TCGA-D3-A1Q1 | 217.4391 | 0.585262771 | 79 | Dead | 0.4 | II | No | 3 | Stage IIIC | N3 | M0 |
| TCGA-D3-A51K | 88.785 | 0.591963978 | 49 | Alive | 10 | N/A | Yes | N/A | Stage IIIC | N1b | M0 |
| TCGA-EB-A3Y7 | 595.9291 | 0.601143427 | 86 | Dead | 4 | N/A | No | N/A | Stage IIIB | N2c | M0 |
| TCGA-GN-A268 | 878.3769 | 0.607200188 | 83 | Dead | 9.8 | V | No | 1.67 | Stage IIIB | N1a | M0 |
| TCGA-ER-A2NE | 113.5345 | 0.610881996 | 39 | Dead | N/A | N/A | N/A | N/A | Stage 0 | N0 | M0 |
| TCGA-EB-A3HV | 140.4714 | 0.622702016 | 37 | Alive | 13 | IV | Yes | N/A | Stage IIC | N0 | M0 |
| TCGA-XV-A9W5 | 150.1068 | 0.624470255 | 51 | Alive | 2 | IV | N/A | N/A | Stage IB | N0 | M0 |
| TCGA-WE-A8K5 | 152.6807 | 0.639131885 | 65 | Dead | 1.7 | III | No | 12 | Stage IV | N3 | M1c |
| TCGA-D9-A4Z6 | 280.2198 | 0.642177128 | 54 | Dead | N/A | N/A | N/A | N/A | Stage IIIC | N1b | M0 |
| TCGA-GN-A4U9 | 125.5887 | 0.655600411 | 71 | Dead | 1.7 | IV | Yes | 5 | Stage IIIC | N3 | M0 |
| TCGA-EB-A97M | 223.6959 | 0.663523695 | 66 | Alive | 15 | IV | Yes | N/A | Stage IIC | N0 | M0 |
| TCGA-OD-A75X | 143.4412 | 0.671782186 | 49 | Dead | N/A | N/A | N/A | N/A | N/A | NX | M1 |
| TCGA-EB-A6R0 | 101.1162 | 0.688275746 | 58 | Dead | 10 | IV | Yes | N/A | Stage IIC | N0 | M0 |
| TCGA-BF-A1PU | 130.1948 | 0.698254571 | 46 | Alive | 13 | III | Yes | N/A | Stage IIC | N0 | M0 |
| TCGA-WE-A8JZ | 113.7081 | 0.699000776 | 70 | Alive | 14 | N/A | Yes | 9 | Stage IIIB | N/A | M0 |
| TCGA-D9-A4Z3 | 261.6036 | 0.707939817 | 73 | Alive | 7.5 | V | Yes | 10 | Stage IIIC | N1b | M0 |
| TCGA-HR-A5NC | 85.9268 | 0.722103656 | 90 | Alive | 8 | V | N/A | N/A | N/A | NX | M0 |
| TCGA-EE-A2GS | 83.3828 | 0.724046255 | 28 | Dead | 1.8 | IV | No | 6 | Stage IB | N0 | M0 |
| TCGA-D3-A8GN | 263.7302 | 0.727356259 | 27 | Alive | N/A | N/A | N/A | N/A | Stage IB | N0 | M0 |
| TCGA-EB-A24C | 169.9458 | 0.728543039 | 56 | Alive | 10 | N/A | Yes | N/A | N/A | NX | M0 |
| TCGA-D9-A6EC | 194.6202 | 0.72939096 | 56 | Alive | 3 | III | No | N/A | Stage IIIA | N1 | M0 |
| TCGA-DA-A960 | 896.6837 | 0.743519334 | 73 | Alive | 2.32 | IV | Yes | N/A | Stage IIB | N0 | M0 |
| TCGA-FS-A1ZN | 169.0821 | 0.748600545 | 43 | Dead | 8 | IV | Yes | N/A | Stage IIIA | N1a | M0 |
| TCGA-FS-A1Z0 | 100.6849 | 0.750812667 | 32 | Dead | 0.95 | III | No | N/A | Stage IA | N0 | M0 |
| TCGA-EE-A3JH | 221.3824 | 0.75181588 | 54 | Alive | 1.3 | III | N/A | 2 | Stage IB | N0 | M0 |
| TCGA-EE-A2MS | 612.4003 | 0.776240651 | 72 | Alive | 1.8 | IV | No | 4 | Stage II | N0 | M0 |
| TCGA-EB-A44P | 216.6159 | 0.784489374 | 58 | Alive | N/A | N/A | No | N/A | Stage IIC | N0 | M0 |
| TCGA-GN-A8LK | 1172.3155 | 0.787395161 | 70 | Dead | 0.7 | N/A | No | 0 | Stage IB | NX | N/A |
| TCGA-DA-A1I8 | 84.4524 | 0.787518479 | 63 | Dead | 8 | IV | Yes | N/A | Stage IIC | N0 | M0 |
| TCGA-EB-A44R | 248.8133 | 0.791476834 | 52 | Dead | N/A | N/A | N/A | N/A | Stage IIIB | N2b | M0 |
| TCGA-ER-A19O | 939.9113 | 0.794949327 | 56 | Dead | N/A | N/A | N/A | N/A | Stage IIIB | N1b | M0 |
| TCGA-EE-A17X | 604.1364 | 0.802703648 | 54 | Dead | 0.8 | III | No | 6 | Stage IA | N0 | M0 |
| TCGA-ER-A1A1 | 298.1262 | 0.819457486 | 58 | Alive | N/A | N/A | N/A | N/A | Stage IIIC | N3 | M0 |
| TCGA-EB-A5SF | 96.5595 | 0.824777152 | 78 | Dead | 11 | V | Yes | N/A | Stage IIC | NX | M0 |
| TCGA-WE-AA9Y | 203.372 | 0.843229 | 37 | Alive | 1.4 | N/A | No | 4 | Stage IIIC | N3 | M0 |
| TCGA-EE-A2GC | 258.9606 | 0.847596009 | 82 | Alive | 2.3 | III | Yes | 35 | Stage IIB | N0 | M0 |
| TCGA-FS-A1ZK | 116.4216 | 0.852671478 | 68 | Dead | 5 | IV | N/A | N/A | Stage II | N0 | M0 |
| TCGA-EE-A20F | 120.6798 | 0.853860738 | 53 | Alive | 0.5 | N/A | N/A | N/A | Stage I | N0 | M0 |
| TCGA-EB-A299 | 311.3807 | 0.859617039 | 63 | Alive | 2 | III | Yes | 4 | Stage IIA | N0 | M0 |
| TCGA-EE-A2GH | 86.0816 | 0.864338683 | 34 | Alive | 0.8 | III | N/A | 2 | Stage I | N0 | M0 |
| TCGA-EB-A85J | 193.0711 | 0.869604179 | 66 | Alive | 5.5 | N/A | Yes | N/A | Stage IIB | N0 | M0 |
| TCGA-BF-A3DL | 92.6244 | 0.871524246 | 84 | Alive | 3 | IV | Yes | N/A | Stage IIIB | N2 | M0 |
| TCGA-D3-A3MV | 124.529 | 0.876093758 | 38 | Alive | 1.2 | IV | Yes | 5 | Stage IIIB | NX | M0 |
| TCGA-GN-A4U8 | 83.7828 | 0.876921031 | 51 | Alive | N/A | N/A | N/A | N/A | N/A | N/A | N/A |
| TCGA-D3-A1Q5 | 467.2416 | 0.878314307 | 60 | Dead | N/A | N/A | N/A | N/A | Stage IB | NX | M0 |
| TCGA-D3-A1Q7 | 94.9005 | 0.879959273 | 42 | Alive | 0.68 | III | No | 1 | Stage IB | N0 | M0 |
| TCGA-ER-A2NH | 144.1489 | 0.886589709 | 49 | Alive | 4 | IV | No | 1 | Stage IIIC | N3 | M0 |
| TCGA-EB-A41B | 237.8846 | 0.892558802 | 76 | Alive | 28 | V | Yes | N/A | Stage IIC | N0 | M0 |
| TCGA-EE-A29W | 149.2806 | 0.922327609 | 42 | Alive | 0 | I | N/A | N/A | Stage 0 | N0 | M0 |
| TCGA-BF-AAP4 | 396.3964 | 0.928864645 | 61 | Alive | 15 | V | Yes | N/A | Stage IIC | N0 | M0 |
| TCGA-EE-A2M8 | 107.1529 | 0.950474619 | 54 | Dead | 2 | IV | No | 4 | Stage III | N0 | M0 |
| TCGA-BF-AAP0 | 128.3186 | 0.954549787 | 40 | Alive | N/A | N/A | N/A | N/A | Stage IV | NX | M1 |
| TCGA-D9-A3Z4 | 84.1699 | 0.95829252 | 54 | Dead | 12 | V | Yes | N/A | Stage IIIC | N3 | M0 |
| TCGA-FR-A8YD | 108.6828 | 0.969887631 | 56 | Dead | 4.5 | IV | Yes | 5 | Stage IIC | N0 | M0 |
| TCGA-YG-AA3O | 123.3629 | 0.971001294 | 62 | Dead | N/A | N/A | N/A | N/A | N/A | N/A | N/A |
| TCGA-ER-A19A | 183.5447 | 0.977961274 | 79 | Alive | N/A | N/A | N/A | N/A | Stage IV | N0 | M1 |
| TCGA-ER-A19T | 105.1221 | 0.991471918 | 51 | Dead | 15 | V | No | 16 | Stage IV | N3 | M1a |
| TCGA-WE-A8K1 | 192.5863 | 1.005595697 | 74 | Alive | 3 | N/A | Yes | 5 | Stage IIIC | N3 | M0 |
| TCGA-EE-A2GP | 83.1057 | 1.006277242 | 80 | Dead | 4.2 | IV | Yes | 30 | Stage IIIB | N1a | M0 |
| TCGA-EB-A3XB | 202.4874 | 1.009595193 | 63 | Alive | N/A | IV | N/A | N/A | Stage II | N0 | M0 |
| TCGA-EB-A4OZ | 160.0704 | 1.013733709 | 41 | Alive | 18 | V | No | N/A | Stage IIIC | N3 | M0 |
| TCGA-ER-A2NG | 148.8061 | 1.014968495 | 43 | Dead | 3 | III | Yes | 9 | Stage IIIC | N3 | M0 |
| TCGA-EE-A29T | 152.2947 | 1.015448739 | 51 | Alive | N/A | N/A | N/A | N/A | N/A | NX | M0 |
| TCGA-D3-A5GN | 82.1142 | 1.017373761 | 15 | Alive | 0.6 | III | N/A | N/A | Stage I | N0 | M0 |
| TCGA-BF-A1PX | 172.0542 | 1.019022476 | 56 | Dead | 12 | III | Yes | 10 | Stage IIIB | N2a | M0 |
| TCGA-ER-A19W | 196.2262 | 1.019491664 | 48 | Dead | 0.75 | II | No | N/A | Stage I | N/A | N/A |
| TCGA-ER-A193 | 234.4945 | 1.023321274 | 62 | Dead | N/A | N/A | No | N/A | Stage IIB | N0 | M0 |
| TCGA-Z2-AA3S | 263.2299 | 1.024728884 | 58 | Alive | 0.5 | II | No | N/A | Stage IA | N0 | M0 |
| TCGA-EE-A2GT | 691.9339 | 1.048369089 | 77 | Alive | 2.2 | IV | No | 5 | Stage IIA | N0 | M0 |
| TCGA-FS-A4F9 | 556.5151 | 1.05133659 | 80 | Alive | 10 | N/A | Yes | N/A | Stage IIIC | N3 | M0 |
| TCGA-D3-A2JN | 96.5104 | 1.059310861 | 46 | Dead | N/A | N/A | N/A | N/A | Stage III | N1b | M0 |
| TCGA-EB-A5VV | 278.6622 | 1.05941751 | 74 | Alive | 4 | N/A | Yes | N/A | Stage IIIB | N1 | M0 |
| TCGA-Z2-AA3V | 222.3312 | 1.060741822 | 57 | Alive | 0.5 | N/A | No | 0.9 | Stage IA | N0 | M0 |
| TCGA-ER-A19H | 113.316 | 1.063240939 | 40 | Dead | 0.4 | II | N/A | N/A | Stage II | N0 | M0 |
| TCGA-EB-A42Z | 92.3474 | 1.073342934 | 58 | Alive | 6 | IV | Yes | N/A | Stage IIC | N0 | M0 |
| TCGA-EE-A184 | 138.6916 | 1.082269644 | 72 | Dead | 1.95 | IV | No | 2 | Stage IB | N0 | M0 |
| TCGA-EE-A182 | 290.3075 | 1.088834577 | 84 | Dead | 14 | V | Yes | 5 | Stage IIIC | N1b | M0 |
| TCGA-WE-A8ZY | 451.8545 | 1.089930953 | 62 | Dead | 3 | N/A | No | 3 | Stage IIA | N0 | M0 |
| TCGA-GN-A9SD | 93.7627 | 1.096814653 | 59 | Dead | 0.5 | III | No | 1 | Stage IA | N0 | M0 |
| TCGA-EB-A44Q | 862.8883 | 1.104545837 | 51 | Alive | N/A | N/A | Yes | N/A | Stage IIIC | N3 | M0 |
| TCGA-EB-A82B | 103.0242 | 1.104745078 | 58 | Alive | 20 | V | Yes | N/A | Stage III | N2 | M0 |
| TCGA-D3-A51T | 101.7544 | 1.107016654 | 59 | Alive | 4.2 | IV | Yes | 16 | Stage IIIC | N1b | M0 |
| TCGA-XV-A9W2 | 125.0033 | 1.114202727 | 81 | Alive | 1 | II | N/A | N/A | Stage I | N0 | M0 |
| TCGA-EB-A41A | 487.7492 | 1.128805232 | 90 | Alive | 7 | N/A | Yes | N/A | Stage IIC | N0 | M0 |
| TCGA-EB-A430 | 428.6656 | 1.131799517 | 83 | Alive | 18 | IV | Yes | N/A | Stage IIC | N0 | M0 |
| TCGA-EE-A29V | 115.913 | 1.156614738 | 85 | Dead | 3.5 | IV | Yes | 14 | Stage IIIC | N1b | M0 |
| TCGA-EB-A57M | 188.6054 | 1.1567811 | 56 | Dead | 8 | N/A | Yes | N/A | Stage IIIB | N1 | M0 |
| TCGA-ER-A42L | 182.5319 | 1.160626741 | 49 | Alive | 1.46 | IV | No | 1 | Stage II | N0 | M0 |
| TCGA-EB-A550 | 283.0649 | 1.163641372 | 75 | Dead | 15 | IV | Yes | N/A | Stage IIC | N0 | M0 |
| TCGA-GF-A2C7 | 122.1133 | 1.169433908 | 48 | Alive | N/A | IV | Yes | 2 | Stage IIC | N0 | M0 |
| TCGA-ER-A199 | 320.519 | 1.182919197 | 86 | Dead | 3 | IV | Yes | 8 | Stage IIIC | N3 | M0 |
| TCGA-FR-A729 | 95.494 | 1.183034708 | 38 | Alive | 0.25 | II | No | N/A | Stage I | N0 | M0 |
| TCGA-XV-AB01 | 384.2975 | 1.185871266 | 54 | Alive | N/A | N/A | N/A | N/A | Stage II | N0 | M0 |
| TCGA-D3-A8GS | 554.864 | 1.187164769 | 52 | Dead | 0.6 | III | N/A | N/A | Stage I | N0 | M0 |
| TCGA-D3-A2J6 | 106.503 | 1.18973458 | 65 | Dead | 3.2 | IV | Yes | 13 | Stage IIB | N0 | M0 |
| TCGA-FS-A1ZG | 1691.4137 | 1.190794207 | 60 | Dead | 6 | V | Yes | N/A | Stage IIIC | N2b | M0 |
| TCGA-D3-A8GM | 271.5652 | 1.191261067 | 73 | Dead | 3.8 | IV | Yes | 9 | Stage IIB | N0 | M0 |
| TCGA-FR-A7U9 | 152.5012 | 1.196866756 | 63 | Alive | 2.8 | IV | Yes | 11 | Stage IIC | N0 | M0 |
| TCGA-D3-A1Q8 | 107.1913 | 1.196900132 | 33 | Dead | N/A | N/A | N/A | N/A | Stage IV | N3 | M1b |
| TCGA-3N-A9WC | 139.7691 | 1.198941415 | 82 | Alive | 1.8 | N/A | Yes | 4 | Stage IIA | N0 | M0 |
| TCGA-ER-A19D | 176.1691 | 1.201871758 | 46 | Dead | 1.75 | IV | No | N/A | Stage IB | N0 | M0 |
| TCGA-EE-A29G | 286.1443 | 1.203256232 | 53 | Dead | 7.4 | V | No | 14 | Stage IIIA | N2a | M0 |
| TCGA-D3-A51F | 92.7331 | 1.205658887 | 51 | Alive | 7 | IV | Yes | 11 | Stage IIC | N0 | M0 |
| TCGA-EB-A5SH | 881.8369 | 1.208050883 | 60 | Alive | 5 | V | Yes | N/A | Stage III | N0 | M0 |
| TCGA-D3-A3C1 | 111.2903 | 1.211253257 | N/A | Alive | N/A | N/A | N/A | N/A | Stage I | N0 | M0 |
| TCGA-EB-A5UN | 209.7561 | 1.216792555 | 49 | Alive | 14 | IV | Yes | 4 | Stage IIC | N0 | M0 |
| TCGA-HR-A2OG | 135.2025 | 1.220038093 | 50 | Alive | N/A | N/A | N/A | N/A | Stage I | N0 | M0 |
| TCGA-WE-A8ZR | 147.4718 | 1.229430332 | 49 | Dead | 7.3 | N/A | Yes | 18 | Stage IIIC | N1b | M0 |
| TCGA-EE-A2A6 | 235.02 | 1.236560305 | 43 | Alive | 0.55 | III | No | 0 | Stage IA | N0 | M0 |
| TCGA-FR-A8YE | 256.1031 | 1.242381524 | 41 | Alive | 0.79 | III | No | N/A | Stage IA | N0 | M0 |
| TCGA-DA-A1HW | 167.4734 | 1.256178251 | 37 | Alive | 0.7 | III | No | N/A | Stage IIIB | N1b | M0 |
| TCGA-LH-A9QB | 115.8702 | 1.260804539 | 24 | Alive | N/A | III | N/A | N/A | Stage II | N0 | M0 |
| TCGA-EB-A3XE | 127.1008 | 1.261955359 | 77 | Alive | 3 | N/A | No | N/A | Stage IIA | N0 | M0 |
| TCGA-FW-A5DY | 112.8512 | 1.268051583 | 48 | Alive | N/A | N/A | N/A | N/A | Stage III | N/A | M0 |
| TCGA-ER-A19P | 87.0257 | 1.269122598 | 47 | Dead | N/A | IV | N/A | N/A | N/A | N0 | M0 |
| TCGA-EE-A29B | 553.7262 | 1.273541176 | 67 | Dead | 4 | III | Yes | N/A | Stage IIB | N0 | M0 |
| TCGA-FR-A3YN | 227.2331 | 1.275533874 | 44 | Alive | 1.25 | IV | No | N/A | Stage IB | N0 | M0 |
| TCGA-ER-A197 | 157.9871 | 1.276051894 | 83 | Dead | 8 | N/A | Yes | N/A | Stage IIIB | N1a | M0 |
| TCGA-EE-A3JD | 145.3055 | 1.276220453 | 70 | Dead | N/A | N/A | N/A | N/A | Stage III | N1b | M0 |
| TCGA-FS-A1ZJ | 143.794 | 1.278517164 | 75 | Dead | 1.22 | IV | No | N/A | Stage I | N0 | M0 |
| TCGA-BF-AAOX | 1656.1135 | 1.282716823 | 83 | Alive | II | IV | Yes | N/A | Stage IIC | N0 | M0 |
| TCGA-EE-A29M | 628.0177 | 1.287067222 | 33 | Alive | 1.02 | III | No | 3 | Stage IB | N0 | M0 |
| TCGA-BF-AAP7 | 97.8848 | 1.289669197 | 76 | Alive | 5 | IV | Yes | N/A | Stage IIC | N0 | M0 |
| TCGA-D9-A148 | 314.8613 | 1.29114553 | 40 | Alive | N/A | N/A | No | N/A | Stage IV | NX | M1b |
| TCGA-DA-A1IA | 169.0327 | 1.29345279 | 32 | Dead | 1.3 | IV | No | N/A | Stage IIIB | N1b | M0 |
| TCGA-D9-A149 | 208.3886 | 1.294084398 | 65 | Alive | N/A | N/A | No | N/A | N/A | NX | M0 |
| TCGA-EB-A82C | 129.5314 | 1.309010398 | 70 | Alive | 10 | V | Yes | N/A | Stage IIC | N0 | M0 |
| TCGA-EE-A2GK | 532.552 | 1.324171732 | 46 | Alive | 0.8 | III | N/A | N/A | Stage I | N0 | M0 |
| TCGA-DA-A1IC | 273.566 | 1.32662062 | 81 | Dead | 2.5 | IV | No | N/A | Stage IIIB | N2c | M0 |
| TCGA-FS-A1YW | 342.6504 | 1.328927504 | 52 | Dead | 1 | IV | No | N/A | Stage IB | N0 | M0 |
| TCGA-DA-A1IB | 140.246 | 1.337055925 | 69 | Alive | 1.5 | IV | Yes | N/A | Stage IIIC | N2b | M0 |
| TCGA-EE-A29N | 169.6588 | 1.357758706 | 78 | Dead | N/A | N/A | N/A | N/A | Stage I | N0 | M0 |
| TCGA-FS-A1ZH | 170.287 | 1.36868413 | 71 | Dead | 3 | IV | Yes | N/A | Stage IV | N2c | M1c |
| TCGA-FS-A1ZQ | 166.508 | 1.372125828 | 31 | Dead | N/A | N/A | N/A | N/A | Stage I | N0 | M0 |
| TCGA-EB-A5KH | 138.5653 | 1.380922099 | 55 | Dead | N/A | N/A | N/A | N/A | Stage II | N0 | M0 |
| TCGA-EE-A3AE | 708.9796 | 1.385424534 | 52 | Alive | 0.9 | III | No | 4 | Stage IA | N0 | M0 |
| TCGA-D3-A1QA | 117.9104 | 1.40371879 | 55 | Alive | 1.12 | III | No | 2 | Stage IB | N0 | M0 |
| TCGA-ER-A19B | 217.2261 | 1.444256603 | 42 | Dead | N/A | N/A | N/A | N/A | N/A | N0 | M0 |
| TCGA-EE-A2MR | 186.3636 | 1.44650159 | 61 | Alive | 1.25 | III | No | N/A | Stage I | N0 | M0 |
| TCGA-EB-A6L9 | 156.3417 | 1.448278761 | 55 | Alive | N/A | N/A | Yes | N/A | Stage IIIC | NX | M0 |
| TCGA-BF-A5EQ | 266.3168 | 1.464571133 | 63 | Alive | 5 | III | N/A | N/A | Stage IIC | N0 | M0 |
| TCGA-WE-A8K4 | 195.9615 | 1.51541651 | 85 | Alive | 12 | N/A | N/A | 4 | Stage IIB | N0 | M0 |
| TCGA-EB-A42Y | 7743.2594 | 1.515934047 | 46 | Alive | 13 | III | Yes | N/A | Stage IIC | N0 | M0 |
| TCGA-GN-A26D | 123.6164 | 1.575462609 | 72 | Dead | 12 | V | Yes | 5 | Stage IIC | N0 | N/A |
| TCGA-ER-A198 | 520.4168 | 1.589715684 | 45 | Dead | 10 | N/A | Yes | N/A | N/A | NX | M0 |
| TCGA-GN-A8LL | 307.2755 | 1.594995866 | 68 | Dead | 5 | N/A | Yes | N/A | Stage IIC | N0 | M0 |
| TCGA-ER-A2NF | 112.069 | 1.597817713 | 53 | Dead | 4 | V | Yes | N/A | Stage IIIB | N3 | M0 |
| TCGA-ER-A195 | 9272.1223 | 1.605245842 | 46 | Dead | N/A | N/A | N/A | N/A | N/A | N0 | M0 |
| TCGA-EB-A4XL | 241.9945 | 1.617780262 | 56 | Alive | 7 | IV | Yes | N/A | Stage IIC | N0 | M0 |
| TCGA-FS-A4FC | 127.176 | 1.691165752 | 75 | Dead | 4 | IV | No | N/A | Stage IIA | N0 | M0 |
| TCGA-YD-A9TB | 113.3582 | 1.695126559 | N/A | Alive | 1.5 | IV | No | 3 | Stage 0 | N0 | M0 |
| TCGA-BF-A3DJ | 98.9144 | 1.73231266 | 36 | Alive | 11 | III | Yes | N/A | Stage IIIB | N1 | M0 |
| TCGA-D9-A4Z5 | 631.2278 | 1.732544693 | 68 | Alive | N/A | III | No | N/A | Stage IIB | N0 | M0 |
| TCGA-EE-A180 | 152.4242 | 1.770963885 | 51 | Alive | N/A | N/A | N/A | N/A | N/A | N0 | M0 |
| TCGA-EE-A2A2 | 143.8528 | 1.776146021 | 71 | Alive | 7 | IV | Yes | 8 | Stage IIIC | N1b | M0 |
| TCGA-EE-A2MJ | 83.9852 | 1.802986806 | 60 | Dead | 11 | IV | No | 8 | Stage II | N0 | M0 |
| TCGA-FS-A1ZP | 88.0084 | 1.804525972 | 52 | Dead | 2.8 | III | N/A | N/A | Stage II | N0 | M0 |
| TCGA-ER-A19K | 93.6546 | 1.837267161 | 79 | Dead | 6.8 | V | Yes | N/A | Stage IIC | N0 | M0 |
| TCGA-D9-A1X3 | 148.8476 | 1.862717708 | 63 | Alive | 12 | IV | Yes | N/A | N/A | NX | N/A |
| TCGA-FR-A726 | 223.7874 | 1.889283653 | 90 | Dead | 14 | IV | Yes | 26 | Stage IIC | N0 | M0 |
| TCGA-EE-A2MN | 104.3838 | 2.026029964 | 58 | Dead | 1 | III | No | 0 | Stage I | N0 | M0 |
| TCGA-YG-AA3P | 258.391 | 2.043227321 | 63 | Alive | 10 | V | No | 6 | Stage IIB | N0 | M0 |
| TCGA-DA-A1I2 | 190.9771 | 2.05237416 | 45 | Dead | N/A | IV | Yes | N/A | Stage III | N2b | M0 |
| TCGA-EE-A2GL | 120.3994 | 2.582797572 | 40 | Alive | 3.1 | IV | No | 3 | Stage IIA | N0 | M0 |
| TCGA-EE-A181 | 102.0202 | 2.607201881 | 82 | Dead | 2.5 | IV | N/A | 4 | Stage II | N0 | M0 |
| TCGA-EE-A20C | 176.1589 | 2.999856943 | 59 | Dead | N/A | I | N/A | N/A | Stage 0 | N0 | M0 |

**Supplementary Table S2⏐Summary of TCGA data for low AXIN2 mRNA expressing cutaneous melanomas^3^.** 47% of the lowest *AXIN2* expressing samples were selected, as this is the percentage of tumors expected to have low β-catenin signaling^4^. Patient cohorts were classified as either PTEN low expressing (highlighted in yellow) or PTEN high expressing tumors, based on reverse phase protein lysate microarray analysis. Clinical information shown includes; vital status, Breslow thickness, Clark level, ulceration status, mitotic rate and AJCC staging system information. N/A: data not available/not applicable. All data was collected August 2016.

| **Patient identifier** | **Normalised Axin2** | **PTEN Fold Change** | **Age** | **Vital status** | **Breslow thickness** | **Clark level** | **Ulceration** | **Mitotic rate** | **AJCC stage** | **AJCC nodes** | **AJCC mets** |
| --- | --- | --- | --- | --- | --- | --- | --- | --- | --- | --- | --- |
| TCGA-D3-A8GB | 47.7059 | 0.364439387 | 48 | Alive | 3.5 | IV | NO | 4 | N/A | N/A | N/A |
| TCGA-FR-A8YC | 23.6857 | 0.372159955 | 78 | Dead | 2.6 | IV | YES | 16.6 | Stage IIIB | N1 | M0 |
| TCGA-GF-A6C8 | 27.0297 | 0.407355069 | 62 | Alive | 4 | IV | YES | 11 | Stage IIC | N0 | M0 |
| TCGA-ER-A19J | 50.831 | 0.46818538 | 54 | Alive | N/A | N/A | N/A | N/A | Stage II | N0 | M0 |
| TCGA-ER-A196 | 48.5513 | 0.468812349 | 64 | Alive | 22 | IV | YES | 10 | Stage IIIC | N1b | M0 |
| TCGA-EB-A551 | 41.4975 | 0.473148323 | 78 | Dead | 10 | V | YES | N/A | Stage IIA | N0 | M0 |
| TCGA-D3-A1Q9 | 14.4975 | 0.481810391 | 72 | Alive | 6 | V | YES | 10 | Stage IV | N1 | M1c |
| TCGA-XV-AAZY | 50.3331 | 0.494406803 | 76 | Alive | 16 | V | N/A | N/A | Stage IIIC | NX | M0 |
| TCGA-WE-AAA4 | 64.0884 | 0.497047977 | 56 | Dead | N/A | N/A | N/A | N/A | Stage IIIC | N3 | M0 |
| TCGA-EB-A431 | 56.5463 | 0.499103368 | 34 | Dead | 15 | III | YES | N/A | Stage IIIB | N1 | M0 |
| TCGA-D3-A2J8 | 53.701 | 0.521184707 | 48 | Alive | 1.4 | IV | NO | 1 | Stage IV | N0 | M1 |
| TCGA-FS-A1ZC | 15.6768 | 0.542692318 | 51 | Alive | N/A | III | N/A | N/A | Stage IIA | N0 | M0 |
| TCGA-EE-A2MK | 80.5875 | 0.549637767 | 18 | Alive | 5.2 | IV | NO | 8 | Stage IIIB | N1 | M0 |
| TCGA-EE-A3J7 | 72.8339 | 0.586293411 | 43 | Alive | 1.12 | II | YES | N/A | N/A | N/A | N/A |
| TCGA-DA-A1I0 | 10.6626 | 0.586301916 | 63 | Alive | 4.5 | IV | YES | N/A | Stage IIIC | N3 | M0 |
| TCGA-GN-A267 | 14.2591 | 0.604051841 | 38 | Alive | 5.2 | IV | NO | 8.6 | Stage IIC | N0 | N/A |
| TCGA-WE-A8ZO | 17.8445 | 0.605543569 | 73 | Alive | 2.4 | IV | NO | 14 | Stage IIC | N0 | M0 |
| TCGA-FS-A1Z3 | 58.3778 | 0.605562831 | 72 | Alive | N/A | N/A | N/A | N/A | Stage IIIC | N2b | M0 |
| TCGA-BF-A5ER | 46.2309 | 0.618590299 | 63 | Alive | 14 | IV | YES | N/A | Stage IIC | N0 | M0 |
| TCGA-D3-A1Q6 | 17.9558 | 0.636608192 | 55 | Dead | 50 | V | N/A | N/A | Stage IIB | N0 | M0 |
| TCGA-EB-A5SE | 43.402 | 0.637997297 | 73 | Dead | 4 | IV | YES | N/A | Stage IIIC | N3 | M0 |
| TCGA-ER-A19C | 76.0265 | 0.640247571 | 77 | Dead | 0.75 | III | NO | N/A | N/A | NX | M0 |
| TCGA-EB-A6QY | 34.3429 | 0.645056463 | 71 | Alive | 10 | N/A | YES | N/A | Stage IIC | N0 | M0 |
| TCGA-FS-A1Z4 | 57.7296 | 0.656500799 | 62 | Dead | 0.85 | III | NO | N/A | Stage IIC | N0 | M0 |
| TCGA-ER-A19E | 64.0924 | 0.664614671 | 36 | Alive | 1.1 | III | NO | N/A | Stage IIIB | N1a | M0 |
| TCGA-DA-A3F2 | 67.2691 | 0.679244167 | 55 | Alive | 5.1 | V | NO | N/A | Stage IV | N1b | M1b |
| TCGA-GN-A263 | 25.2127 | 0.680677254 | 24 | Alive | N/A | V | YES | 1.25 | Stage IIC | N0 | M0 |
| TCGA-EE-A3AG | 57.5849 | 0.681025376 | 25 | Dead | N/A | N/A | N/A | N/A | Stage II | N0 | M0 |
| TCGA-FR-A69P | 72.0641 | 0.681622092 | 34 | Dead | N/A | N/A | N/A | N/A | Stage III | N2c | M0 |
| TCGA-ER-A19M | 80.6271 | 0.698606979 | 36 | Alive | 1.9 | IV | NO | N/A | Stage IA | N0 | M0 |
| TCGA-EE-A2MP | 58.6963 | 0.701780324 | 34 | Alive | 1.05 | N/A | N/A | N/A | Stage III | N2 | M0 |
| TCGA-D3-A2JE | 4.4353 | 0.707625924 | 75 | Dead | N/A | N/A | N/A | N/A | Stage IB | N0 | M0 |
| TCGA-D3-A2JO | 7.3448 | 0.732891292 | 50 | Dead | N/A | N/A | YES | N/A | Stage IIB | N0 | M0 |
| TCGA-EE-A29P | 10.397 | 0.73627966 | 73 | Alive | 4.5 | IV | YES | 16 | Stage IIA | N0 | M0 |
| TCGA-FR-A3YO | 33.584 | 0.745829575 | N/A | Dead | N/A | N/A | N/A | N/A | Stage IIA | N0 | M0 |
| TCGA-FS-A1ZU | 61.1584 | 0.74811502 | 70 | Alive | 8.4 | V | YES | N/A | Stage IIIA | N1 | M0 |
| TCGA-EB-A5UL | 62.2137 | 0.762648872 | 71 | Alive | 4 | N/A | N/A | N/A | Stage IIIC | N3 | M0 |
| TCGA-EB-A1NK | 55.8562 | 0.763010801 | 48 | Alive | 8 | IV | YES | N/A | Stage IIC | N0 | M0 |
| TCGA-DA-A3F5 | 15.4272 | 0.772708274 | 45 | Alive | 0.4 | III | N/A | N/A | Stage IIIC | N1b | M0 |
| TCGA-EE-A2MD | 22.6663 | 0.781378402 | 52 | Dead | 3 | III | YES | 13 | Stage IIB | N0 | M0 |
| TCGA-DA-A1I5 | 64.3777 | 0.785431064 | 27 | Alive | 0.6 | IV | NO | N/A | Stage IIIB | N2b | M0 |
| TCGA-W3-AA1O | 23.1317 | 0.813037884 | 85 | Alive | N/A | N/A | N/A | N/A | Stage IIC | N0 | M0 |
| TCGA-FS-A1ZA | 12.9642 | 0.813408362 | 45 | Alive | 4.5 | IV | YES | N/A | Stage III | N1b | M0 |
| TCGA-EB-A5FP | 24.5929 | 0.81719747 | 65 | Dead | 8 | N/A | YES | N/A | Stage IIIC | N3 | M0 |
| TCGA-EE-A2MM | 20.0238 | 0.821423337 | 63 | Alive | 0.6 | III | N/A | 1 | Stage IIIC | N1b | M0 |
| TCGA-EE-A2M5 | 32.0893 | 0.830207935 | 49 | Dead | 0.8 | III | NO | 1 | Stage IIIB | N2c | M0 |
| TCGA-FW-A3TV | 77.8073 | 0.83490106 | 57 | Dead | 0.5 | III | N/A | N/A | Stage IIIB | N1a | M0 |
| TCGA-EE-A185 | 60.221 | 0.844757832 | 55 | Dead | 6 | IV | YES | 12 | Stage 0 | N0 | M0 |
| TCGA-EE-A29R | 35.1613 | 0.847015844 | 48 | Alive | 3.5 | V | YES | 6 | Stage IIC | N0 | M0 |
| TCGA-DA-A95Z | 79.4798 | 0.852715709 | 87 | Alive | N/A | N/A | N/A | N/A | Stage IB | N0 | M0 |
| TCGA-EE-A2MU | 23.5918 | 0.866758906 | 71 | Dead | 0.5 | II | NO | 2 | Stage IV | N3 | M1c |
| TCGA-D3-A8GK | 11.2374 | 0.880859179 | 45 | Dead | 2.6 | IV | NO | 5 | Stage IIIC | N1b | M0 |
| TCGA-GF-A3OT | 70.3384 | 0.885172796 | 58 | Dead | N/A | N/A | N/A | N/A | Stage IIIC | N3 | M0 |
| TCGA-EE-A29C | 60.2811 | 0.887492322 | 20 | Alive | 1.7 | IV | NO | 4 | Stage IIC | N0 | M0 |
| TCGA-EB-A3XC | 21.0364 | 0.892218829 | 74 | Dead | N/A | N/A | YES | N/A | N/A | NX | M1 |
| TCGA-DA-A1I4 | 51.5053 | 0.898230717 | 51 | Dead | 3.4 | IV | YES | N/A | Stage IIC | N0 | M0 |
| TCGA-D9-A1JW | 80.5399 | 0.900945485 | 82 | Alive | N/A | N/A | NO | N/A | Stage IIC | N0 | M0 |
| TCGA-FR-A3R1 | 35.6192 | 0.902633718 | 69 | Alive | 6.3 | V | YES | 3.75 | Stage IIIB | N/A | M0 |
| TCGA-D3-A2JD | 64.3016 | 0.902668146 | 58 | Alive | 18 | V | YES | 14 | Stage IIIC | N1b | M0 |
| TCGA-EE-A2GU | 48.4107 | 0.905655321 | 65 | Alive | 0.3 | II | NO | 0 | N/A | NX | M0 |
| TCGA-EB-A44O | 66.6015 | 0.907539684 | 69 | Dead | 5 | N/A | NO | N/A | Stage IB | N0 | M0 |
| TCGA-EE-A29L | 36.8044 | 0.91734874 | 78 | Alive | 7 | V | YES | 16 | Stage IB | N0 | M0 |
| TCGA-ER-A2NB | 75.8964 | 0.92343612 | 57 | Alive | 4.39 | IV | YES | 1.3 | N/A | NX | M0 |
| TCGA-EE-A29E | 56.4746 | 0.92881909 | 54 | Alive | 3.2 | IV | NO | 6 | Stage IIIA | N1 | M0 |
| TCGA-D3-A3CE | 47.8863 | 0.941517916 | 74 | Alive | N/A | N/A | N/A | N/A | Stage IIB | N0 | M0 |
| TCGA-D9-A3Z1 | 14.0332 | 0.941837459 | 66 | Dead | 1.7 | IV | N/A | N/A | Stage IIIA | N1a | M0 |
| TCGA-ER-A2NF | 67.0181 | 0.943934846 | 53 | Dead | 4 | V | YES | N/A | Stage IA | N0 | M0 |
| TCGA-GN-A26A | 14.861 | 0.950419381 | 63 | Alive | 2.3 | IV | NO | 11 | Stage IB | N0 | M0 |
| TCGA-FS-A1ZT | 18.3039 | 0.961878802 | 55 | Alive | 1.15 | IV | N/A | N/A | Stage II | N0 | M0 |
| TCGA-FR-A44A | 34.4612 | 0.983450936 | 29 | Alive | 2.51 | IV | NO | 9 | Stage IIC | N0 | M0 |
| TCGA-RP-A694 | 46.1913 | 0.985578611 | 71 | Dead | N/A | N/A | N/A | N/A | Stage IB | NX | N/A |
| TCGA-EE-A3AB | 23.7174 | 0.985832919 | 30 | Dead | N/A | N/A | N/A | N/A | Stage IIC | N0 | M0 |
| TCGA-D3-A51N | 48.9642 | 0.998202952 | 56 | Dead | N/A | N/A | N/A | N/A | Stage IIIB | N2b | M0 |
| TCGA-FS-A1ZM | 68.1376 | 1.004065271 | 74 | Dead | 1.2 | IV | N/A | N/A | Stage IIIB | N1b | M0 |
| TCGA-GN-A4U7 | 16.2049 | 1.02634016 | 56 | Dead | 1.39 | IV | NO | 8 | Stage IA | N0 | M0 |
| TCGA-HR-A2OH | 74.9415 | 1.028462499 | 46 | Alive | 3.4 | N/A | YES | N/A | Stage IIIC | N3 | M0 |
| TCGA-WE-A8K6 | 20.1688 | 1.038624359 | 79 | Dead | N/A | N/A | N/A | N/A | Stage IIC | NX | M0 |
| TCGA-EE-A29H | 59.9993 | 1.03933165 | 59 | Alive | 1 | II | NO | 1 | Stage IIIC | N3 | M0 |
| TCGA-D3-A8GP | 20.1635 | 1.043112106 | 77 | Alive | 1.8 | IV | N/A | N/A | Stage IIB | N0 | M0 |
| TCGA-FS-A1ZZ | 8.4784 | 1.045456839 | 54 | Dead | 3.8 | IV | YES | N/A | Stage II | N0 | M0 |
| TCGA-D9-A6EA | 39.8671 | 1.046062246 | 70 | Alive | 6 | IV | NO | N/A | Stage I | N0 | M0 |
| TCGA-D3-A51E | 6.8729 | 1.053596566 | 39 | Alive | 1.2 | III | N/A | N/A | Stage IIA | N0 | M0 |
| TCGA-D3-A3CB | 66.0421 | 1.093763082 | 39 | Alive | 1.75 | III | N/A | N/A | Stage I | N0 | M0 |
| TCGA-BF-A1PZ | 46.4441 | 1.096267552 | 71 | Alive | 8 | III | NO | N/A | Stage IIB | N0 | M0 |
| TCGA-DA-A3F3 | 36.3531 | 1.100495294 | 52 | Alive | N/A | N/A | N/A | N/A | Stage IIIB | N2 | M0 |
| TCGA-XV-AAZV | 32.9937 | 1.111136626 | 56 | Alive | 10 | IV | N/A | N/A | Stage IIIB | NX | M0 |
| TCGA-DA-A95Y | 74.3142 | 1.139393417 | 68 | Alive | 5.03 | IV | YES | N/A | N/A | N/A | N/A |
| TCGA-BF-AAP1 | 57.6238 | 1.147896603 | 86 | Dead | 15 | IV | YES | N/A | Stage IB | NX | M0 |
| TCGA-D3-A51H | 61.9413 | 1.157195695 | 60 | Alive | 0.91 | IV | YES | 0 | Stage IB | N0 | M0 |
| TCGA-3N-A9WD | 75.8865 | 1.15894231 | 82 | Alive | 1.25 | III | NO | 4 | Stage IIIC | N3 | M0 |
| TCGA-D3-A8GD | 20.9115 | 1.172183121 | 63 | Alive | 20 | IV | YES | 18 | Stage IIC | N0 | M0 |
| TCGA-D3-A8GC | 48.0458 | 1.186773249 | 48 | Alive | N/A | N/A | N/A | N/A | Stage 0 | N0 | M0 |
| TCGA-RP-A695 | 46.6508 | 1.205734142 | N/A | Alive | N/A | N/A | N/A | N/A | Stage IIC | N0 | M0 |
| TCGA-D3-A3ML | 23.1179 | 1.207619436 | 70 | Dead | 2.3 | IV | NO | 3 | Stage III | N0 | M0 |
| TCGA-EE-A2MC | 46.6051 | 1.21948056 | 73 | Alive | 1 | III | NO | 2 | Stage IV | NX | M1 |
| TCGA-EE-A3AC | 4.855 | 1.236992532 | 47 | Dead | N/A | N/A | N/A | N/A | Stage IIIC | N3 | M0 |
| TCGA-GF-A4EO | 29.5445 | 1.250798704 | 74 | Dead | N/A | N/A | N/A | N/A | Stage IIC | N0 | M0 |
| TCGA-QB-A6FS | 25.3019 | 1.279324556 | 49 | Dead | N/A | N/A | N/A | N/A | N/A | N/A | N/A |
| TCGA-EE-A3J3 | 53.5428 | 1.282629796 | 42 | Alive | 0.98 | N/A | N/A | N/A | Stage IV | N0 | M1 |
| TCGA-ER-A3PL | 64.1874 | 1.283528815 | 30 | Dead | 2.2 | V | YES | N/A | Stage IV | N3 | M1a |
| TCGA-EE-A2MT | 39.3851 | 1.293266936 | 45 | Alive | 1.5 | IV | NO | 4 | Stage IIIC | N3 | M0 |
| TCGA-FR-A728 | 64.2553 | 1.324608346 | 54 | Dead | 12 | IV | YES | 6 | Stage IIIB | N1a | M0 |
| TCGA-D3-A8GR | 39.5108 | 1.327965088 | 54 | Alive | 0.01 | I | N/A | N/A | Stage II | N0 | M0 |
| TCGA-EE-A2GR | 29.6606 | 1.330980867 | 78 | Alive | 6.9 | IV | N/A | 8 | Stage IIIC | N3 | M0 |
| TCGA-EE-A3AA | 10.6452 | 1.332283701 | 47 | Dead | N/A | N/A | N/A | N/A | Stage IIIC | N3 | M0 |
| TCGA-EE-A3JA | 28.2486 | 1.37094586 | 44 | Alive | 1.5 | IV | NO | N/A | N/A | NX | M0 |
| TCGA-EE-A2ML | 21.8307 | 1.391565465 | 35 | Alive | 3 | III | NO | 8 | Stage I | N0 | M0 |
| TCGA-DA-A95X | 35.3288 | 1.441735414 | 62 | Dead | 1.45 | IV | NO | N/A | Stage IIIB | N2a | M0 |
| TCGA-DA-A1HV | 14.3121 | 1.441935692 | 75 | Dead | N/A | N/A | N/A | N/A | Stage I | N/A | N/A |
| TCGA-D3-A1QB | 76.8931 | 1.451761402 | 75 | Dead | N/A | N/A | N/A | N/A | Stage IIB | N0 | M0 |
| TCGA-ER-A2ND | 44.0035 | 1.452366115 | 57 | Alive | 1 | IV | NO | N/A | Stage IA | N0 | M0 |
| TCGA-W3-AA1Q | 14.6048 | 1.476316241 | 57 | Alive | N/A | N/A | N/A | N/A | Stage IIA | N0 | M0 |
| TCGA-EE-A29S | 32.6185 | 1.56741067 | 79 | Alive | 2.5 | IV | NO | 7 | Stage IIIC | N3 | M0 |
| TCGA-EE-A3AF | 15.0231 | 1.581047048 | 48 | Dead | N/A | N/A | N/A | N/A | Stage III | N1b | M0 |
| TCGA-ER-A19Q | 30.6921 | 1.606766092 | 37 | Alive | 1.2 | III | NO | N/A | Stage IIIB | N1 | M0 |
| TCGA-GN-A26C | 18.2371 | 1.647294339 | 77 | Alive | 14 | IV | YES | 0.4 | Stage IA | N0 | M0 |
| TCGA-EE-A3AD | 51.3177 | 1.750854856 | 50 | Dead | N/A | N/A | N/A | N/A | Stage II | N0 | M0 |
| TCGA-BF-A5ES | 54.3353 | 1.847871727 | 76 | Alive | 8 | IV | YES | N/A | Stage IIC | N0 | M0 |
| TCGA-EB-A24D | 27.8878 | 1.878242954 | 72 | Dead | 36 | N/A | NO | N/A | Stage IB | N0 | M0 |
| TCGA-ER-A194 | 35.9112 | 1.93544897 | 77 | Dead | 4.5 | IV | YES | 1 | Stage IIIC | N1b | M0 |
| TCGA-ER-A19F | 14.9312 | 1.984985521 | 82 | Dead | 6.7 | N/A | N/A | N/A | Stage IIA | N0 | M0 |
| TCGA-D3-A2JP | 22.0588 | 1.993682524 | 37 | Dead | N/A | N/A | N/A | N/A | Stage IA | N0 | M0 |
| TCGA-ER-A19G | 80.8197 | 2.021978935 | 48 | Alive | 1.6 | II | NO | N/A | Stage IIIC | N3 | M0 |
| TCGA-ER-A3ET | 60.0009 | 2.208525786 | 64 | Alive | 3.55 | IV | N/A | N/A | Stage III | N2 | M0 |
| TCGA-DA-A1HY | 49.6648 | 2.2100743 | 42 | Alive | 1.13 | IV | YES | N/A | Stage IIIC | N1b | M0 |
| TCGA-GN-A8LN | 64.7635 | 2.365177915 | 68 | Alive | 4.85 | IV | YES | 1.6 | Stage I | N0 | M0 |
| TCGA-D3-A3C3 | 9.5334 | 3.989290856 | N/A | Alive | N/A | N/A | N/A | N/A | Stage IIC | N0 | M0 |

**Supplementary Table S3⏐List of proteins with increased binding to β-catenin from IP-MS analysis.**

| Gene ID | Mapped ID | Gene Name/Symbol | GO Biological Process |
| --- | --- | --- | --- |
| [HUMAN\|Ensembl=ENSG00000021776\|UniProtKB=O60306](http://www.pantherdb.org/genes/gene.do?acc=HUMAN%7CEnsembl=ENSG00000021776%7CUniProtKB=O60306) | O60306 | Intron-binding protein aquarius  [AQR](http://www.pantherdb.org/genes/gene.do?acc=HUMAN%7CEnsembl=ENSG00000021776%7CUniProtKB=O60306)  [ortholog](http://www.pantherdb.org/genes/gene.do?acc=HUMAN%7CEnsembl=ENSG00000021776%7CUniProtKB=O60306#orthologs) | [DNA replication](http://www.pantherdb.org/panther/category.do?categoryAcc=GO:0006260)  [transcription from RNA polymerase II promoter](http://www.pantherdb.org/panther/category.do?categoryAcc=GO:0006366)  [mRNA splicing, via spliceosome](http://www.pantherdb.org/panther/category.do?categoryAcc=GO:0000398)  [tRNA metabolic process](http://www.pantherdb.org/panther/category.do?categoryAcc=GO:0006399)  [RNA catabolic process](http://www.pantherdb.org/panther/category.do?categoryAcc=GO:0006401)  [protein metabolic process](http://www.pantherdb.org/panther/category.do?categoryAcc=GO:0019538)  [cell cycle](http://www.pantherdb.org/panther/category.do?categoryAcc=GO:0007049)  [regulation of transcription from RNA polymerase II promoter](http://www.pantherdb.org/panther/category.do?categoryAcc=GO:0006357) |
| [HUMAN\|Ensembl=ENSG00000144028\|UniProtKB=O75643](http://www.pantherdb.org/genes/gene.do?acc=HUMAN%7CEnsembl=ENSG00000144028%7CUniProtKB=O75643) | O75643 | U5 small nuclear ribonucleoprotein 200 kDa helicase  [SNRNP200](http://www.pantherdb.org/genes/gene.do?acc=HUMAN%7CEnsembl=ENSG00000144028%7CUniProtKB=O75643)  [ortholog](http://www.pantherdb.org/genes/gene.do?acc=HUMAN%7CEnsembl=ENSG00000144028%7CUniProtKB=O75643#orthologs) | [RNA splicing, via transesterification reactions](http://www.pantherdb.org/panther/category.do?categoryAcc=GO:0000375)  [mRNA splicing, via spliceosome](http://www.pantherdb.org/panther/category.do?categoryAcc=GO:0000398)  [RNA splicing, via transesterification reactions](http://www.pantherdb.org/panther/category.do?categoryAcc=GO:0000375)  [meiosis](http://www.pantherdb.org/panther/category.do?categoryAcc=GO:0007126) |
| [HUMAN\|Ensembl=ENSG00000100591\|UniProtKB=O95433](http://www.pantherdb.org/genes/gene.do?acc=HUMAN%7CEnsembl=ENSG00000100591%7CUniProtKB=O95433) | O95433 | Activator of 90 kDa heat shock protein ATPase homolog 1  [AHSA1](http://www.pantherdb.org/genes/gene.do?acc=HUMAN%7CEnsembl=ENSG00000100591%7CUniProtKB=O95433)  [ortholog](http://www.pantherdb.org/genes/gene.do?acc=HUMAN%7CEnsembl=ENSG00000100591%7CUniProtKB=O95433#orthologs) | [immune system process](http://www.pantherdb.org/panther/category.do?categoryAcc=GO:0002376)  [protein folding](http://www.pantherdb.org/panther/category.do?categoryAcc=GO:0006457)  [response to stress](http://www.pantherdb.org/panther/category.do?categoryAcc=GO:0006950)  [regulation of catalytic activity](http://www.pantherdb.org/panther/category.do?categoryAcc=GO:0050790) |
| [HUMAN\|Ensembl=ENSG00000189060\|UniProtKB=P07305](http://www.pantherdb.org/genes/gene.do?acc=HUMAN%7CEnsembl=ENSG00000189060%7CUniProtKB=P07305) | P07305 | Histone H1.0  [H1F0](http://www.pantherdb.org/genes/gene.do?acc=HUMAN%7CEnsembl=ENSG00000189060%7CUniProtKB=P07305)  [ortholog](http://www.pantherdb.org/genes/gene.do?acc=HUMAN%7CEnsembl=ENSG00000189060%7CUniProtKB=P07305#orthologs) | [nucleobase-containing compound metabolic process](http://www.pantherdb.org/panther/category.do?categoryAcc=GO:0006139)  [cellular process](http://www.pantherdb.org/panther/category.do?categoryAcc=GO:0009987)  [chromatin organization](http://www.pantherdb.org/panther/category.do?categoryAcc=GO:0006325) |
| [HUMAN\|Ensembl=ENSG00000124228\|UniProtKB=Q96GQ7](http://www.pantherdb.org/genes/gene.do?acc=HUMAN%7CEnsembl=ENSG00000124228%7CUniProtKB=Q96GQ7) | Q96GQ7 | Probable ATP-dependent RNA helicase DDX27  [DDX27](http://www.pantherdb.org/genes/gene.do?acc=HUMAN%7CEnsembl=ENSG00000124228%7CUniProtKB=Q96GQ7)  [ortholog](http://www.pantherdb.org/genes/gene.do?acc=HUMAN%7CEnsembl=ENSG00000124228%7CUniProtKB=Q96GQ7#orthologs) | [nucleobase-containing compound metabolic process](http://www.pantherdb.org/panther/category.do?categoryAcc=GO:0006139)  [translation](http://www.pantherdb.org/panther/category.do?categoryAcc=GO:0006412)  [regulation of translation](http://www.pantherdb.org/panther/category.do?categoryAcc=GO:0006417) |
| [HUMAN\|Ensembl=ENSG00000136271\|UniProtKB=Q9NY93](http://www.pantherdb.org/genes/gene.do?acc=HUMAN%7CEnsembl=ENSG00000136271%7CUniProtKB=Q9NY93) | Q9NY93 | Probable ATP-dependent RNA helicase DDX56  [DDX56](http://www.pantherdb.org/genes/gene.do?acc=HUMAN%7CEnsembl=ENSG00000136271%7CUniProtKB=Q9NY93)  [ortholog](http://www.pantherdb.org/genes/gene.do?acc=HUMAN%7CEnsembl=ENSG00000136271%7CUniProtKB=Q9NY93#orthologs) | [nucleobase-containing compound metabolic process](http://www.pantherdb.org/panther/category.do?categoryAcc=GO:0006139)  [translation](http://www.pantherdb.org/panther/category.do?categoryAcc=GO:0006412)  [regulation of translation](http://www.pantherdb.org/panther/category.do?categoryAcc=GO:0006417) |
| [HUMAN\|Ensembl=ENSG00000183520\|UniProtKB=Q9Y3A2](http://www.pantherdb.org/genes/gene.do?acc=HUMAN%7CEnsembl=ENSG00000183520%7CUniProtKB=Q9Y3A2) | Q9Y3A2 | Probable U3 small nucleolar RNA-associated protein 11  [UTP11L](http://www.pantherdb.org/genes/gene.do?acc=HUMAN%7CEnsembl=ENSG00000183520%7CUniProtKB=Q9Y3A2)  [ortholog](http://www.pantherdb.org/genes/gene.do?acc=HUMAN%7CEnsembl=ENSG00000183520%7CUniProtKB=Q9Y3A2#orthologs) | [nucleobase-containing compound metabolic process](http://www.pantherdb.org/panther/category.do?categoryAcc=GO:0006139) |
| [HUMAN\|Ensembl=ENSG00000215301\|UniProtKB=O00571](http://www.pantherdb.org/genes/gene.do?acc=HUMAN%7CEnsembl=ENSG00000215301%7CUniProtKB=O00571) | O00571 | ATP-dependent RNA helicase DDX3X  [DDX3X](http://www.pantherdb.org/genes/gene.do?acc=HUMAN%7CEnsembl=ENSG00000215301%7CUniProtKB=O00571)  [ortholog](http://www.pantherdb.org/genes/gene.do?acc=HUMAN%7CEnsembl=ENSG00000215301%7CUniProtKB=O00571#orthologs) | [nucleobase-containing compound metabolic process](http://www.pantherdb.org/panther/category.do?categoryAcc=GO:0006139)  [translation](http://www.pantherdb.org/panther/category.do?categoryAcc=GO:0006412)  [regulation of translation](http://www.pantherdb.org/panther/category.do?categoryAcc=GO:0006417) |
| [HUMAN\|Ensembl=ENSG00000102978\|UniProtKB=P19387](http://www.pantherdb.org/genes/gene.do?acc=HUMAN%7CEnsembl=ENSG00000102978%7CUniProtKB=P19387) | P19387 | DNA-directed RNA polymerase II subunit RPB3  [POLR2C](http://www.pantherdb.org/genes/gene.do?acc=HUMAN%7CEnsembl=ENSG00000102978%7CUniProtKB=P19387)  [ortholog](http://www.pantherdb.org/genes/gene.do?acc=HUMAN%7CEnsembl=ENSG00000102978%7CUniProtKB=P19387#orthologs) | [transcription from RNA polymerase II promoter](http://www.pantherdb.org/panther/category.do?categoryAcc=GO:0006366) |
| [HUMAN\|Ensembl=ENSG00000096746\|UniProtKB=P31942](http://www.pantherdb.org/genes/gene.do?acc=HUMAN%7CEnsembl=ENSG00000096746%7CUniProtKB=P31942) | P31942 | Heterogeneous nuclear ribonucleoprotein H3  [HNRNPH3](http://www.pantherdb.org/genes/gene.do?acc=HUMAN%7CEnsembl=ENSG00000096746%7CUniProtKB=P31942)  [ortholog](http://www.pantherdb.org/genes/gene.do?acc=HUMAN%7CEnsembl=ENSG00000096746%7CUniProtKB=P31942#orthologs) | [mRNA splicing, via spliceosome](http://www.pantherdb.org/panther/category.do?categoryAcc=GO:0000398) |
| [HUMAN\|Ensembl=ENSG00000143621\|UniProtKB=Q12905](http://www.pantherdb.org/genes/gene.do?acc=HUMAN%7CEnsembl=ENSG00000143621%7CUniProtKB=Q12905) | Q12905 | Interleukin enhancer-binding factor 2  [ILF2](http://www.pantherdb.org/genes/gene.do?acc=HUMAN%7CEnsembl=ENSG00000143621%7CUniProtKB=Q12905)  [ortholog](http://www.pantherdb.org/genes/gene.do?acc=HUMAN%7CEnsembl=ENSG00000143621%7CUniProtKB=Q12905#orthologs) | [spermatogenesis](http://www.pantherdb.org/panther/category.do?categoryAcc=GO:0007283)  [response to interferon-gamma](http://www.pantherdb.org/panther/category.do?categoryAcc=GO:0034341)  [apoptotic process](http://www.pantherdb.org/panther/category.do?categoryAcc=GO:0006915)  [purine nucleobase metabolic process](http://www.pantherdb.org/panther/category.do?categoryAcc=GO:0006144)  [protein metabolic process](http://www.pantherdb.org/panther/category.do?categoryAcc=GO:0019538)  [cell cycle](http://www.pantherdb.org/panther/category.do?categoryAcc=GO:0007049)  [apoptotic process](http://www.pantherdb.org/panther/category.do?categoryAcc=GO:0006915)  [response to stimulus](http://www.pantherdb.org/panther/category.do?categoryAcc=GO:0050896)  [RNA localization](http://www.pantherdb.org/panther/category.do?categoryAcc=GO:0006403)  [regulation of catalytic activity](http://www.pantherdb.org/panther/category.do?categoryAcc=GO:0050790) |
| [HUMAN\|Ensembl=ENSG00000132153\|UniProtKB=Q7L2E3](http://www.pantherdb.org/genes/gene.do?acc=HUMAN%7CEnsembl=ENSG00000132153%7CUniProtKB=Q7L2E3) | Q7L2E3 | Putative ATP-dependent RNA helicase DHX30  [DHX30](http://www.pantherdb.org/genes/gene.do?acc=HUMAN%7CEnsembl=ENSG00000132153%7CUniProtKB=Q7L2E3)  [ortholog](http://www.pantherdb.org/genes/gene.do?acc=HUMAN%7CEnsembl=ENSG00000132153%7CUniProtKB=Q7L2E3#orthologs) | [mRNA splicing, via spliceosome](http://www.pantherdb.org/panther/category.do?categoryAcc=GO:0000398) |
| [HUMAN\|Ensembl=ENSG00000151893\|UniProtKB=Q86Y37](http://www.pantherdb.org/genes/gene.do?acc=HUMAN%7CEnsembl=ENSG00000151893%7CUniProtKB=Q86Y37) | Q86Y37 | CDK2-associated and cullin domain-containing protein 1  [CACUL1](http://www.pantherdb.org/genes/gene.do?acc=HUMAN%7CEnsembl=ENSG00000151893%7CUniProtKB=Q86Y37)  [ortholog](http://www.pantherdb.org/genes/gene.do?acc=HUMAN%7CEnsembl=ENSG00000151893%7CUniProtKB=Q86Y37#orthologs) | [induction of apoptosis](http://www.pantherdb.org/panther/category.do?categoryAcc=GO:0006917)  [proteolysis](http://www.pantherdb.org/panther/category.do?categoryAcc=GO:0006508)  [mitosis](http://www.pantherdb.org/panther/category.do?categoryAcc=GO:0007067)  [induction of apoptosis](http://www.pantherdb.org/panther/category.do?categoryAcc=GO:0006917) |
| [HUMAN\|Ensembl=ENSG00000119285\|UniProtKB=Q9H583](http://www.pantherdb.org/genes/gene.do?acc=HUMAN%7CEnsembl=ENSG00000119285%7CUniProtKB=Q9H583) | Q9H583 | HEAT repeat-containing protein 1  [HEATR1](http://www.pantherdb.org/genes/gene.do?acc=HUMAN%7CEnsembl=ENSG00000119285%7CUniProtKB=Q9H583)  [ortholog](http://www.pantherdb.org/genes/gene.do?acc=HUMAN%7CEnsembl=ENSG00000119285%7CUniProtKB=Q9H583#orthologs) | [rRNA metabolic process](http://www.pantherdb.org/panther/category.do?categoryAcc=GO:0016072) |
| [HUMAN\|Ensembl=ENSG00000106344\|UniProtKB=Q9NW13](http://www.pantherdb.org/genes/gene.do?acc=HUMAN%7CEnsembl=ENSG00000106344%7CUniProtKB=Q9NW13) | Q9NW13 | RNA-binding protein 28  [RBM28](http://www.pantherdb.org/genes/gene.do?acc=HUMAN%7CEnsembl=ENSG00000106344%7CUniProtKB=Q9NW13)  [ortholog](http://www.pantherdb.org/genes/gene.do?acc=HUMAN%7CEnsembl=ENSG00000106344%7CUniProtKB=Q9NW13#orthologs) | [DNA replication](http://www.pantherdb.org/panther/category.do?categoryAcc=GO:0006260)  [RNA splicing, via transesterification reactions](http://www.pantherdb.org/panther/category.do?categoryAcc=GO:0000375)  [mRNA splicing, via spliceosome](http://www.pantherdb.org/panther/category.do?categoryAcc=GO:0000398)  [mRNA polyadenylation](http://www.pantherdb.org/panther/category.do?categoryAcc=GO:0006378)  [RNA splicing, via transesterification reactions](http://www.pantherdb.org/panther/category.do?categoryAcc=GO:0000375)  [rRNA metabolic process](http://www.pantherdb.org/panther/category.do?categoryAcc=GO:0016072)  [protein metabolic process](http://www.pantherdb.org/panther/category.do?categoryAcc=GO:0019538)  [cell cycle](http://www.pantherdb.org/panther/category.do?categoryAcc=GO:0007049)  [neurological system process](http://www.pantherdb.org/panther/category.do?categoryAcc=GO:0050877)  [ectoderm development](http://www.pantherdb.org/panther/category.do?categoryAcc=GO:0007398) |
| [HUMAN\|Ensembl=ENSG00000159217\|UniProtKB=Q9NZI8](http://www.pantherdb.org/genes/gene.do?acc=HUMAN%7CEnsembl=ENSG00000159217%7CUniProtKB=Q9NZI8) | Q9NZI8 | Insulin-like growth factor 2 mRNA-binding protein 1  [IGF2BP1](http://www.pantherdb.org/genes/gene.do?acc=HUMAN%7CEnsembl=ENSG00000159217%7CUniProtKB=Q9NZI8)  [ortholog](http://www.pantherdb.org/genes/gene.do?acc=HUMAN%7CEnsembl=ENSG00000159217%7CUniProtKB=Q9NZI8#orthologs) | [induction of apoptosis](http://www.pantherdb.org/panther/category.do?categoryAcc=GO:0006917)  [RNA splicing, via transesterification reactions](http://www.pantherdb.org/panther/category.do?categoryAcc=GO:0000375)  [transcription from RNA polymerase II promoter](http://www.pantherdb.org/panther/category.do?categoryAcc=GO:0006366)  [mRNA splicing, via spliceosome](http://www.pantherdb.org/panther/category.do?categoryAcc=GO:0000398)  [RNA splicing, via transesterification reactions](http://www.pantherdb.org/panther/category.do?categoryAcc=GO:0000375)  [protein metabolic process](http://www.pantherdb.org/panther/category.do?categoryAcc=GO:0019538)  [cell communication](http://www.pantherdb.org/panther/category.do?categoryAcc=GO:0007154)  [neurological system process](http://www.pantherdb.org/panther/category.do?categoryAcc=GO:0050877)  [induction of apoptosis](http://www.pantherdb.org/panther/category.do?categoryAcc=GO:0006917)  [intracellular protein transport](http://www.pantherdb.org/panther/category.do?categoryAcc=GO:0006886)  [nuclear transport](http://www.pantherdb.org/panther/category.do?categoryAcc=GO:0051169) |
| [HUMAN\|Ensembl=ENSG00000125970\|UniProtKB=Q9UKM9](http://www.pantherdb.org/genes/gene.do?acc=HUMAN%7CEnsembl=ENSG00000125970%7CUniProtKB=Q9UKM9) | Q9UKM9 | RNA-binding protein Raly  [RALY](http://www.pantherdb.org/genes/gene.do?acc=HUMAN%7CEnsembl=ENSG00000125970%7CUniProtKB=Q9UKM9)  [ortholog](http://www.pantherdb.org/genes/gene.do?acc=HUMAN%7CEnsembl=ENSG00000125970%7CUniProtKB=Q9UKM9#orthologs) | [mRNA splicing, via spliceosome](http://www.pantherdb.org/panther/category.do?categoryAcc=GO:0000398) |
| [HUMAN\|Ensembl=ENSG00000153147\|UniProtKB=O60264](http://www.pantherdb.org/genes/gene.do?acc=HUMAN%7CEnsembl=ENSG00000153147%7CUniProtKB=O60264) | O60264 | SWI/SNF-related matrix-associated actin-dependent regulator of chromatin subfamily A member 5  [SMARCA5](http://www.pantherdb.org/genes/gene.do?acc=HUMAN%7CEnsembl=ENSG00000153147%7CUniProtKB=O60264)  [ortholog](http://www.pantherdb.org/genes/gene.do?acc=HUMAN%7CEnsembl=ENSG00000153147%7CUniProtKB=O60264#orthologs) | [DNA repair](http://www.pantherdb.org/panther/category.do?categoryAcc=GO:0006281)  [DNA recombination](http://www.pantherdb.org/panther/category.do?categoryAcc=GO:0006310)  [transcription from RNA polymerase II promoter](http://www.pantherdb.org/panther/category.do?categoryAcc=GO:0006366)  [cellular process](http://www.pantherdb.org/panther/category.do?categoryAcc=GO:0009987)  [regulation of transcription from RNA polymerase II promoter](http://www.pantherdb.org/panther/category.do?categoryAcc=GO:0006357)  [chromatin organization](http://www.pantherdb.org/panther/category.do?categoryAcc=GO:0006325) |
| [HUMAN\|Ensembl=ENSG00000092199\|UniProtKB=P07910](http://www.pantherdb.org/genes/gene.do?acc=HUMAN%7CEnsembl=ENSG00000092199%7CUniProtKB=P07910) | P07910 | Heterogeneous nuclear ribonucleoproteins C1/C2  [HNRNPC](http://www.pantherdb.org/genes/gene.do?acc=HUMAN%7CEnsembl=ENSG00000092199%7CUniProtKB=P07910)  [ortholog](http://www.pantherdb.org/genes/gene.do?acc=HUMAN%7CEnsembl=ENSG00000092199%7CUniProtKB=P07910#orthologs) | [mRNA splicing, via spliceosome](http://www.pantherdb.org/panther/category.do?categoryAcc=GO:0000398) |
| [HUMAN\|Ensembl=ENSG00000099783\|UniProtKB=P52272](http://www.pantherdb.org/genes/gene.do?acc=HUMAN%7CEnsembl=ENSG00000099783%7CUniProtKB=P52272) | P52272 | Heterogeneous nuclear ribonucleoprotein M  [HNRNPM](http://www.pantherdb.org/genes/gene.do?acc=HUMAN%7CEnsembl=ENSG00000099783%7CUniProtKB=P52272)  [ortholog](http://www.pantherdb.org/genes/gene.do?acc=HUMAN%7CEnsembl=ENSG00000099783%7CUniProtKB=P52272#orthologs) | [mRNA splicing, via spliceosome](http://www.pantherdb.org/panther/category.do?categoryAcc=GO:0000398) |
| [HUMAN\|Ensembl=ENSG00000108468\|UniProtKB=P83916](http://www.pantherdb.org/genes/gene.do?acc=HUMAN%7CEnsembl=ENSG00000108468%7CUniProtKB=P83916) | P83916 | Chromobox protein homolog 1  [CBX1](http://www.pantherdb.org/genes/gene.do?acc=HUMAN%7CEnsembl=ENSG00000108468%7CUniProtKB=P83916)  [ortholog](http://www.pantherdb.org/genes/gene.do?acc=HUMAN%7CEnsembl=ENSG00000108468%7CUniProtKB=P83916#orthologs) | [apoptotic process](http://www.pantherdb.org/panther/category.do?categoryAcc=GO:0006915)  [transcription from RNA polymerase II promoter](http://www.pantherdb.org/panther/category.do?categoryAcc=GO:0006366)  [cellular process](http://www.pantherdb.org/panther/category.do?categoryAcc=GO:0009987)  [apoptotic process](http://www.pantherdb.org/panther/category.do?categoryAcc=GO:0006915)  [regulation of transcription from RNA polymerase II promoter](http://www.pantherdb.org/panther/category.do?categoryAcc=GO:0006357)  [chromatin organization](http://www.pantherdb.org/panther/category.do?categoryAcc=GO:0006325) |
| [HUMAN\|Ensembl=ENSG00000113460\|UniProtKB=Q8TDN6](http://www.pantherdb.org/genes/gene.do?acc=HUMAN%7CEnsembl=ENSG00000113460%7CUniProtKB=Q8TDN6) | Q8TDN6 | Ribosome biogenesis protein BRX1 homolog  [BRIX1](http://www.pantherdb.org/genes/gene.do?acc=HUMAN%7CEnsembl=ENSG00000113460%7CUniProtKB=Q8TDN6)  [ortholog](http://www.pantherdb.org/genes/gene.do?acc=HUMAN%7CEnsembl=ENSG00000113460%7CUniProtKB=Q8TDN6#orthologs) | [rRNA metabolic process](http://www.pantherdb.org/panther/category.do?categoryAcc=GO:0016072) |
| [HUMAN\|Ensembl=ENSG00000107937\|UniProtKB=Q9BZE4](http://www.pantherdb.org/genes/gene.do?acc=HUMAN%7CEnsembl=ENSG00000107937%7CUniProtKB=Q9BZE4) | Q9BZE4 | Nucleolar GTP-binding protein 1  [GTPBP4](http://www.pantherdb.org/genes/gene.do?acc=HUMAN%7CEnsembl=ENSG00000107937%7CUniProtKB=Q9BZE4)  [ortholog](http://www.pantherdb.org/genes/gene.do?acc=HUMAN%7CEnsembl=ENSG00000107937%7CUniProtKB=Q9BZE4#orthologs) | [translation](http://www.pantherdb.org/panther/category.do?categoryAcc=GO:0006412) |
| [HUMAN\|Ensembl=ENSG00000134597\|UniProtKB=Q9Y388](http://www.pantherdb.org/genes/gene.do?acc=HUMAN%7CEnsembl=ENSG00000134597%7CUniProtKB=Q9Y388) | Q9Y388 | RNA-binding motif protein, X-linked 2  [RBMX2](http://www.pantherdb.org/genes/gene.do?acc=HUMAN%7CEnsembl=ENSG00000134597%7CUniProtKB=Q9Y388)  [ortholog](http://www.pantherdb.org/genes/gene.do?acc=HUMAN%7CEnsembl=ENSG00000134597%7CUniProtKB=Q9Y388#orthologs) | [RNA splicing, via transesterification reactions](http://www.pantherdb.org/panther/category.do?categoryAcc=GO:0000375)  [mRNA splicing, via spliceosome](http://www.pantherdb.org/panther/category.do?categoryAcc=GO:0000398)  [RNA splicing, via transesterification reactions](http://www.pantherdb.org/panther/category.do?categoryAcc=GO:0000375) |
| [HUMAN\|Ensembl=ENSG00000130826\|UniProtKB=O60832](http://www.pantherdb.org/genes/gene.do?acc=HUMAN%7CEnsembl=ENSG00000130826%7CUniProtKB=O60832) | O60832 | H/ACA ribonucleoprotein complex subunit 4  [DKC1](http://www.pantherdb.org/genes/gene.do?acc=HUMAN%7CEnsembl=ENSG00000130826%7CUniProtKB=O60832)  [ortholog](http://www.pantherdb.org/genes/gene.do?acc=HUMAN%7CEnsembl=ENSG00000130826%7CUniProtKB=O60832#orthologs) | [rRNA metabolic process](http://www.pantherdb.org/panther/category.do?categoryAcc=GO:0016072)  [cell cycle](http://www.pantherdb.org/panther/category.do?categoryAcc=GO:0007049) |
| [HUMAN\|Ensembl=ENSG00000115524\|UniProtKB=O75533](http://www.pantherdb.org/genes/gene.do?acc=HUMAN%7CEnsembl=ENSG00000115524%7CUniProtKB=O75533) | O75533 | Splicing factor 3B subunit 1  [SF3B1](http://www.pantherdb.org/genes/gene.do?acc=HUMAN%7CEnsembl=ENSG00000115524%7CUniProtKB=O75533)  [ortholog](http://www.pantherdb.org/genes/gene.do?acc=HUMAN%7CEnsembl=ENSG00000115524%7CUniProtKB=O75533#orthologs) | [RNA splicing, via transesterification reactions](http://www.pantherdb.org/panther/category.do?categoryAcc=GO:0000375)  [mRNA splicing, via spliceosome](http://www.pantherdb.org/panther/category.do?categoryAcc=GO:0000398)  [RNA splicing, via transesterification reactions](http://www.pantherdb.org/panther/category.do?categoryAcc=GO:0000375) |
| [HUMAN\|Ensembl=ENSG00000101161\|UniProtKB=O94906](http://www.pantherdb.org/genes/gene.do?acc=HUMAN%7CEnsembl=ENSG00000101161%7CUniProtKB=O94906) | O94906 | Pre-mRNA-processing factor 6  [PRPF6](http://www.pantherdb.org/genes/gene.do?acc=HUMAN%7CEnsembl=ENSG00000101161%7CUniProtKB=O94906)  [ortholog](http://www.pantherdb.org/genes/gene.do?acc=HUMAN%7CEnsembl=ENSG00000101161%7CUniProtKB=O94906#orthologs) | [RNA splicing, via transesterification reactions](http://www.pantherdb.org/panther/category.do?categoryAcc=GO:0000375)  [mRNA splicing, via spliceosome](http://www.pantherdb.org/panther/category.do?categoryAcc=GO:0000398)  [RNA splicing, via transesterification reactions](http://www.pantherdb.org/panther/category.do?categoryAcc=GO:0000375)  [ectoderm development](http://www.pantherdb.org/panther/category.do?categoryAcc=GO:0007398)  [nervous system development](http://www.pantherdb.org/panther/category.do?categoryAcc=GO:0007399) |
| [HUMAN\|Ensembl=ENSG00000126267\|UniProtKB=P14854](http://www.pantherdb.org/genes/gene.do?acc=HUMAN%7CEnsembl=ENSG00000126267%7CUniProtKB=P14854) | P14854 | Cytochrome c oxidase subunit 6B1  [COX6B1](http://www.pantherdb.org/genes/gene.do?acc=HUMAN%7CEnsembl=ENSG00000126267%7CUniProtKB=P14854)  [ortholog](http://www.pantherdb.org/genes/gene.do?acc=HUMAN%7CEnsembl=ENSG00000126267%7CUniProtKB=P14854#orthologs) | [oxidative phosphorylation](http://www.pantherdb.org/panther/category.do?categoryAcc=GO:0006119)  [respiratory electron transport chain](http://www.pantherdb.org/panther/category.do?categoryAcc=GO:0022904) |
| [HUMAN\|Ensembl=ENSG00000173801\|UniProtKB=P14923](http://www.pantherdb.org/genes/gene.do?acc=HUMAN%7CEnsembl=ENSG00000173801%7CUniProtKB=P14923) | P14923 | Junction plakoglobin  [JUP](http://www.pantherdb.org/genes/gene.do?acc=HUMAN%7CEnsembl=ENSG00000173801%7CUniProtKB=P14923)  [ortholog](http://www.pantherdb.org/genes/gene.do?acc=HUMAN%7CEnsembl=ENSG00000173801%7CUniProtKB=P14923#orthologs) | [female gamete generation](http://www.pantherdb.org/panther/category.do?categoryAcc=GO:0007292)  [nitrogen compound metabolic process](http://www.pantherdb.org/panther/category.do?categoryAcc=GO:0006807)  [biosynthetic process](http://www.pantherdb.org/panther/category.do?categoryAcc=GO:0009058)  [transcription from RNA polymerase II promoter](http://www.pantherdb.org/panther/category.do?categoryAcc=GO:0006366)  [cell-cell signaling](http://www.pantherdb.org/panther/category.do?categoryAcc=GO:0007267)  [cell adhesion](http://www.pantherdb.org/panther/category.do?categoryAcc=GO:0007155)  [pattern specification process](http://www.pantherdb.org/panther/category.do?categoryAcc=GO:0007389)  [pattern specification process](http://www.pantherdb.org/panther/category.do?categoryAcc=GO:0007389)  [cellular component morphogenesis](http://www.pantherdb.org/panther/category.do?categoryAcc=GO:0032989)  [embryo development](http://www.pantherdb.org/panther/category.do?categoryAcc=GO:0009790)  [cell differentiation](http://www.pantherdb.org/panther/category.do?categoryAcc=GO:0030154)  [heart development](http://www.pantherdb.org/panther/category.do?categoryAcc=GO:0007507)  [response to stimulus](http://www.pantherdb.org/panther/category.do?categoryAcc=GO:0050896)  [protein localization](http://www.pantherdb.org/panther/category.do?categoryAcc=GO:0008104)  [regulation of transcription from RNA polymerase II promoter](http://www.pantherdb.org/panther/category.do?categoryAcc=GO:0006357)  [cytoskeleton organization](http://www.pantherdb.org/panther/category.do?categoryAcc=GO:0007010) |
| [HUMAN\|Ensembl=ENSG00000105202\|UniProtKB=P22087](http://www.pantherdb.org/genes/gene.do?acc=HUMAN%7CEnsembl=ENSG00000105202%7CUniProtKB=P22087) | P22087 | rRNA 2'-O-methyltransferase fibrillarin  [FBL](http://www.pantherdb.org/genes/gene.do?acc=HUMAN%7CEnsembl=ENSG00000105202%7CUniProtKB=P22087)  [ortholog](http://www.pantherdb.org/genes/gene.do?acc=HUMAN%7CEnsembl=ENSG00000105202%7CUniProtKB=P22087#orthologs) | [rRNA metabolic process](http://www.pantherdb.org/panther/category.do?categoryAcc=GO:0016072) |
| [HUMAN\|Ensembl=ENSG00000261236\|UniProtKB=Q14137](http://www.pantherdb.org/genes/gene.do?acc=HUMAN%7CEnsembl=ENSG00000261236%7CUniProtKB=Q14137) | Q14137 | Ribosome biogenesis protein BOP1  [BOP1](http://www.pantherdb.org/genes/gene.do?acc=HUMAN%7CEnsembl=ENSG00000261236%7CUniProtKB=Q14137)  [ortholog](http://www.pantherdb.org/genes/gene.do?acc=HUMAN%7CEnsembl=ENSG00000261236%7CUniProtKB=Q14137#orthologs) | [rRNA metabolic process](http://www.pantherdb.org/panther/category.do?categoryAcc=GO:0016072)  [translation](http://www.pantherdb.org/panther/category.do?categoryAcc=GO:0006412) |
| [HUMAN\|Ensembl=ENSG00000007968\|UniProtKB=Q14209](http://www.pantherdb.org/genes/gene.do?acc=HUMAN%7CEnsembl=ENSG00000007968%7CUniProtKB=Q14209) | Q14209 | Transcription factor E2F2  [E2F2](http://www.pantherdb.org/genes/gene.do?acc=HUMAN%7CEnsembl=ENSG00000007968%7CUniProtKB=Q14209)  [ortholog](http://www.pantherdb.org/genes/gene.do?acc=HUMAN%7CEnsembl=ENSG00000007968%7CUniProtKB=Q14209#orthologs) | [transcription from RNA polymerase II promoter](http://www.pantherdb.org/panther/category.do?categoryAcc=GO:0006366)  [cell cycle](http://www.pantherdb.org/panther/category.do?categoryAcc=GO:0007049)  [cell communication](http://www.pantherdb.org/panther/category.do?categoryAcc=GO:0007154)  [regulation of transcription from RNA polymerase II promoter](http://www.pantherdb.org/panther/category.do?categoryAcc=GO:0006357) |
| [HUMAN\|Ensembl=ENSG00000174231\|UniProtKB=Q6P2Q9](http://www.pantherdb.org/genes/gene.do?acc=HUMAN%7CEnsembl=ENSG00000174231%7CUniProtKB=Q6P2Q9) | Q6P2Q9 | Pre-mRNA-processing-splicing factor 8  [PRPF8](http://www.pantherdb.org/genes/gene.do?acc=HUMAN%7CEnsembl=ENSG00000174231%7CUniProtKB=Q6P2Q9)  [ortholog](http://www.pantherdb.org/genes/gene.do?acc=HUMAN%7CEnsembl=ENSG00000174231%7CUniProtKB=Q6P2Q9#orthologs) | [RNA splicing, via transesterification reactions](http://www.pantherdb.org/panther/category.do?categoryAcc=GO:0000375)  [mRNA splicing, via spliceosome](http://www.pantherdb.org/panther/category.do?categoryAcc=GO:0000398)  [RNA splicing, via transesterification reactions](http://www.pantherdb.org/panther/category.do?categoryAcc=GO:0000375) |
| [HUMAN\|Ensembl=ENSG00000134987\|UniProtKB=Q8NI36](http://www.pantherdb.org/genes/gene.do?acc=HUMAN%7CEnsembl=ENSG00000134987%7CUniProtKB=Q8NI36) | Q8NI36 | WD repeat-containing protein 36  [WDR36](http://www.pantherdb.org/genes/gene.do?acc=HUMAN%7CEnsembl=ENSG00000134987%7CUniProtKB=Q8NI36)  [ortholog](http://www.pantherdb.org/genes/gene.do?acc=HUMAN%7CEnsembl=ENSG00000134987%7CUniProtKB=Q8NI36#orthologs) | [RNA splicing, via transesterification reactions](http://www.pantherdb.org/panther/category.do?categoryAcc=GO:0000375)  [mRNA splicing, via spliceosome](http://www.pantherdb.org/panther/category.do?categoryAcc=GO:0000398)  [RNA splicing, via transesterification reactions](http://www.pantherdb.org/panther/category.do?categoryAcc=GO:0000375) |
| [HUMAN\|Ensembl=ENSG00000048649\|UniProtKB=Q96T23](http://www.pantherdb.org/genes/gene.do?acc=HUMAN%7CEnsembl=ENSG00000048649%7CUniProtKB=Q96T23) | Q96T23 | Remodeling and spacing factor 1  [RSF1](http://www.pantherdb.org/genes/gene.do?acc=HUMAN%7CEnsembl=ENSG00000048649%7CUniProtKB=Q96T23)  [ortholog](http://www.pantherdb.org/genes/gene.do?acc=HUMAN%7CEnsembl=ENSG00000048649%7CUniProtKB=Q96T23#orthologs) | [induction of apoptosis](http://www.pantherdb.org/panther/category.do?categoryAcc=GO:0006917)  [transcription from RNA polymerase II promoter](http://www.pantherdb.org/panther/category.do?categoryAcc=GO:0006366)  [protein acetylation](http://www.pantherdb.org/panther/category.do?categoryAcc=GO:0006473)  [cell cycle](http://www.pantherdb.org/panther/category.do?categoryAcc=GO:0007049)  [induction of apoptosis](http://www.pantherdb.org/panther/category.do?categoryAcc=GO:0006917)  [regulation of transcription from RNA polymerase II promoter](http://www.pantherdb.org/panther/category.do?categoryAcc=GO:0006357) |
| [HUMAN\|Ensembl=ENSG00000185129\|UniProtKB=Q00577](http://www.pantherdb.org/genes/gene.do?acc=HUMAN%7CEnsembl=ENSG00000185129%7CUniProtKB=Q00577) | Q00577 | Transcriptional activator protein Pur-alpha  [PURA](http://www.pantherdb.org/genes/gene.do?acc=HUMAN%7CEnsembl=ENSG00000185129%7CUniProtKB=Q00577)  [ortholog](http://www.pantherdb.org/genes/gene.do?acc=HUMAN%7CEnsembl=ENSG00000185129%7CUniProtKB=Q00577#orthologs) | [transcription from RNA polymerase II promoter](http://www.pantherdb.org/panther/category.do?categoryAcc=GO:0006366) |
| [HUMAN\|Ensembl=ENSG00000135829\|UniProtKB=Q08211](http://www.pantherdb.org/genes/gene.do?acc=HUMAN%7CEnsembl=ENSG00000135829%7CUniProtKB=Q08211) | Q08211 | ATP-dependent RNA helicase A  [DHX9](http://www.pantherdb.org/genes/gene.do?acc=HUMAN%7CEnsembl=ENSG00000135829%7CUniProtKB=Q08211)  [ortholog](http://www.pantherdb.org/genes/gene.do?acc=HUMAN%7CEnsembl=ENSG00000135829%7CUniProtKB=Q08211#orthologs) | [mRNA splicing, via spliceosome](http://www.pantherdb.org/panther/category.do?categoryAcc=GO:0000398) |
| [HUMAN\|Ensembl=ENSG00000112739\|UniProtKB=Q13523](http://www.pantherdb.org/genes/gene.do?acc=HUMAN%7CEnsembl=ENSG00000112739%7CUniProtKB=Q13523) | Q13523 | Serine/threonine-protein kinase PRP4 homolog  [PRPF4B](http://www.pantherdb.org/genes/gene.do?acc=HUMAN%7CEnsembl=ENSG00000112739%7CUniProtKB=Q13523)  [ortholog](http://www.pantherdb.org/genes/gene.do?acc=HUMAN%7CEnsembl=ENSG00000112739%7CUniProtKB=Q13523#orthologs) | [glycogen metabolic process](http://www.pantherdb.org/panther/category.do?categoryAcc=GO:0005977)  [protein phosphorylation](http://www.pantherdb.org/panther/category.do?categoryAcc=GO:0006468)  [mitosis](http://www.pantherdb.org/panther/category.do?categoryAcc=GO:0007067)  [cell communication](http://www.pantherdb.org/panther/category.do?categoryAcc=GO:0007154) |
| [HUMAN\|Ensembl=ENSG00000165733\|UniProtKB=Q14692](http://www.pantherdb.org/genes/gene.do?acc=HUMAN%7CEnsembl=ENSG00000165733%7CUniProtKB=Q14692) | Q14692 | Ribosome biogenesis protein BMS1 homolog  [BMS1](http://www.pantherdb.org/genes/gene.do?acc=HUMAN%7CEnsembl=ENSG00000165733%7CUniProtKB=Q14692)  [ortholog](http://www.pantherdb.org/genes/gene.do?acc=HUMAN%7CEnsembl=ENSG00000165733%7CUniProtKB=Q14692#orthologs) | [nucleobase-containing compound metabolic process](http://www.pantherdb.org/panther/category.do?categoryAcc=GO:0006139) |
| [HUMAN\|Ensembl=ENSG00000115368\|UniProtKB=Q8IWA0](http://www.pantherdb.org/genes/gene.do?acc=HUMAN%7CEnsembl=ENSG00000115368%7CUniProtKB=Q8IWA0) | Q8IWA0 | WD repeat-containing protein 75  [WDR75](http://www.pantherdb.org/genes/gene.do?acc=HUMAN%7CEnsembl=ENSG00000115368%7CUniProtKB=Q8IWA0)  [ortholog](http://www.pantherdb.org/genes/gene.do?acc=HUMAN%7CEnsembl=ENSG00000115368%7CUniProtKB=Q8IWA0#orthologs) | [RNA splicing, via transesterification reactions](http://www.pantherdb.org/panther/category.do?categoryAcc=GO:0000375)  [transcription from RNA polymerase II promoter](http://www.pantherdb.org/panther/category.do?categoryAcc=GO:0006366)  [mRNA splicing, via spliceosome](http://www.pantherdb.org/panther/category.do?categoryAcc=GO:0000398)  [RNA splicing, via transesterification reactions](http://www.pantherdb.org/panther/category.do?categoryAcc=GO:0000375)  [RNA localization](http://www.pantherdb.org/panther/category.do?categoryAcc=GO:0006403)  [regulation of catalytic activity](http://www.pantherdb.org/panther/category.do?categoryAcc=GO:0050790) |
| [HUMAN\|Ensembl=ENSG00000096401\|UniProtKB=Q99459](http://www.pantherdb.org/genes/gene.do?acc=HUMAN%7CEnsembl=ENSG00000096401%7CUniProtKB=Q99459) | Q99459 | Cell division cycle 5-like protein  [CDC5L](http://www.pantherdb.org/genes/gene.do?acc=HUMAN%7CEnsembl=ENSG00000096401%7CUniProtKB=Q99459)  [ortholog](http://www.pantherdb.org/genes/gene.do?acc=HUMAN%7CEnsembl=ENSG00000096401%7CUniProtKB=Q99459#orthologs) | [purine nucleobase metabolic process](http://www.pantherdb.org/panther/category.do?categoryAcc=GO:0006144)  [transcription from RNA polymerase II promoter](http://www.pantherdb.org/panther/category.do?categoryAcc=GO:0006366)  [rRNA metabolic process](http://www.pantherdb.org/panther/category.do?categoryAcc=GO:0016072)  [cellular amino acid biosynthetic process](http://www.pantherdb.org/panther/category.do?categoryAcc=GO:0008652)  [cell cycle](http://www.pantherdb.org/panther/category.do?categoryAcc=GO:0007049)  [regulation of transcription from RNA polymerase II promoter](http://www.pantherdb.org/panther/category.do?categoryAcc=GO:0006357) |
| [HUMAN\|Ensembl=ENSG00000130810\|UniProtKB=Q9NQ55](http://www.pantherdb.org/genes/gene.do?acc=HUMAN%7CEnsembl=ENSG00000130810%7CUniProtKB=Q9NQ55) | Q9NQ55 | Suppressor of SWI4 1 homolog  [PPAN](http://www.pantherdb.org/genes/gene.do?acc=HUMAN%7CEnsembl=ENSG00000130810%7CUniProtKB=Q9NQ55)  [ortholog](http://www.pantherdb.org/genes/gene.do?acc=HUMAN%7CEnsembl=ENSG00000130810%7CUniProtKB=Q9NQ55#orthologs) | [mRNA splicing, via spliceosome](http://www.pantherdb.org/panther/category.do?categoryAcc=GO:0000398) |
| [HUMAN\|Ensembl=ENSG00000053372\|UniProtKB=Q9UKD2](http://www.pantherdb.org/genes/gene.do?acc=HUMAN%7CEnsembl=ENSG00000053372%7CUniProtKB=Q9UKD2) | Q9UKD2 | mRNA turnover protein 4 homolog  [MRTO4](http://www.pantherdb.org/genes/gene.do?acc=HUMAN%7CEnsembl=ENSG00000053372%7CUniProtKB=Q9UKD2)  [ortholog](http://www.pantherdb.org/genes/gene.do?acc=HUMAN%7CEnsembl=ENSG00000053372%7CUniProtKB=Q9UKD2#orthologs) | [translation](http://www.pantherdb.org/panther/category.do?categoryAcc=GO:0006412) |
| [HUMAN\|Ensembl=ENSG00000065183\|UniProtKB=Q9UNX4](http://www.pantherdb.org/genes/gene.do?acc=HUMAN%7CEnsembl=ENSG00000065183%7CUniProtKB=Q9UNX4) | Q9UNX4 | WD repeat-containing protein 3  [WDR3](http://www.pantherdb.org/genes/gene.do?acc=HUMAN%7CEnsembl=ENSG00000065183%7CUniProtKB=Q9UNX4)  [ortholog](http://www.pantherdb.org/genes/gene.do?acc=HUMAN%7CEnsembl=ENSG00000065183%7CUniProtKB=Q9UNX4#orthologs) | [nucleobase-containing compound metabolic process](http://www.pantherdb.org/panther/category.do?categoryAcc=GO:0006139) |
| [HUMAN\|Ensembl=ENSG00000054118\|UniProtKB=Q9Y2W1](http://www.pantherdb.org/genes/gene.do?acc=HUMAN%7CEnsembl=ENSG00000054118%7CUniProtKB=Q9Y2W1) | Q9Y2W1 | Thyroid hormone receptor-associated protein 3  [THRAP3](http://www.pantherdb.org/genes/gene.do?acc=HUMAN%7CEnsembl=ENSG00000054118%7CUniProtKB=Q9Y2W1)  [ortholog](http://www.pantherdb.org/genes/gene.do?acc=HUMAN%7CEnsembl=ENSG00000054118%7CUniProtKB=Q9Y2W1#orthologs) | [transcription from RNA polymerase II promoter](http://www.pantherdb.org/panther/category.do?categoryAcc=GO:0006366)  [regulation of transcription from RNA polymerase II promoter](http://www.pantherdb.org/panther/category.do?categoryAcc=GO:0006357) |
| [HUMAN\|Ensembl=ENSG00000055044\|UniProtKB=Q9Y2X3](http://www.pantherdb.org/genes/gene.do?acc=HUMAN%7CEnsembl=ENSG00000055044%7CUniProtKB=Q9Y2X3) | Q9Y2X3 | Nucleolar protein 58  [NOP58](http://www.pantherdb.org/genes/gene.do?acc=HUMAN%7CEnsembl=ENSG00000055044%7CUniProtKB=Q9Y2X3)  [ortholog](http://www.pantherdb.org/genes/gene.do?acc=HUMAN%7CEnsembl=ENSG00000055044%7CUniProtKB=Q9Y2X3#orthologs) | [rRNA metabolic process](http://www.pantherdb.org/panther/category.do?categoryAcc=GO:0016072) |
| [HUMAN\|Ensembl=ENSG00000168298\|UniProtKB=P10412](http://www.pantherdb.org/genes/gene.do?acc=HUMAN%7CEnsembl=ENSG00000168298%7CUniProtKB=P10412) | P10412 | Histone H1.4  [HIST1H1E](http://www.pantherdb.org/genes/gene.do?acc=HUMAN%7CEnsembl=ENSG00000168298%7CUniProtKB=P10412)  [ortholog](http://www.pantherdb.org/genes/gene.do?acc=HUMAN%7CEnsembl=ENSG00000168298%7CUniProtKB=P10412#orthologs) | [nucleobase-containing compound metabolic process](http://www.pantherdb.org/panther/category.do?categoryAcc=GO:0006139)  [cellular process](http://www.pantherdb.org/panther/category.do?categoryAcc=GO:0009987)  [chromatin organization](http://www.pantherdb.org/panther/category.do?categoryAcc=GO:0006325) |
| [HUMAN\|Ensembl=ENSG00000168036\|UniProtKB=P35222](http://www.pantherdb.org/genes/gene.do?acc=HUMAN%7CEnsembl=ENSG00000168036%7CUniProtKB=P35222) | P35222 | Catenin beta-1  [CTNNB1](http://www.pantherdb.org/genes/gene.do?acc=HUMAN%7CEnsembl=ENSG00000168036%7CUniProtKB=P35222)  [ortholog](http://www.pantherdb.org/genes/gene.do?acc=HUMAN%7CEnsembl=ENSG00000168036%7CUniProtKB=P35222#orthologs) | [female gamete generation](http://www.pantherdb.org/panther/category.do?categoryAcc=GO:0007292)  [immune system process](http://www.pantherdb.org/panther/category.do?categoryAcc=GO:0002376)  [apoptotic process](http://www.pantherdb.org/panther/category.do?categoryAcc=GO:0006915)  [nitrogen compound metabolic process](http://www.pantherdb.org/panther/category.do?categoryAcc=GO:0006807)  [biosynthetic process](http://www.pantherdb.org/panther/category.do?categoryAcc=GO:0009058)  [transcription from RNA polymerase II promoter](http://www.pantherdb.org/panther/category.do?categoryAcc=GO:0006366)  [synaptic transmission](http://www.pantherdb.org/panther/category.do?categoryAcc=GO:0007268)  [cell proliferation](http://www.pantherdb.org/panther/category.do?categoryAcc=GO:0008283)  [cell adhesion](http://www.pantherdb.org/panther/category.do?categoryAcc=GO:0007155)  [neurological system process](http://www.pantherdb.org/panther/category.do?categoryAcc=GO:0050877)  [pattern specification process](http://www.pantherdb.org/panther/category.do?categoryAcc=GO:0007389)  [endoderm development](http://www.pantherdb.org/panther/category.do?categoryAcc=GO:0007492)  [cellular component morphogenesis](http://www.pantherdb.org/panther/category.do?categoryAcc=GO:0032989)  [embryo development](http://www.pantherdb.org/panther/category.do?categoryAcc=GO:0009790)  [apoptotic process](http://www.pantherdb.org/panther/category.do?categoryAcc=GO:0006915)  [cell differentiation](http://www.pantherdb.org/panther/category.do?categoryAcc=GO:0030154)  [skeletal system development](http://www.pantherdb.org/panther/category.do?categoryAcc=GO:0001501)  [angiogenesis](http://www.pantherdb.org/panther/category.do?categoryAcc=GO:0001525)  [nervous system development](http://www.pantherdb.org/panther/category.do?categoryAcc=GO:0007399)  [heart development](http://www.pantherdb.org/panther/category.do?categoryAcc=GO:0007507)  [hemopoiesis](http://www.pantherdb.org/panther/category.do?categoryAcc=GO:0030097)  [response to endogenous stimulus](http://www.pantherdb.org/panther/category.do?categoryAcc=GO:0009719)  [vesicle-mediated transport](http://www.pantherdb.org/panther/category.do?categoryAcc=GO:0016192)  [protein localization](http://www.pantherdb.org/panther/category.do?categoryAcc=GO:0008104)  [regulation of transcription from RNA polymerase II promoter](http://www.pantherdb.org/panther/category.do?categoryAcc=GO:0006357)  [regulation of sequence-specific DNA binding transcription factor activity](http://www.pantherdb.org/panther/category.do?categoryAcc=GO:0051090)  [cytoskeleton organization](http://www.pantherdb.org/panther/category.do?categoryAcc=GO:0007010) |
| [HUMAN\|Ensembl=ENSG00000253729\|UniProtKB=P78527](http://www.pantherdb.org/genes/gene.do?acc=HUMAN%7CEnsembl=ENSG00000253729%7CUniProtKB=P78527) | P78527 | DNA-dependent protein kinase catalytic subunit  [PRKDC](http://www.pantherdb.org/genes/gene.do?acc=HUMAN%7CEnsembl=ENSG00000253729%7CUniProtKB=P78527)  [ortholog](http://www.pantherdb.org/genes/gene.do?acc=HUMAN%7CEnsembl=ENSG00000253729%7CUniProtKB=P78527#orthologs) | [immune system process](http://www.pantherdb.org/panther/category.do?categoryAcc=GO:0002376)  [induction of apoptosis](http://www.pantherdb.org/panther/category.do?categoryAcc=GO:0006917)  [DNA repair](http://www.pantherdb.org/panther/category.do?categoryAcc=GO:0006281)  [DNA recombination](http://www.pantherdb.org/panther/category.do?categoryAcc=GO:0006310)  [protein phosphorylation](http://www.pantherdb.org/panther/category.do?categoryAcc=GO:0006468)  [cell cycle](http://www.pantherdb.org/panther/category.do?categoryAcc=GO:0007049)  [cell communication](http://www.pantherdb.org/panther/category.do?categoryAcc=GO:0007154)  [induction of apoptosis](http://www.pantherdb.org/panther/category.do?categoryAcc=GO:0006917)  [response to stress](http://www.pantherdb.org/panther/category.do?categoryAcc=GO:0006950)  [chromatin organization](http://www.pantherdb.org/panther/category.do?categoryAcc=GO:0006325) |
| [HUMAN\|Ensembl=ENSG00000131051\|UniProtKB=Q14498](http://www.pantherdb.org/genes/gene.do?acc=HUMAN%7CEnsembl=ENSG00000131051%7CUniProtKB=Q14498) | Q14498 | RNA-binding protein 39  [RBM39](http://www.pantherdb.org/genes/gene.do?acc=HUMAN%7CEnsembl=ENSG00000131051%7CUniProtKB=Q14498)  [ortholog](http://www.pantherdb.org/genes/gene.do?acc=HUMAN%7CEnsembl=ENSG00000131051%7CUniProtKB=Q14498#orthologs) | [DNA replication](http://www.pantherdb.org/panther/category.do?categoryAcc=GO:0006260)  [RNA splicing, via transesterification reactions](http://www.pantherdb.org/panther/category.do?categoryAcc=GO:0000375)  [mRNA splicing, via spliceosome](http://www.pantherdb.org/panther/category.do?categoryAcc=GO:0000398)  [mRNA polyadenylation](http://www.pantherdb.org/panther/category.do?categoryAcc=GO:0006378)  [RNA splicing, via transesterification reactions](http://www.pantherdb.org/panther/category.do?categoryAcc=GO:0000375)  [rRNA metabolic process](http://www.pantherdb.org/panther/category.do?categoryAcc=GO:0016072)  [protein metabolic process](http://www.pantherdb.org/panther/category.do?categoryAcc=GO:0019538)  [cell cycle](http://www.pantherdb.org/panther/category.do?categoryAcc=GO:0007049)  [neurological system process](http://www.pantherdb.org/panther/category.do?categoryAcc=GO:0050877)  [ectoderm development](http://www.pantherdb.org/panther/category.do?categoryAcc=GO:0007398)  [nervous system development](http://www.pantherdb.org/panther/category.do?categoryAcc=GO:0007399) |
| [HUMAN\|Ensembl=ENSG00000108592\|UniProtKB=Q8IY81](http://www.pantherdb.org/genes/gene.do?acc=HUMAN%7CEnsembl=ENSG00000108592%7CUniProtKB=Q8IY81) | Q8IY81 | pre-rRNA processing protein FTSJ3  [FTSJ3](http://www.pantherdb.org/genes/gene.do?acc=HUMAN%7CEnsembl=ENSG00000108592%7CUniProtKB=Q8IY81)  [ortholog](http://www.pantherdb.org/genes/gene.do?acc=HUMAN%7CEnsembl=ENSG00000108592%7CUniProtKB=Q8IY81#orthologs) | [rRNA metabolic process](http://www.pantherdb.org/panther/category.do?categoryAcc=GO:0016072) |
| [HUMAN\|Ensembl=ENSG00000188976\|UniProtKB=Q9Y3T9](http://www.pantherdb.org/genes/gene.do?acc=HUMAN%7CEnsembl=ENSG00000188976%7CUniProtKB=Q9Y3T9) | Q9Y3T9 | Nucleolar complex protein 2 homolog  [NOC2L](http://www.pantherdb.org/genes/gene.do?acc=HUMAN%7CEnsembl=ENSG00000188976%7CUniProtKB=Q9Y3T9)  [ortholog](http://www.pantherdb.org/genes/gene.do?acc=HUMAN%7CEnsembl=ENSG00000188976%7CUniProtKB=Q9Y3T9#orthologs) | [protein metabolic process](http://www.pantherdb.org/panther/category.do?categoryAcc=GO:0019538)  [protein targeting](http://www.pantherdb.org/panther/category.do?categoryAcc=GO:0006605)  [nuclear transport](http://www.pantherdb.org/panther/category.do?categoryAcc=GO:0051169) |
| [HUMAN\|Ensembl=ENSG00000172809\|UniProtKB=P63173](http://www.pantherdb.org/genes/gene.do?acc=HUMAN%7CEnsembl=ENSG00000172809%7CUniProtKB=P63173) | P63173 | 60S ribosomal protein L38  [RPL38](http://www.pantherdb.org/genes/gene.do?acc=HUMAN%7CEnsembl=ENSG00000172809%7CUniProtKB=P63173)  [ortholog](http://www.pantherdb.org/genes/gene.do?acc=HUMAN%7CEnsembl=ENSG00000172809%7CUniProtKB=P63173#orthologs) | [translation](http://www.pantherdb.org/panther/category.do?categoryAcc=GO:0006412) |
| [HUMAN\|Ensembl=ENSG00000115816\|UniProtKB=Q03701](http://www.pantherdb.org/genes/gene.do?acc=HUMAN%7CEnsembl=ENSG00000115816%7CUniProtKB=Q03701) | Q03701 | CCAAT/enhancer-binding protein zeta  [CEBPZ](http://www.pantherdb.org/genes/gene.do?acc=HUMAN%7CEnsembl=ENSG00000115816%7CUniProtKB=Q03701)  [ortholog](http://www.pantherdb.org/genes/gene.do?acc=HUMAN%7CEnsembl=ENSG00000115816%7CUniProtKB=Q03701#orthologs) | [transcription from RNA polymerase II promoter](http://www.pantherdb.org/panther/category.do?categoryAcc=GO:0006366)  [regulation of transcription from RNA polymerase II promoter](http://www.pantherdb.org/panther/category.do?categoryAcc=GO:0006357) |
| [HUMAN\|Ensembl=ENSG00000111530\|UniProtKB=Q86VP6](http://www.pantherdb.org/genes/gene.do?acc=HUMAN%7CEnsembl=ENSG00000111530%7CUniProtKB=Q86VP6) | Q86VP6 | Cullin-associated NEDD8-dissociated protein 1  [CAND1](http://www.pantherdb.org/genes/gene.do?acc=HUMAN%7CEnsembl=ENSG00000111530%7CUniProtKB=Q86VP6)  [ortholog](http://www.pantherdb.org/genes/gene.do?acc=HUMAN%7CEnsembl=ENSG00000111530%7CUniProtKB=Q86VP6#orthologs) | [transcription from RNA polymerase II promoter](http://www.pantherdb.org/panther/category.do?categoryAcc=GO:0006366) |
| [HUMAN\|Ensembl=ENSG00000123064\|UniProtKB=Q8TDD1](http://www.pantherdb.org/genes/gene.do?acc=HUMAN%7CEnsembl=ENSG00000123064%7CUniProtKB=Q8TDD1) | Q8TDD1 | ATP-dependent RNA helicase DDX54  [DDX54](http://www.pantherdb.org/genes/gene.do?acc=HUMAN%7CEnsembl=ENSG00000123064%7CUniProtKB=Q8TDD1)  [ortholog](http://www.pantherdb.org/genes/gene.do?acc=HUMAN%7CEnsembl=ENSG00000123064%7CUniProtKB=Q8TDD1#orthologs) | [nucleobase-containing compound metabolic process](http://www.pantherdb.org/panther/category.do?categoryAcc=GO:0006139)  [translation](http://www.pantherdb.org/panther/category.do?categoryAcc=GO:0006412)  [regulation of translation](http://www.pantherdb.org/panther/category.do?categoryAcc=GO:0006417) |
| [HUMAN\|Ensembl=ENSG00000156697\|UniProtKB=Q9BVJ6](http://www.pantherdb.org/genes/gene.do?acc=HUMAN%7CEnsembl=ENSG00000156697%7CUniProtKB=Q9BVJ6) | Q9BVJ6 | U3 small nucleolar RNA-associated protein 14 homolog A  [UTP14A](http://www.pantherdb.org/genes/gene.do?acc=HUMAN%7CEnsembl=ENSG00000156697%7CUniProtKB=Q9BVJ6)  [ortholog](http://www.pantherdb.org/genes/gene.do?acc=HUMAN%7CEnsembl=ENSG00000156697%7CUniProtKB=Q9BVJ6#orthologs) | [nucleobase-containing compound metabolic process](http://www.pantherdb.org/panther/category.do?categoryAcc=GO:0006139) |
| [HUMAN\|Ensembl=ENSG00000089737\|UniProtKB=Q9GZR7](http://www.pantherdb.org/genes/gene.do?acc=HUMAN%7CEnsembl=ENSG00000089737%7CUniProtKB=Q9GZR7) | Q9GZR7 | ATP-dependent RNA helicase DDX24  [DDX24](http://www.pantherdb.org/genes/gene.do?acc=HUMAN%7CEnsembl=ENSG00000089737%7CUniProtKB=Q9GZR7)  [ortholog](http://www.pantherdb.org/genes/gene.do?acc=HUMAN%7CEnsembl=ENSG00000089737%7CUniProtKB=Q9GZR7#orthologs) | [nucleobase-containing compound metabolic process](http://www.pantherdb.org/panther/category.do?categoryAcc=GO:0006139)  [translation](http://www.pantherdb.org/panther/category.do?categoryAcc=GO:0006412)  [regulation of translation](http://www.pantherdb.org/panther/category.do?categoryAcc=GO:0006417) |
| [HUMAN\|Ensembl=ENSG00000122566\|UniProtKB=P22626](http://www.pantherdb.org/genes/gene.do?acc=HUMAN%7CEnsembl=ENSG00000122566%7CUniProtKB=P22626) | P22626 | Heterogeneous nuclear ribonucleoproteins A2/B1  [HNRNPA2B1](http://www.pantherdb.org/genes/gene.do?acc=HUMAN%7CEnsembl=ENSG00000122566%7CUniProtKB=P22626)  [ortholog](http://www.pantherdb.org/genes/gene.do?acc=HUMAN%7CEnsembl=ENSG00000122566%7CUniProtKB=P22626#orthologs) | [DNA replication](http://www.pantherdb.org/panther/category.do?categoryAcc=GO:0006260)  [RNA splicing, via transesterification reactions](http://www.pantherdb.org/panther/category.do?categoryAcc=GO:0000375)  [mRNA splicing, via spliceosome](http://www.pantherdb.org/panther/category.do?categoryAcc=GO:0000398)  [mRNA polyadenylation](http://www.pantherdb.org/panther/category.do?categoryAcc=GO:0006378)  [RNA splicing, via transesterification reactions](http://www.pantherdb.org/panther/category.do?categoryAcc=GO:0000375)  [rRNA metabolic process](http://www.pantherdb.org/panther/category.do?categoryAcc=GO:0016072)  [protein metabolic process](http://www.pantherdb.org/panther/category.do?categoryAcc=GO:0019538)  [cell cycle](http://www.pantherdb.org/panther/category.do?categoryAcc=GO:0007049)  [neurological system process](http://www.pantherdb.org/panther/category.do?categoryAcc=GO:0050877)  [ectoderm development](http://www.pantherdb.org/panther/category.do?categoryAcc=GO:0007398)  [nervous system development](http://www.pantherdb.org/panther/category.do?categoryAcc=GO:0007399) |
| [HUMAN\|Ensembl=ENSG00000067066\|UniProtKB=P23497](http://www.pantherdb.org/genes/gene.do?acc=HUMAN%7CEnsembl=ENSG00000067066%7CUniProtKB=P23497) | P23497 | Nuclear autoantigen Sp-100  [SP100](http://www.pantherdb.org/genes/gene.do?acc=HUMAN%7CEnsembl=ENSG00000067066%7CUniProtKB=P23497)  [ortholog](http://www.pantherdb.org/genes/gene.do?acc=HUMAN%7CEnsembl=ENSG00000067066%7CUniProtKB=P23497#orthologs) | [transcription from RNA polymerase II promoter](http://www.pantherdb.org/panther/category.do?categoryAcc=GO:0006366)  [cell communication](http://www.pantherdb.org/panther/category.do?categoryAcc=GO:0007154)  [regulation of transcription from RNA polymerase II promoter](http://www.pantherdb.org/panther/category.do?categoryAcc=GO:0006357)  [chromatin organization](http://www.pantherdb.org/panther/category.do?categoryAcc=GO:0006325) |
| [HUMAN\|Ensembl=ENSG00000121774\|UniProtKB=Q07666](http://www.pantherdb.org/genes/gene.do?acc=HUMAN%7CEnsembl=ENSG00000121774%7CUniProtKB=Q07666) | Q07666 | KH domain-containing, RNA-binding, signal transduction-associated protein 1  [KHDRBS1](http://www.pantherdb.org/genes/gene.do?acc=HUMAN%7CEnsembl=ENSG00000121774%7CUniProtKB=Q07666)  [ortholog](http://www.pantherdb.org/genes/gene.do?acc=HUMAN%7CEnsembl=ENSG00000121774%7CUniProtKB=Q07666#orthologs) | [spermatogenesis](http://www.pantherdb.org/panther/category.do?categoryAcc=GO:0007283)  [RNA splicing, via transesterification reactions](http://www.pantherdb.org/panther/category.do?categoryAcc=GO:0000375)  [mRNA splicing, via spliceosome](http://www.pantherdb.org/panther/category.do?categoryAcc=GO:0000398) |
| [HUMAN\|Ensembl=ENSG00000179041\|UniProtKB=Q15050](http://www.pantherdb.org/genes/gene.do?acc=HUMAN%7CEnsembl=ENSG00000179041%7CUniProtKB=Q15050) | Q15050 | Ribosome biogenesis regulatory protein homolog  [RRS1](http://www.pantherdb.org/genes/gene.do?acc=HUMAN%7CEnsembl=ENSG00000179041%7CUniProtKB=Q15050)  [ortholog](http://www.pantherdb.org/genes/gene.do?acc=HUMAN%7CEnsembl=ENSG00000179041%7CUniProtKB=Q15050#orthologs) | [RNA splicing, via transesterification reactions](http://www.pantherdb.org/panther/category.do?categoryAcc=GO:0000375)  [nucleobase-containing compound metabolic process](http://www.pantherdb.org/panther/category.do?categoryAcc=GO:0006139) |
| [HUMAN\|Ensembl=ENSG00000163811\|UniProtKB=Q15061](http://www.pantherdb.org/genes/gene.do?acc=HUMAN%7CEnsembl=ENSG00000163811%7CUniProtKB=Q15061) | Q15061 | WD repeat-containing protein 43  [WDR43](http://www.pantherdb.org/genes/gene.do?acc=HUMAN%7CEnsembl=ENSG00000163811%7CUniProtKB=Q15061)  [ortholog](http://www.pantherdb.org/genes/gene.do?acc=HUMAN%7CEnsembl=ENSG00000163811%7CUniProtKB=Q15061#orthologs) | [RNA splicing, via transesterification reactions](http://www.pantherdb.org/panther/category.do?categoryAcc=GO:0000375)  [transcription from RNA polymerase II promoter](http://www.pantherdb.org/panther/category.do?categoryAcc=GO:0006366)  [mRNA splicing, via spliceosome](http://www.pantherdb.org/panther/category.do?categoryAcc=GO:0000398)  [RNA splicing, via transesterification reactions](http://www.pantherdb.org/panther/category.do?categoryAcc=GO:0000375)  [ectoderm development](http://www.pantherdb.org/panther/category.do?categoryAcc=GO:0007398)  [RNA localization](http://www.pantherdb.org/panther/category.do?categoryAcc=GO:0006403)  [regulation of catalytic activity](http://www.pantherdb.org/panther/category.do?categoryAcc=GO:0050790) |
| [HUMAN\|Ensembl=ENSG00000248643\|UniProtKB=Q96PK6](http://www.pantherdb.org/genes/gene.do?acc=HUMAN%7CEnsembl=ENSG00000248643%7CUniProtKB=Q96PK6) | Q96PK6 | RNA-binding protein 14  [RBM14](http://www.pantherdb.org/genes/gene.do?acc=HUMAN%7CEnsembl=ENSG00000248643%7CUniProtKB=Q96PK6)  [ortholog](http://www.pantherdb.org/genes/gene.do?acc=HUMAN%7CEnsembl=ENSG00000248643%7CUniProtKB=Q96PK6#orthologs) | [DNA replication](http://www.pantherdb.org/panther/category.do?categoryAcc=GO:0006260)  [RNA splicing, via transesterification reactions](http://www.pantherdb.org/panther/category.do?categoryAcc=GO:0000375)  [transcription from RNA polymerase II promoter](http://www.pantherdb.org/panther/category.do?categoryAcc=GO:0006366)  [mRNA splicing, via spliceosome](http://www.pantherdb.org/panther/category.do?categoryAcc=GO:0000398)  [mRNA polyadenylation](http://www.pantherdb.org/panther/category.do?categoryAcc=GO:0006378)  [RNA splicing, via transesterification reactions](http://www.pantherdb.org/panther/category.do?categoryAcc=GO:0000375)  [protein metabolic process](http://www.pantherdb.org/panther/category.do?categoryAcc=GO:0019538)  [cell cycle](http://www.pantherdb.org/panther/category.do?categoryAcc=GO:0007049)  [ectoderm development](http://www.pantherdb.org/panther/category.do?categoryAcc=GO:0007398)  [nervous system development](http://www.pantherdb.org/panther/category.do?categoryAcc=GO:0007399) |
| [HUMAN\|Ensembl=ENSG00000076924\|UniProtKB=Q9HCS7](http://www.pantherdb.org/genes/gene.do?acc=HUMAN%7CEnsembl=ENSG00000076924%7CUniProtKB=Q9HCS7) | Q9HCS7 | Pre-mRNA-splicing factor SYF1  [XAB2](http://www.pantherdb.org/genes/gene.do?acc=HUMAN%7CEnsembl=ENSG00000076924%7CUniProtKB=Q9HCS7)  [ortholog](http://www.pantherdb.org/genes/gene.do?acc=HUMAN%7CEnsembl=ENSG00000076924%7CUniProtKB=Q9HCS7#orthologs) | [RNA splicing, via transesterification reactions](http://www.pantherdb.org/panther/category.do?categoryAcc=GO:0000375)  [mRNA splicing, via spliceosome](http://www.pantherdb.org/panther/category.do?categoryAcc=GO:0000398)  [RNA splicing, via transesterification reactions](http://www.pantherdb.org/panther/category.do?categoryAcc=GO:0000375)  [ectoderm development](http://www.pantherdb.org/panther/category.do?categoryAcc=GO:0007398)  [nervous system development](http://www.pantherdb.org/panther/category.do?categoryAcc=GO:0007399) |
| [HUMAN\|Ensembl=ENSG00000088205\|UniProtKB=Q9NVP1](http://www.pantherdb.org/genes/gene.do?acc=HUMAN%7CEnsembl=ENSG00000088205%7CUniProtKB=Q9NVP1) | Q9NVP1 | ATP-dependent RNA helicase DDX18  [DDX18](http://www.pantherdb.org/genes/gene.do?acc=HUMAN%7CEnsembl=ENSG00000088205%7CUniProtKB=Q9NVP1)  [ortholog](http://www.pantherdb.org/genes/gene.do?acc=HUMAN%7CEnsembl=ENSG00000088205%7CUniProtKB=Q9NVP1#orthologs) | [nucleobase-containing compound metabolic process](http://www.pantherdb.org/panther/category.do?categoryAcc=GO:0006139)  [translation](http://www.pantherdb.org/panther/category.do?categoryAcc=GO:0006412)  [regulation of translation](http://www.pantherdb.org/panther/category.do?categoryAcc=GO:0006417) |
| [HUMAN\|Ensembl=ENSG00000120158\|UniProtKB=Q9Y2P8](http://www.pantherdb.org/genes/gene.do?acc=HUMAN%7CEnsembl=ENSG00000120158%7CUniProtKB=Q9Y2P8) | Q9Y2P8 | RNA 3'-terminal phosphate cyclase-like protein  [RCL1](http://www.pantherdb.org/genes/gene.do?acc=HUMAN%7CEnsembl=ENSG00000120158%7CUniProtKB=Q9Y2P8)  [ortholog](http://www.pantherdb.org/genes/gene.do?acc=HUMAN%7CEnsembl=ENSG00000120158%7CUniProtKB=Q9Y2P8#orthologs) | [mRNA splicing, via spliceosome](http://www.pantherdb.org/panther/category.do?categoryAcc=GO:0000398) |

**Supplementary Table S4⏐List of proteins with decreased binding to β-catenin from IP-MS analysis.**

| Gene ID | Mapped ID | Gene Name/Symbol | GO Biological Process |
| --- | --- | --- | --- |
| [HUMAN\|Ensembl=ENSG00000010244\|UniProtKB=O43670](http://www.pantherdb.org/genes/gene.do?acc=HUMAN%7CEnsembl=ENSG00000010244%7CUniProtKB=O43670) | O43670 | Zinc finger protein 207  [ZNF207](http://www.pantherdb.org/genes/gene.do?acc=HUMAN%7CEnsembl=ENSG00000010244%7CUniProtKB=O43670)  [ortholog](http://www.pantherdb.org/genes/gene.do?acc=HUMAN%7CEnsembl=ENSG00000010244%7CUniProtKB=O43670#orthologs) | [nucleobase-containing compound metabolic process](http://www.pantherdb.org/panther/category.do?categoryAcc=GO:0006139) |
| [HUMAN\|Ensembl=ENSG00000125166\|UniProtKB=P00505](http://www.pantherdb.org/genes/gene.do?acc=HUMAN%7CEnsembl=ENSG00000125166%7CUniProtKB=P00505) | P00505 | Aspartate aminotransferase, mitochondrial  [GOT2](http://www.pantherdb.org/genes/gene.do?acc=HUMAN%7CEnsembl=ENSG00000125166%7CUniProtKB=P00505)  [ortholog](http://www.pantherdb.org/genes/gene.do?acc=HUMAN%7CEnsembl=ENSG00000125166%7CUniProtKB=P00505#orthologs) | [cellular amino acid metabolic process](http://www.pantherdb.org/panther/category.do?categoryAcc=GO:0006520) |
| [HUMAN\|Ensembl=ENSG00000111640\|UniProtKB=P04406](http://www.pantherdb.org/genes/gene.do?acc=HUMAN%7CEnsembl=ENSG00000111640%7CUniProtKB=P04406) | P04406 | Glyceraldehyde-3-phosphate dehydrogenase  [GAPDH](http://www.pantherdb.org/genes/gene.do?acc=HUMAN%7CEnsembl=ENSG00000111640%7CUniProtKB=P04406)  [ortholog](http://www.pantherdb.org/genes/gene.do?acc=HUMAN%7CEnsembl=ENSG00000111640%7CUniProtKB=P04406#orthologs) | [glycolysis](http://www.pantherdb.org/panther/category.do?categoryAcc=GO:0006096)  [glycolysis](http://www.pantherdb.org/panther/category.do?categoryAcc=GO:0006096) |
| [HUMAN\|Ensembl=ENSG00000163902\|UniProtKB=P04843](http://www.pantherdb.org/genes/gene.do?acc=HUMAN%7CEnsembl=ENSG00000163902%7CUniProtKB=P04843) | P04843 | Dolichyl-diphosphooligosaccharide--protein glycosyltransferase subunit 1  [RPN1](http://www.pantherdb.org/genes/gene.do?acc=HUMAN%7CEnsembl=ENSG00000163902%7CUniProtKB=P04843)  [ortholog](http://www.pantherdb.org/genes/gene.do?acc=HUMAN%7CEnsembl=ENSG00000163902%7CUniProtKB=P04843#orthologs) | [translation](http://www.pantherdb.org/panther/category.do?categoryAcc=GO:0006412)  [protein glycosylation](http://www.pantherdb.org/panther/category.do?categoryAcc=GO:0006486) |
| [HUMAN\|Ensembl=ENSG00000177600\|UniProtKB=P05387](http://www.pantherdb.org/genes/gene.do?acc=HUMAN%7CEnsembl=ENSG00000177600%7CUniProtKB=P05387) | P05387 | 60S acidic ribosomal protein P2  [RPLP2](http://www.pantherdb.org/genes/gene.do?acc=HUMAN%7CEnsembl=ENSG00000177600%7CUniProtKB=P05387)  [ortholog](http://www.pantherdb.org/genes/gene.do?acc=HUMAN%7CEnsembl=ENSG00000177600%7CUniProtKB=P05387#orthologs) | [translation](http://www.pantherdb.org/panther/category.do?categoryAcc=GO:0006412) |
| [HUMAN\|Ensembl=ENSG00000008018\|UniProtKB=P20618](http://www.pantherdb.org/genes/gene.do?acc=HUMAN%7CEnsembl=ENSG00000008018%7CUniProtKB=P20618) | P20618 | Proteasome subunit beta type-1  [PSMB1](http://www.pantherdb.org/genes/gene.do?acc=HUMAN%7CEnsembl=ENSG00000008018%7CUniProtKB=P20618)  [ortholog](http://www.pantherdb.org/genes/gene.do?acc=HUMAN%7CEnsembl=ENSG00000008018%7CUniProtKB=P20618#orthologs) | [proteolysis](http://www.pantherdb.org/panther/category.do?categoryAcc=GO:0006508) |
| [HUMAN\|Ensembl=ENSG00000041357\|UniProtKB=P25789](http://www.pantherdb.org/genes/gene.do?acc=HUMAN%7CEnsembl=ENSG00000041357%7CUniProtKB=P25789) | P25789 | Proteasome subunit alpha type-4  [PSMA4](http://www.pantherdb.org/genes/gene.do?acc=HUMAN%7CEnsembl=ENSG00000041357%7CUniProtKB=P25789)  [ortholog](http://www.pantherdb.org/genes/gene.do?acc=HUMAN%7CEnsembl=ENSG00000041357%7CUniProtKB=P25789#orthologs) | [proteolysis](http://www.pantherdb.org/panther/category.do?categoryAcc=GO:0006508) |
| [HUMAN\|Ensembl=ENSG00000167004\|UniProtKB=P30101](http://www.pantherdb.org/genes/gene.do?acc=HUMAN%7CEnsembl=ENSG00000167004%7CUniProtKB=P30101) | P30101 | Protein disulfide-isomerase A3  [PDIA3](http://www.pantherdb.org/genes/gene.do?acc=HUMAN%7CEnsembl=ENSG00000167004%7CUniProtKB=P30101)  [ortholog](http://www.pantherdb.org/genes/gene.do?acc=HUMAN%7CEnsembl=ENSG00000167004%7CUniProtKB=P30101#orthologs) | [protein folding](http://www.pantherdb.org/panther/category.do?categoryAcc=GO:0006457)  [cellular protein modification process](http://www.pantherdb.org/panther/category.do?categoryAcc=GO:0006464) |
| [HUMAN\|Ensembl=ENSG00000168906\|UniProtKB=P31153](http://www.pantherdb.org/genes/gene.do?acc=HUMAN%7CEnsembl=ENSG00000168906%7CUniProtKB=P31153) | P31153 | S-adenosylmethionine synthase isoform type-2  [MAT2A](http://www.pantherdb.org/genes/gene.do?acc=HUMAN%7CEnsembl=ENSG00000168906%7CUniProtKB=P31153)  [ortholog](http://www.pantherdb.org/genes/gene.do?acc=HUMAN%7CEnsembl=ENSG00000168906%7CUniProtKB=P31153#orthologs) | [cellular amino acid metabolic process](http://www.pantherdb.org/panther/category.do?categoryAcc=GO:0006520) |
| [HUMAN\|Ensembl=ENSG00000170606\|UniProtKB=P34932](http://www.pantherdb.org/genes/gene.do?acc=HUMAN%7CEnsembl=ENSG00000170606%7CUniProtKB=P34932) | P34932 | Heat shock 70 kDa protein 4  [HSPA4](http://www.pantherdb.org/genes/gene.do?acc=HUMAN%7CEnsembl=ENSG00000170606%7CUniProtKB=P34932)  [ortholog](http://www.pantherdb.org/genes/gene.do?acc=HUMAN%7CEnsembl=ENSG00000170606%7CUniProtKB=P34932#orthologs) | [immune system process](http://www.pantherdb.org/panther/category.do?categoryAcc=GO:0002376)  [protein folding](http://www.pantherdb.org/panther/category.do?categoryAcc=GO:0006457)  [protein complex assembly](http://www.pantherdb.org/panther/category.do?categoryAcc=GO:0006461)  [response to stress](http://www.pantherdb.org/panther/category.do?categoryAcc=GO:0006950)  [protein complex biogenesis](http://www.pantherdb.org/panther/category.do?categoryAcc=GO:0070271) |
| [HUMAN\|Ensembl=ENSG00000150753\|UniProtKB=P48643](http://www.pantherdb.org/genes/gene.do?acc=HUMAN%7CEnsembl=ENSG00000150753%7CUniProtKB=P48643) | P48643 | T-complex protein 1 subunit epsilon  [CCT5](http://www.pantherdb.org/genes/gene.do?acc=HUMAN%7CEnsembl=ENSG00000150753%7CUniProtKB=P48643)  [ortholog](http://www.pantherdb.org/genes/gene.do?acc=HUMAN%7CEnsembl=ENSG00000150753%7CUniProtKB=P48643#orthologs) | [protein folding](http://www.pantherdb.org/panther/category.do?categoryAcc=GO:0006457) |
| [HUMAN\|Ensembl=ENSG00000100380\|UniProtKB=P50502](http://www.pantherdb.org/genes/gene.do?acc=HUMAN%7CEnsembl=ENSG00000100380%7CUniProtKB=P50502) | P50502 | Hsc70-interacting protein  [ST13](http://www.pantherdb.org/genes/gene.do?acc=HUMAN%7CEnsembl=ENSG00000100380%7CUniProtKB=P50502)  [ortholog](http://www.pantherdb.org/genes/gene.do?acc=HUMAN%7CEnsembl=ENSG00000100380%7CUniProtKB=P50502#orthologs) | [protein folding](http://www.pantherdb.org/panther/category.do?categoryAcc=GO:0006457)  [response to stress](http://www.pantherdb.org/panther/category.do?categoryAcc=GO:0006950) |
| [HUMAN\|Ensembl=ENSG00000065427\|UniProtKB=Q15046](http://www.pantherdb.org/genes/gene.do?acc=HUMAN%7CEnsembl=ENSG00000065427%7CUniProtKB=Q15046) | Q15046 | Lysine--tRNA ligase  [KARS](http://www.pantherdb.org/genes/gene.do?acc=HUMAN%7CEnsembl=ENSG00000065427%7CUniProtKB=Q15046)  [ortholog](http://www.pantherdb.org/genes/gene.do?acc=HUMAN%7CEnsembl=ENSG00000065427%7CUniProtKB=Q15046#orthologs) | [translation](http://www.pantherdb.org/panther/category.do?categoryAcc=GO:0006412) |
| [HUMAN\|Ensembl=ENSG00000111196\|UniProtKB=Q96A72](http://www.pantherdb.org/genes/gene.do?acc=HUMAN%7CEnsembl=ENSG00000111196%7CUniProtKB=Q96A72) | Q96A72 | Protein mago nashi homolog 2  [MAGOHB](http://www.pantherdb.org/genes/gene.do?acc=HUMAN%7CEnsembl=ENSG00000111196%7CUniProtKB=Q96A72)  [ortholog](http://www.pantherdb.org/genes/gene.do?acc=HUMAN%7CEnsembl=ENSG00000111196%7CUniProtKB=Q96A72#orthologs) | [female gamete generation](http://www.pantherdb.org/panther/category.do?categoryAcc=GO:0007292)  [nucleobase-containing compound metabolic process](http://www.pantherdb.org/panther/category.do?categoryAcc=GO:0006139)  [sex determination](http://www.pantherdb.org/panther/category.do?categoryAcc=GO:0007530)  [RNA localization](http://www.pantherdb.org/panther/category.do?categoryAcc=GO:0006403) |
| [HUMAN\|Ensembl=ENSG00000135018\|UniProtKB=Q9UMX0](http://www.pantherdb.org/genes/gene.do?acc=HUMAN%7CEnsembl=ENSG00000135018%7CUniProtKB=Q9UMX0) | Q9UMX0 | Ubiquilin-1  [UBQLN1](http://www.pantherdb.org/genes/gene.do?acc=HUMAN%7CEnsembl=ENSG00000135018%7CUniProtKB=Q9UMX0)  [ortholog](http://www.pantherdb.org/genes/gene.do?acc=HUMAN%7CEnsembl=ENSG00000135018%7CUniProtKB=Q9UMX0#orthologs) | [proteolysis](http://www.pantherdb.org/panther/category.do?categoryAcc=GO:0006508) |
| [HUMAN\|Ensembl=ENSG00000134333\|UniProtKB=P00338](http://www.pantherdb.org/genes/gene.do?acc=HUMAN%7CEnsembl=ENSG00000134333%7CUniProtKB=P00338) | P00338 | L-lactate dehydrogenase A chain  [LDHA](http://www.pantherdb.org/genes/gene.do?acc=HUMAN%7CEnsembl=ENSG00000134333%7CUniProtKB=P00338)  [ortholog](http://www.pantherdb.org/genes/gene.do?acc=HUMAN%7CEnsembl=ENSG00000134333%7CUniProtKB=P00338#orthologs) | [glycolysis](http://www.pantherdb.org/panther/category.do?categoryAcc=GO:0006096)  [glycolysis](http://www.pantherdb.org/panther/category.do?categoryAcc=GO:0006096)  [tricarboxylic acid cycle](http://www.pantherdb.org/panther/category.do?categoryAcc=GO:0006099) |
| [HUMAN\|Ensembl=ENSG00000155660\|UniProtKB=P13667](http://www.pantherdb.org/genes/gene.do?acc=HUMAN%7CEnsembl=ENSG00000155660%7CUniProtKB=P13667) | P13667 | Protein disulfide-isomerase A4  [PDIA4](http://www.pantherdb.org/genes/gene.do?acc=HUMAN%7CEnsembl=ENSG00000155660%7CUniProtKB=P13667)  [ortholog](http://www.pantherdb.org/genes/gene.do?acc=HUMAN%7CEnsembl=ENSG00000155660%7CUniProtKB=P13667#orthologs) | [protein folding](http://www.pantherdb.org/panther/category.do?categoryAcc=GO:0006457)  [cellular protein modification process](http://www.pantherdb.org/panther/category.do?categoryAcc=GO:0006464) |
| [HUMAN\|Ensembl=ENSG00000130985\|UniProtKB=P22314](http://www.pantherdb.org/genes/gene.do?acc=HUMAN%7CEnsembl=ENSG00000130985%7CUniProtKB=P22314) | P22314 | Ubiquitin-like modifier-activating enzyme 1  [UBA1](http://www.pantherdb.org/genes/gene.do?acc=HUMAN%7CEnsembl=ENSG00000130985%7CUniProtKB=P22314)  [ortholog](http://www.pantherdb.org/genes/gene.do?acc=HUMAN%7CEnsembl=ENSG00000130985%7CUniProtKB=P22314#orthologs) | [coenzyme metabolic process](http://www.pantherdb.org/panther/category.do?categoryAcc=GO:0006732)  [cellular protein modification process](http://www.pantherdb.org/panther/category.do?categoryAcc=GO:0006464)  [proteolysis](http://www.pantherdb.org/panther/category.do?categoryAcc=GO:0006508)  [cell communication](http://www.pantherdb.org/panther/category.do?categoryAcc=GO:0007154)  [intracellular protein transport](http://www.pantherdb.org/panther/category.do?categoryAcc=GO:0006886)  [nuclear transport](http://www.pantherdb.org/panther/category.do?categoryAcc=GO:0051169) |
|  |  |  |  |
| [HUMAN\|Ensembl=ENSG00000130741\|UniProtKB=P41091](http://www.pantherdb.org/genes/gene.do?acc=HUMAN%7CEnsembl=ENSG00000130741%7CUniProtKB=P41091) | P41091 | Eukaryotic translation initiation factor 2 subunit 3  [EIF2S3](http://www.pantherdb.org/genes/gene.do?acc=HUMAN%7CEnsembl=ENSG00000130741%7CUniProtKB=P41091)  [ortholog](http://www.pantherdb.org/genes/gene.do?acc=HUMAN%7CEnsembl=ENSG00000130741%7CUniProtKB=P41091#orthologs) | [translation](http://www.pantherdb.org/panther/category.do?categoryAcc=GO:0006412)  [regulation of translation](http://www.pantherdb.org/panther/category.do?categoryAcc=GO:0006417) |
| [HUMAN\|Ensembl=ENSG00000241837\|UniProtKB=P48047](http://www.pantherdb.org/genes/gene.do?acc=HUMAN%7CEnsembl=ENSG00000241837%7CUniProtKB=P48047) | P48047 | ATP synthase subunit O, mitochondrial  [ATP5O](http://www.pantherdb.org/genes/gene.do?acc=HUMAN%7CEnsembl=ENSG00000241837%7CUniProtKB=P48047)  [ortholog](http://www.pantherdb.org/genes/gene.do?acc=HUMAN%7CEnsembl=ENSG00000241837%7CUniProtKB=P48047#orthologs) | [coenzyme metabolic process](http://www.pantherdb.org/panther/category.do?categoryAcc=GO:0006732)  [nucleobase-containing compound metabolic process](http://www.pantherdb.org/panther/category.do?categoryAcc=GO:0006139)  [cation transport](http://www.pantherdb.org/panther/category.do?categoryAcc=GO:0006812) |
| [HUMAN\|Ensembl=ENSG00000134684\|UniProtKB=P54577](http://www.pantherdb.org/genes/gene.do?acc=HUMAN%7CEnsembl=ENSG00000134684%7CUniProtKB=P54577) | P54577 | Tyrosine--tRNA ligase, cytoplasmic  [YARS](http://www.pantherdb.org/genes/gene.do?acc=HUMAN%7CEnsembl=ENSG00000134684%7CUniProtKB=P54577)  [ortholog](http://www.pantherdb.org/genes/gene.do?acc=HUMAN%7CEnsembl=ENSG00000134684%7CUniProtKB=P54577#orthologs) | [translation](http://www.pantherdb.org/panther/category.do?categoryAcc=GO:0006412) |
| [HUMAN\|Ensembl=ENSG00000131467\|UniProtKB=P61289](http://www.pantherdb.org/genes/gene.do?acc=HUMAN%7CEnsembl=ENSG00000131467%7CUniProtKB=P61289) | P61289 | Proteasome activator complex subunit 3  [PSME3](http://www.pantherdb.org/genes/gene.do?acc=HUMAN%7CEnsembl=ENSG00000131467%7CUniProtKB=P61289)  [ortholog](http://www.pantherdb.org/genes/gene.do?acc=HUMAN%7CEnsembl=ENSG00000131467%7CUniProtKB=P61289#orthologs) | [proteolysis](http://www.pantherdb.org/panther/category.do?categoryAcc=GO:0006508) |
| [HUMAN\|Ensembl=ENSG00000067057\|UniProtKB=Q01813](http://www.pantherdb.org/genes/gene.do?acc=HUMAN%7CEnsembl=ENSG00000067057%7CUniProtKB=Q01813) | Q01813 | 6-phosphofructokinase type C  [PFKP](http://www.pantherdb.org/genes/gene.do?acc=HUMAN%7CEnsembl=ENSG00000067057%7CUniProtKB=Q01813)  [ortholog](http://www.pantherdb.org/genes/gene.do?acc=HUMAN%7CEnsembl=ENSG00000067057%7CUniProtKB=Q01813#orthologs) | [glycolysis](http://www.pantherdb.org/panther/category.do?categoryAcc=GO:0006096)  [glycolysis](http://www.pantherdb.org/panther/category.do?categoryAcc=GO:0006096) |
| [HUMAN\|Ensembl=ENSG00000104823\|UniProtKB=Q13011](http://www.pantherdb.org/genes/gene.do?acc=HUMAN%7CEnsembl=ENSG00000104823%7CUniProtKB=Q13011) | Q13011 | Delta(3,5)-Delta(2,4)-dienoyl-CoA isomerase, mitochondrial  [ECH1](http://www.pantherdb.org/genes/gene.do?acc=HUMAN%7CEnsembl=ENSG00000104823%7CUniProtKB=Q13011)  [ortholog](http://www.pantherdb.org/genes/gene.do?acc=HUMAN%7CEnsembl=ENSG00000104823%7CUniProtKB=Q13011#orthologs) | [coenzyme metabolic process](http://www.pantherdb.org/panther/category.do?categoryAcc=GO:0006732)  [vitamin biosynthetic process](http://www.pantherdb.org/panther/category.do?categoryAcc=GO:0009110)  [carbohydrate metabolic process](http://www.pantherdb.org/panther/category.do?categoryAcc=GO:0005975)  [fatty acid beta-oxidation](http://www.pantherdb.org/panther/category.do?categoryAcc=GO:0006635) |
| [HUMAN\|Ensembl=ENSG00000110958\|UniProtKB=Q15185](http://www.pantherdb.org/genes/gene.do?acc=HUMAN%7CEnsembl=ENSG00000110958%7CUniProtKB=Q15185) | Q15185 | Prostaglandin E synthase 3  [PTGES3](http://www.pantherdb.org/genes/gene.do?acc=HUMAN%7CEnsembl=ENSG00000110958%7CUniProtKB=Q15185)  [ortholog](http://www.pantherdb.org/genes/gene.do?acc=HUMAN%7CEnsembl=ENSG00000110958%7CUniProtKB=Q15185#orthologs) | [protein folding](http://www.pantherdb.org/panther/category.do?categoryAcc=GO:0006457)  [protein complex assembly](http://www.pantherdb.org/panther/category.do?categoryAcc=GO:0006461)  [protein complex biogenesis](http://www.pantherdb.org/panther/category.do?categoryAcc=GO:0070271) |
| [HUMAN\|Ensembl=ENSG00000100836\|UniProtKB=Q86U42](http://www.pantherdb.org/genes/gene.do?acc=HUMAN%7CEnsembl=ENSG00000100836%7CUniProtKB=Q86U42) | Q86U42 | Polyadenylate-binding protein 2  [PABPN1](http://www.pantherdb.org/genes/gene.do?acc=HUMAN%7CEnsembl=ENSG00000100836%7CUniProtKB=Q86U42)  [ortholog](http://www.pantherdb.org/genes/gene.do?acc=HUMAN%7CEnsembl=ENSG00000100836%7CUniProtKB=Q86U42#orthologs) | [mRNA processing](http://www.pantherdb.org/panther/category.do?categoryAcc=GO:0006397) |
| [HUMAN\|Ensembl=ENSG00000107164\|UniProtKB=Q96I24](http://www.pantherdb.org/genes/gene.do?acc=HUMAN%7CEnsembl=ENSG00000107164%7CUniProtKB=Q96I24) | Q96I24 | Far upstream element-binding protein 3  [FUBP3](http://www.pantherdb.org/genes/gene.do?acc=HUMAN%7CEnsembl=ENSG00000107164%7CUniProtKB=Q96I24)  [ortholog](http://www.pantherdb.org/genes/gene.do?acc=HUMAN%7CEnsembl=ENSG00000107164%7CUniProtKB=Q96I24#orthologs) | [induction of apoptosis](http://www.pantherdb.org/panther/category.do?categoryAcc=GO:0006917)  [RNA splicing, via transesterification reactions](http://www.pantherdb.org/panther/category.do?categoryAcc=GO:0000375)  [transcription from RNA polymerase II promoter](http://www.pantherdb.org/panther/category.do?categoryAcc=GO:0006366)  [mRNA splicing, via spliceosome](http://www.pantherdb.org/panther/category.do?categoryAcc=GO:0000398)  [RNA splicing, via transesterification reactions](http://www.pantherdb.org/panther/category.do?categoryAcc=GO:0000375)  [protein metabolic process](http://www.pantherdb.org/panther/category.do?categoryAcc=GO:0019538)  [cell communication](http://www.pantherdb.org/panther/category.do?categoryAcc=GO:0007154)  [neurological system process](http://www.pantherdb.org/panther/category.do?categoryAcc=GO:0050877)  [induction of apoptosis](http://www.pantherdb.org/panther/category.do?categoryAcc=GO:0006917)  [intracellular protein transport](http://www.pantherdb.org/panther/category.do?categoryAcc=GO:0006886)  [nuclear transport](http://www.pantherdb.org/panther/category.do?categoryAcc=GO:0051169) |
|  |  |  |  |
|  |  |  |  |
| [HUMAN\|Ensembl=ENSG00000143401\|UniProtKB=Q9BTT0](http://www.pantherdb.org/genes/gene.do?acc=HUMAN%7CEnsembl=ENSG00000143401%7CUniProtKB=Q9BTT0) | Q9BTT0 | Acidic leucine-rich nuclear phosphoprotein 32 family member E  [ANP32E](http://www.pantherdb.org/genes/gene.do?acc=HUMAN%7CEnsembl=ENSG00000143401%7CUniProtKB=Q9BTT0)  [ortholog](http://www.pantherdb.org/genes/gene.do?acc=HUMAN%7CEnsembl=ENSG00000143401%7CUniProtKB=Q9BTT0#orthologs) | [metabolic process](http://www.pantherdb.org/panther/category.do?categoryAcc=GO:0008152)  [regulation of catalytic activity](http://www.pantherdb.org/panther/category.do?categoryAcc=GO:0050790) |
| [HUMAN\|Ensembl=ENSG00000108010\|UniProtKB=O76003](http://www.pantherdb.org/genes/gene.do?acc=HUMAN%7CEnsembl=ENSG00000108010%7CUniProtKB=O76003) | O76003 | Glutaredoxin-3  [GLRX3](http://www.pantherdb.org/genes/gene.do?acc=HUMAN%7CEnsembl=ENSG00000108010%7CUniProtKB=O76003)  [ortholog](http://www.pantherdb.org/genes/gene.do?acc=HUMAN%7CEnsembl=ENSG00000108010%7CUniProtKB=O76003#orthologs) | [respiratory electron transport chain](http://www.pantherdb.org/panther/category.do?categoryAcc=GO:0022904)  [sulfur compound metabolic process](http://www.pantherdb.org/panther/category.do?categoryAcc=GO:0006790)  [response to stress](http://www.pantherdb.org/panther/category.do?categoryAcc=GO:0006950) |
| [HUMAN\|Ensembl=ENSG00000162813\|UniProtKB=O95861](http://www.pantherdb.org/genes/gene.do?acc=HUMAN%7CEnsembl=ENSG00000162813%7CUniProtKB=O95861) | O95861 | 3'(2'),5'-bisphosphate nucleotidase 1  [BPNT1](http://www.pantherdb.org/genes/gene.do?acc=HUMAN%7CEnsembl=ENSG00000162813%7CUniProtKB=O95861)  [ortholog](http://www.pantherdb.org/genes/gene.do?acc=HUMAN%7CEnsembl=ENSG00000162813%7CUniProtKB=O95861#orthologs) | [sulfur compound metabolic process](http://www.pantherdb.org/panther/category.do?categoryAcc=GO:0006790)  [phospholipid metabolic process](http://www.pantherdb.org/panther/category.do?categoryAcc=GO:0006644)  [nucleobase-containing compound metabolic process](http://www.pantherdb.org/panther/category.do?categoryAcc=GO:0006139)  [phospholipid metabolic process](http://www.pantherdb.org/panther/category.do?categoryAcc=GO:0006644)  [cellular process](http://www.pantherdb.org/panther/category.do?categoryAcc=GO:0009987) |
| [HUMAN\|Ensembl=ENSG00000121552\|UniProtKB=P01040](http://www.pantherdb.org/genes/gene.do?acc=HUMAN%7CEnsembl=ENSG00000121552%7CUniProtKB=P01040) | P01040 | Cystatin-A  [CSTA](http://www.pantherdb.org/genes/gene.do?acc=HUMAN%7CEnsembl=ENSG00000121552%7CUniProtKB=P01040)  [ortholog](http://www.pantherdb.org/genes/gene.do?acc=HUMAN%7CEnsembl=ENSG00000121552%7CUniProtKB=P01040#orthologs) | [proteolysis](http://www.pantherdb.org/panther/category.do?categoryAcc=GO:0006508)  [regulation of catalytic activity](http://www.pantherdb.org/panther/category.do?categoryAcc=GO:0050790) |
| [HUMAN\|Ensembl=ENSG00000160213\|UniProtKB=P04080](http://www.pantherdb.org/genes/gene.do?acc=HUMAN%7CEnsembl=ENSG00000160213%7CUniProtKB=P04080) | P04080 | Cystatin-B  [CSTB](http://www.pantherdb.org/genes/gene.do?acc=HUMAN%7CEnsembl=ENSG00000160213%7CUniProtKB=P04080)  [ortholog](http://www.pantherdb.org/genes/gene.do?acc=HUMAN%7CEnsembl=ENSG00000160213%7CUniProtKB=P04080#orthologs) | [proteolysis](http://www.pantherdb.org/panther/category.do?categoryAcc=GO:0006508)  [regulation of catalytic activity](http://www.pantherdb.org/panther/category.do?categoryAcc=GO:0050790) |
| [HUMAN\|Ensembl=ENSG00000170312\|UniProtKB=P06493](http://www.pantherdb.org/genes/gene.do?acc=HUMAN%7CEnsembl=ENSG00000170312%7CUniProtKB=P06493) | P06493 | Cyclin-dependent kinase 1  [CDK1](http://www.pantherdb.org/genes/gene.do?acc=HUMAN%7CEnsembl=ENSG00000170312%7CUniProtKB=P06493)  [ortholog](http://www.pantherdb.org/genes/gene.do?acc=HUMAN%7CEnsembl=ENSG00000170312%7CUniProtKB=P06493#orthologs) | [glycogen metabolic process](http://www.pantherdb.org/panther/category.do?categoryAcc=GO:0005977)  [protein phosphorylation](http://www.pantherdb.org/panther/category.do?categoryAcc=GO:0006468)  [mitosis](http://www.pantherdb.org/panther/category.do?categoryAcc=GO:0007067)  [cell communication](http://www.pantherdb.org/panther/category.do?categoryAcc=GO:0007154) |
| [HUMAN\|Ensembl=ENSG00000182199\|UniProtKB=P34897](http://www.pantherdb.org/genes/gene.do?acc=HUMAN%7CEnsembl=ENSG00000182199%7CUniProtKB=P34897) | P34897 | Serine hydroxymethyltransferase, mitochondrial  [SHMT2](http://www.pantherdb.org/genes/gene.do?acc=HUMAN%7CEnsembl=ENSG00000182199%7CUniProtKB=P34897)  [ortholog](http://www.pantherdb.org/genes/gene.do?acc=HUMAN%7CEnsembl=ENSG00000182199%7CUniProtKB=P34897#orthologs) | [nucleobase-containing compound metabolic process](http://www.pantherdb.org/panther/category.do?categoryAcc=GO:0006139)  [cellular amino acid metabolic process](http://www.pantherdb.org/panther/category.do?categoryAcc=GO:0006520) |
| [HUMAN\|Ensembl=ENSG00000072778\|UniProtKB=P49748](http://www.pantherdb.org/genes/gene.do?acc=HUMAN%7CEnsembl=ENSG00000072778%7CUniProtKB=P49748) | P49748 | Very long-chain specific acyl-CoA dehydrogenase, mitochondrial  [ACADVL](http://www.pantherdb.org/genes/gene.do?acc=HUMAN%7CEnsembl=ENSG00000072778%7CUniProtKB=P49748)  [ortholog](http://www.pantherdb.org/genes/gene.do?acc=HUMAN%7CEnsembl=ENSG00000072778%7CUniProtKB=P49748#orthologs) | [respiratory electron transport chain](http://www.pantherdb.org/panther/category.do?categoryAcc=GO:0022904)  [acyl-CoA metabolic process](http://www.pantherdb.org/panther/category.do?categoryAcc=GO:0006637)  [nitrogen compound metabolic process](http://www.pantherdb.org/panther/category.do?categoryAcc=GO:0006807)  [fatty acid beta-oxidation](http://www.pantherdb.org/panther/category.do?categoryAcc=GO:0006635)  [acyl-CoA metabolic process](http://www.pantherdb.org/panther/category.do?categoryAcc=GO:0006637) |
| [HUMAN\|Ensembl=ENSG00000127314\|UniProtKB=P61224](http://www.pantherdb.org/genes/gene.do?acc=HUMAN%7CEnsembl=ENSG00000127314%7CUniProtKB=P61224) | P61224 | Ras-related protein Rap-1b  [RAP1B](http://www.pantherdb.org/genes/gene.do?acc=HUMAN%7CEnsembl=ENSG00000127314%7CUniProtKB=P61224)  [ortholog](http://www.pantherdb.org/genes/gene.do?acc=HUMAN%7CEnsembl=ENSG00000127314%7CUniProtKB=P61224#orthologs) | [metabolic process](http://www.pantherdb.org/panther/category.do?categoryAcc=GO:0008152)  [synaptic transmission](http://www.pantherdb.org/panther/category.do?categoryAcc=GO:0007268)  [cell adhesion](http://www.pantherdb.org/panther/category.do?categoryAcc=GO:0007155)  [neurological system process](http://www.pantherdb.org/panther/category.do?categoryAcc=GO:0050877)  [intracellular protein transport](http://www.pantherdb.org/panther/category.do?categoryAcc=GO:0006886)  [receptor-mediated endocytosis](http://www.pantherdb.org/panther/category.do?categoryAcc=GO:0006898) |
| [HUMAN\|Ensembl=ENSG00000103257\|UniProtKB=Q01650](http://www.pantherdb.org/genes/gene.do?acc=HUMAN%7CEnsembl=ENSG00000103257%7CUniProtKB=Q01650) | Q01650 | Large neutral amino acids transporter small subunit 1  [SLC7A5](http://www.pantherdb.org/genes/gene.do?acc=HUMAN%7CEnsembl=ENSG00000103257%7CUniProtKB=Q01650)  [ortholog](http://www.pantherdb.org/genes/gene.do?acc=HUMAN%7CEnsembl=ENSG00000103257%7CUniProtKB=Q01650#orthologs) | [cellular amino acid metabolic process](http://www.pantherdb.org/panther/category.do?categoryAcc=GO:0006520)  [amino acid transport](http://www.pantherdb.org/panther/category.do?categoryAcc=GO:0006865) |
| [HUMAN\|Ensembl=ENSG00000197157\|UniProtKB=Q7KZF4](http://www.pantherdb.org/genes/gene.do?acc=HUMAN%7CEnsembl=ENSG00000197157%7CUniProtKB=Q7KZF4) | Q7KZF4 | Staphylococcal nuclease domain-containing protein 1  [SND1](http://www.pantherdb.org/genes/gene.do?acc=HUMAN%7CEnsembl=ENSG00000197157%7CUniProtKB=Q7KZF4)  [ortholog](http://www.pantherdb.org/genes/gene.do?acc=HUMAN%7CEnsembl=ENSG00000197157%7CUniProtKB=Q7KZF4#orthologs) | [transcription from RNA polymerase II promoter](http://www.pantherdb.org/panther/category.do?categoryAcc=GO:0006366) |
| [HUMAN\|Ensembl=ENSG00000168872\|UniProtKB=Q9NUU7](http://www.pantherdb.org/genes/gene.do?acc=HUMAN%7CEnsembl=ENSG00000168872%7CUniProtKB=Q9NUU7) | Q9NUU7 | ATP-dependent RNA helicase DDX19A  [DDX19A](http://www.pantherdb.org/genes/gene.do?acc=HUMAN%7CEnsembl=ENSG00000168872%7CUniProtKB=Q9NUU7)  [ortholog](http://www.pantherdb.org/genes/gene.do?acc=HUMAN%7CEnsembl=ENSG00000168872%7CUniProtKB=Q9NUU7#orthologs) | [nucleobase-containing compound metabolic process](http://www.pantherdb.org/panther/category.do?categoryAcc=GO:0006139)  [translation](http://www.pantherdb.org/panther/category.do?categoryAcc=GO:0006412)  [regulation of translation](http://www.pantherdb.org/panther/category.do?categoryAcc=GO:0006417) |
| [HUMAN\|Ensembl=ENSG00000134440\|UniProtKB=O43776](http://www.pantherdb.org/genes/gene.do?acc=HUMAN%7CEnsembl=ENSG00000134440%7CUniProtKB=O43776) | O43776 | Asparagine--tRNA ligase, cytoplasmic  [NARS](http://www.pantherdb.org/genes/gene.do?acc=HUMAN%7CEnsembl=ENSG00000134440%7CUniProtKB=O43776)  [ortholog](http://www.pantherdb.org/genes/gene.do?acc=HUMAN%7CEnsembl=ENSG00000134440%7CUniProtKB=O43776#orthologs) | [translation](http://www.pantherdb.org/panther/category.do?categoryAcc=GO:0006412) |
| [HUMAN\|Ensembl=ENSG00000111716\|UniProtKB=P07195](http://www.pantherdb.org/genes/gene.do?acc=HUMAN%7CEnsembl=ENSG00000111716%7CUniProtKB=P07195) | P07195 | L-lactate dehydrogenase B chain  [LDHB](http://www.pantherdb.org/genes/gene.do?acc=HUMAN%7CEnsembl=ENSG00000111716%7CUniProtKB=P07195)  [ortholog](http://www.pantherdb.org/genes/gene.do?acc=HUMAN%7CEnsembl=ENSG00000111716%7CUniProtKB=P07195#orthologs) | [glycolysis](http://www.pantherdb.org/panther/category.do?categoryAcc=GO:0006096)  [glycolysis](http://www.pantherdb.org/panther/category.do?categoryAcc=GO:0006096)  [tricarboxylic acid cycle](http://www.pantherdb.org/panther/category.do?categoryAcc=GO:0006099) |
| [HUMAN\|Ensembl=ENSG00000175592\|UniProtKB=P15407](http://www.pantherdb.org/genes/gene.do?acc=HUMAN%7CEnsembl=ENSG00000175592%7CUniProtKB=P15407) | P15407 | Fos-related antigen 1  [FOSL1](http://www.pantherdb.org/genes/gene.do?acc=HUMAN%7CEnsembl=ENSG00000175592%7CUniProtKB=P15407)  [ortholog](http://www.pantherdb.org/genes/gene.do?acc=HUMAN%7CEnsembl=ENSG00000175592%7CUniProtKB=P15407#orthologs) | [immune system process](http://www.pantherdb.org/panther/category.do?categoryAcc=GO:0002376)  [induction of apoptosis](http://www.pantherdb.org/panther/category.do?categoryAcc=GO:0006917)  [transcription from RNA polymerase II promoter](http://www.pantherdb.org/panther/category.do?categoryAcc=GO:0006366)  [cell cycle](http://www.pantherdb.org/panther/category.do?categoryAcc=GO:0007049)  [neurological system process](http://www.pantherdb.org/panther/category.do?categoryAcc=GO:0050877)  [induction of apoptosis](http://www.pantherdb.org/panther/category.do?categoryAcc=GO:0006917)  [regulation of transcription from RNA polymerase II promoter](http://www.pantherdb.org/panther/category.do?categoryAcc=GO:0006357) |
| [HUMAN\|Ensembl=ENSG00000120053\|UniProtKB=P17174](http://www.pantherdb.org/genes/gene.do?acc=HUMAN%7CEnsembl=ENSG00000120053%7CUniProtKB=P17174) | P17174 | Aspartate aminotransferase, cytoplasmic  [GOT1](http://www.pantherdb.org/genes/gene.do?acc=HUMAN%7CEnsembl=ENSG00000120053%7CUniProtKB=P17174)  [ortholog](http://www.pantherdb.org/genes/gene.do?acc=HUMAN%7CEnsembl=ENSG00000120053%7CUniProtKB=P17174#orthologs) | [cellular amino acid metabolic process](http://www.pantherdb.org/panther/category.do?categoryAcc=GO:0006520) |
| [HUMAN\|Ensembl=ENSG00000125977\|UniProtKB=P20042](http://www.pantherdb.org/genes/gene.do?acc=HUMAN%7CEnsembl=ENSG00000125977%7CUniProtKB=P20042) | P20042 | Eukaryotic translation initiation factor 2 subunit 2  [EIF2S2](http://www.pantherdb.org/genes/gene.do?acc=HUMAN%7CEnsembl=ENSG00000125977%7CUniProtKB=P20042)  [ortholog](http://www.pantherdb.org/genes/gene.do?acc=HUMAN%7CEnsembl=ENSG00000125977%7CUniProtKB=P20042#orthologs) | [translation](http://www.pantherdb.org/panther/category.do?categoryAcc=GO:0006412)  [regulation of translation](http://www.pantherdb.org/panther/category.do?categoryAcc=GO:0006417) |
| [HUMAN\|Ensembl=ENSG00000152234\|UniProtKB=P25705](http://www.pantherdb.org/genes/gene.do?acc=HUMAN%7CEnsembl=ENSG00000152234%7CUniProtKB=P25705) | P25705 | ATP synthase subunit alpha, mitochondrial  [ATP5A1](http://www.pantherdb.org/genes/gene.do?acc=HUMAN%7CEnsembl=ENSG00000152234%7CUniProtKB=P25705)  [ortholog](http://www.pantherdb.org/genes/gene.do?acc=HUMAN%7CEnsembl=ENSG00000152234%7CUniProtKB=P25705#orthologs) | [respiratory electron transport chain](http://www.pantherdb.org/panther/category.do?categoryAcc=GO:0022904)  [purine nucleobase metabolic process](http://www.pantherdb.org/panther/category.do?categoryAcc=GO:0006144)  [cation transport](http://www.pantherdb.org/panther/category.do?categoryAcc=GO:0006812) |
| [HUMAN\|Ensembl=ENSG00000163918\|UniProtKB=P35249](http://www.pantherdb.org/genes/gene.do?acc=HUMAN%7CEnsembl=ENSG00000163918%7CUniProtKB=P35249) | P35249 | Replication factor C subunit 4  [RFC4](http://www.pantherdb.org/genes/gene.do?acc=HUMAN%7CEnsembl=ENSG00000163918%7CUniProtKB=P35249)  [ortholog](http://www.pantherdb.org/genes/gene.do?acc=HUMAN%7CEnsembl=ENSG00000163918%7CUniProtKB=P35249#orthologs) | [DNA replication](http://www.pantherdb.org/panther/category.do?categoryAcc=GO:0006260)  [cell cycle](http://www.pantherdb.org/panther/category.do?categoryAcc=GO:0007049) |
| [HUMAN\|Ensembl=ENSG00000014641\|UniProtKB=P40925](http://www.pantherdb.org/genes/gene.do?acc=HUMAN%7CEnsembl=ENSG00000014641%7CUniProtKB=P40925) | P40925 | Malate dehydrogenase, cytoplasmic  [MDH1](http://www.pantherdb.org/genes/gene.do?acc=HUMAN%7CEnsembl=ENSG00000014641%7CUniProtKB=P40925)  [ortholog](http://www.pantherdb.org/genes/gene.do?acc=HUMAN%7CEnsembl=ENSG00000014641%7CUniProtKB=P40925#orthologs) | [generation of precursor metabolites and energy](http://www.pantherdb.org/panther/category.do?categoryAcc=GO:0006091)  [carbohydrate metabolic process](http://www.pantherdb.org/panther/category.do?categoryAcc=GO:0005975)  [tricarboxylic acid cycle](http://www.pantherdb.org/panther/category.do?categoryAcc=GO:0006099) |
| [HUMAN\|Ensembl=ENSG00000109332\|UniProtKB=P61077](http://www.pantherdb.org/genes/gene.do?acc=HUMAN%7CEnsembl=ENSG00000109332%7CUniProtKB=P61077) | P61077 | Ubiquitin-conjugating enzyme E2 D3  [UBE2D3](http://www.pantherdb.org/genes/gene.do?acc=HUMAN%7CEnsembl=ENSG00000109332%7CUniProtKB=P61077)  [ortholog](http://www.pantherdb.org/genes/gene.do?acc=HUMAN%7CEnsembl=ENSG00000109332%7CUniProtKB=P61077#orthologs) | [apoptotic process](http://www.pantherdb.org/panther/category.do?categoryAcc=GO:0006915)  [cellular protein modification process](http://www.pantherdb.org/panther/category.do?categoryAcc=GO:0006464)  [apoptotic process](http://www.pantherdb.org/panther/category.do?categoryAcc=GO:0006915) |
| [HUMAN\|Ensembl=ENSG00000184640\|UniProtKB=Q9UHD8](http://www.pantherdb.org/genes/gene.do?acc=HUMAN%7CEnsembl=ENSG00000184640%7CUniProtKB=Q9UHD8) | Q9UHD8 | Septin-9  [ortholog](http://www.pantherdb.org/genes/gene.do?acc=HUMAN%7CEnsembl=ENSG00000184640%7CUniProtKB=Q9UHD8#orthologs) | [metabolic process](http://www.pantherdb.org/panther/category.do?categoryAcc=GO:0008152)  [cytokinesis](http://www.pantherdb.org/panther/category.do?categoryAcc=GO:0000910)  [mitosis](http://www.pantherdb.org/panther/category.do?categoryAcc=GO:0007067) |
| [HUMAN\|Ensembl=ENSG00000091164\|UniProtKB=O43396](http://www.pantherdb.org/genes/gene.do?acc=HUMAN%7CEnsembl=ENSG00000091164%7CUniProtKB=O43396) | O43396 | Thioredoxin-like protein 1  [TXNL1](http://www.pantherdb.org/genes/gene.do?acc=HUMAN%7CEnsembl=ENSG00000091164%7CUniProtKB=O43396)  [ortholog](http://www.pantherdb.org/genes/gene.do?acc=HUMAN%7CEnsembl=ENSG00000091164%7CUniProtKB=O43396#orthologs) | [respiratory electron transport chain](http://www.pantherdb.org/panther/category.do?categoryAcc=GO:0022904)  [sulfur compound metabolic process](http://www.pantherdb.org/panther/category.do?categoryAcc=GO:0006790)  [cell cycle](http://www.pantherdb.org/panther/category.do?categoryAcc=GO:0007049)  [cell communication](http://www.pantherdb.org/panther/category.do?categoryAcc=GO:0007154)  [response to stress](http://www.pantherdb.org/panther/category.do?categoryAcc=GO:0006950) |
| [HUMAN\|Ensembl=ENSG00000154473\|UniProtKB=O43684](http://www.pantherdb.org/genes/gene.do?acc=HUMAN%7CEnsembl=ENSG00000154473%7CUniProtKB=O43684) | O43684 | Mitotic checkpoint protein BUB3  [BUB3](http://www.pantherdb.org/genes/gene.do?acc=HUMAN%7CEnsembl=ENSG00000154473%7CUniProtKB=O43684)  [ortholog](http://www.pantherdb.org/genes/gene.do?acc=HUMAN%7CEnsembl=ENSG00000154473%7CUniProtKB=O43684#orthologs) | [nucleobase-containing compound metabolic process](http://www.pantherdb.org/panther/category.do?categoryAcc=GO:0006139)  [mitosis](http://www.pantherdb.org/panther/category.do?categoryAcc=GO:0007067)  [chromosome segregation](http://www.pantherdb.org/panther/category.do?categoryAcc=GO:0007059)  [RNA localization](http://www.pantherdb.org/panther/category.do?categoryAcc=GO:0006403) |
| [HUMAN\|Ensembl=ENSG00000163399\|UniProtKB=P05023](http://www.pantherdb.org/genes/gene.do?acc=HUMAN%7CEnsembl=ENSG00000163399%7CUniProtKB=P05023) | P05023 | Sodium/potassium-transporting ATPase subunit alpha-1  [ATP1A1](http://www.pantherdb.org/genes/gene.do?acc=HUMAN%7CEnsembl=ENSG00000163399%7CUniProtKB=P05023)  [ortholog](http://www.pantherdb.org/genes/gene.do?acc=HUMAN%7CEnsembl=ENSG00000163399%7CUniProtKB=P05023#orthologs) | [metabolic process](http://www.pantherdb.org/panther/category.do?categoryAcc=GO:0008152)  [cation transport](http://www.pantherdb.org/panther/category.do?categoryAcc=GO:0006812)  [cellular calcium ion homeostasis](http://www.pantherdb.org/panther/category.do?categoryAcc=GO:0006874) |
| [HUMAN\|Ensembl=ENSG00000105220\|UniProtKB=P06744](http://www.pantherdb.org/genes/gene.do?acc=HUMAN%7CEnsembl=ENSG00000105220%7CUniProtKB=P06744) | P06744 | Glucose-6-phosphate isomerase  [GPI](http://www.pantherdb.org/genes/gene.do?acc=HUMAN%7CEnsembl=ENSG00000105220%7CUniProtKB=P06744)  [ortholog](http://www.pantherdb.org/genes/gene.do?acc=HUMAN%7CEnsembl=ENSG00000105220%7CUniProtKB=P06744#orthologs) | [glycolysis](http://www.pantherdb.org/panther/category.do?categoryAcc=GO:0006096)  [gluconeogenesis](http://www.pantherdb.org/panther/category.do?categoryAcc=GO:0006094)  [glycolysis](http://www.pantherdb.org/panther/category.do?categoryAcc=GO:0006096) |
| [HUMAN\|Ensembl=ENSG00000080824\|UniProtKB=P07900](http://www.pantherdb.org/genes/gene.do?acc=HUMAN%7CEnsembl=ENSG00000080824%7CUniProtKB=P07900) | P07900 | Heat shock protein HSP 90-alpha  [HSP90AA1](http://www.pantherdb.org/genes/gene.do?acc=HUMAN%7CEnsembl=ENSG00000080824%7CUniProtKB=P07900)  [ortholog](http://www.pantherdb.org/genes/gene.do?acc=HUMAN%7CEnsembl=ENSG00000080824%7CUniProtKB=P07900#orthologs) | [protein folding](http://www.pantherdb.org/panther/category.do?categoryAcc=GO:0006457)  [response to stress](http://www.pantherdb.org/panther/category.do?categoryAcc=GO:0006950) |
| [HUMAN\|Ensembl=ENSG00000096384\|UniProtKB=P08238](http://www.pantherdb.org/genes/gene.do?acc=HUMAN%7CEnsembl=ENSG00000096384%7CUniProtKB=P08238) | P08238 | Heat shock protein HSP 90-beta  [HSP90AB1](http://www.pantherdb.org/genes/gene.do?acc=HUMAN%7CEnsembl=ENSG00000096384%7CUniProtKB=P08238)  [ortholog](http://www.pantherdb.org/genes/gene.do?acc=HUMAN%7CEnsembl=ENSG00000096384%7CUniProtKB=P08238#orthologs) | [immune system process](http://www.pantherdb.org/panther/category.do?categoryAcc=GO:0002376)  [protein folding](http://www.pantherdb.org/panther/category.do?categoryAcc=GO:0006457)  [response to stress](http://www.pantherdb.org/panther/category.do?categoryAcc=GO:0006950) |
| [HUMAN\|Ensembl=ENSG00000164111\|UniProtKB=P08758](http://www.pantherdb.org/genes/gene.do?acc=HUMAN%7CEnsembl=ENSG00000164111%7CUniProtKB=P08758) | P08758 | Annexin A5  [ANXA5](http://www.pantherdb.org/genes/gene.do?acc=HUMAN%7CEnsembl=ENSG00000164111%7CUniProtKB=P08758)  [ortholog](http://www.pantherdb.org/genes/gene.do?acc=HUMAN%7CEnsembl=ENSG00000164111%7CUniProtKB=P08758#orthologs) | [fatty acid metabolic process](http://www.pantherdb.org/panther/category.do?categoryAcc=GO:0006631) |
| [HUMAN\|Ensembl=ENSG00000100567\|UniProtKB=P25788](http://www.pantherdb.org/genes/gene.do?acc=HUMAN%7CEnsembl=ENSG00000100567%7CUniProtKB=P25788) | P25788 | Proteasome subunit alpha type-3  [PSMA3](http://www.pantherdb.org/genes/gene.do?acc=HUMAN%7CEnsembl=ENSG00000100567%7CUniProtKB=P25788)  [ortholog](http://www.pantherdb.org/genes/gene.do?acc=HUMAN%7CEnsembl=ENSG00000100567%7CUniProtKB=P25788#orthologs) | [proteolysis](http://www.pantherdb.org/panther/category.do?categoryAcc=GO:0006508) |
| [HUMAN\|Ensembl=ENSG00000254772\|UniProtKB=P26641](http://www.pantherdb.org/genes/gene.do?acc=HUMAN%7CEnsembl=ENSG00000254772%7CUniProtKB=P26641) | P26641 | Elongation factor 1-gamma  [EEF1G](http://www.pantherdb.org/genes/gene.do?acc=HUMAN%7CEnsembl=ENSG00000254772%7CUniProtKB=P26641)  [ortholog](http://www.pantherdb.org/genes/gene.do?acc=HUMAN%7CEnsembl=ENSG00000254772%7CUniProtKB=P26641#orthologs) | [immune system process](http://www.pantherdb.org/panther/category.do?categoryAcc=GO:0002376)  [translation](http://www.pantherdb.org/panther/category.do?categoryAcc=GO:0006412)  [cell communication](http://www.pantherdb.org/panther/category.do?categoryAcc=GO:0007154)  [response to toxic substance](http://www.pantherdb.org/panther/category.do?categoryAcc=GO:0009636)  [regulation of translation](http://www.pantherdb.org/panther/category.do?categoryAcc=GO:0006417) |
| [HUMAN\|Ensembl=ENSG00000143106\|UniProtKB=P28066](http://www.pantherdb.org/genes/gene.do?acc=HUMAN%7CEnsembl=ENSG00000143106%7CUniProtKB=P28066) | P28066 | Proteasome subunit alpha type-5  [PSMA5](http://www.pantherdb.org/genes/gene.do?acc=HUMAN%7CEnsembl=ENSG00000143106%7CUniProtKB=P28066)  [ortholog](http://www.pantherdb.org/genes/gene.do?acc=HUMAN%7CEnsembl=ENSG00000143106%7CUniProtKB=P28066#orthologs) | [proteolysis](http://www.pantherdb.org/panther/category.do?categoryAcc=GO:0006508) |
| [HUMAN\|Ensembl=ENSG00000100804\|UniProtKB=P28074](http://www.pantherdb.org/genes/gene.do?acc=HUMAN%7CEnsembl=ENSG00000100804%7CUniProtKB=P28074) | P28074 | Proteasome subunit beta type-5  [PSMB5](http://www.pantherdb.org/genes/gene.do?acc=HUMAN%7CEnsembl=ENSG00000100804%7CUniProtKB=P28074)  [ortholog](http://www.pantherdb.org/genes/gene.do?acc=HUMAN%7CEnsembl=ENSG00000100804%7CUniProtKB=P28074#orthologs) | [proteolysis](http://www.pantherdb.org/panther/category.do?categoryAcc=GO:0006508) |
| [HUMAN\|Ensembl=ENSG00000169710\|UniProtKB=P49327](http://www.pantherdb.org/genes/gene.do?acc=HUMAN%7CEnsembl=ENSG00000169710%7CUniProtKB=P49327) | P49327 | Fatty acid synthase  [FASN](http://www.pantherdb.org/genes/gene.do?acc=HUMAN%7CEnsembl=ENSG00000169710%7CUniProtKB=P49327)  [ortholog](http://www.pantherdb.org/genes/gene.do?acc=HUMAN%7CEnsembl=ENSG00000169710%7CUniProtKB=P49327#orthologs) | [cellular amino acid metabolic process](http://www.pantherdb.org/panther/category.do?categoryAcc=GO:0006520)  [fatty acid biosynthetic process](http://www.pantherdb.org/panther/category.do?categoryAcc=GO:0006633) |
| [HUMAN\|Ensembl=ENSG00000178952\|UniProtKB=P49411](http://www.pantherdb.org/genes/gene.do?acc=HUMAN%7CEnsembl=ENSG00000178952%7CUniProtKB=P49411) | P49411 | Elongation factor Tu, mitochondrial  [TUFM](http://www.pantherdb.org/genes/gene.do?acc=HUMAN%7CEnsembl=ENSG00000178952%7CUniProtKB=P49411)  [ortholog](http://www.pantherdb.org/genes/gene.do?acc=HUMAN%7CEnsembl=ENSG00000178952%7CUniProtKB=P49411#orthologs) | [translation](http://www.pantherdb.org/panther/category.do?categoryAcc=GO:0006412)  [regulation of translation](http://www.pantherdb.org/panther/category.do?categoryAcc=GO:0006417) |
| [HUMAN\|Ensembl=ENSG00000090861\|UniProtKB=P49588](http://www.pantherdb.org/genes/gene.do?acc=HUMAN%7CEnsembl=ENSG00000090861%7CUniProtKB=P49588) | P49588 | Alanine--tRNA ligase, cytoplasmic  [AARS](http://www.pantherdb.org/genes/gene.do?acc=HUMAN%7CEnsembl=ENSG00000090861%7CUniProtKB=P49588)  [ortholog](http://www.pantherdb.org/genes/gene.do?acc=HUMAN%7CEnsembl=ENSG00000090861%7CUniProtKB=P49588#orthologs) | [tRNA metabolic process](http://www.pantherdb.org/panther/category.do?categoryAcc=GO:0006399) |
| [HUMAN\|Ensembl=ENSG00000115484\|UniProtKB=P50991](http://www.pantherdb.org/genes/gene.do?acc=HUMAN%7CEnsembl=ENSG00000115484%7CUniProtKB=P50991) | P50991 | T-complex protein 1 subunit delta  [CCT4](http://www.pantherdb.org/genes/gene.do?acc=HUMAN%7CEnsembl=ENSG00000115484%7CUniProtKB=P50991)  [ortholog](http://www.pantherdb.org/genes/gene.do?acc=HUMAN%7CEnsembl=ENSG00000115484%7CUniProtKB=P50991#orthologs) | [protein folding](http://www.pantherdb.org/panther/category.do?categoryAcc=GO:0006457)  [protein complex assembly](http://www.pantherdb.org/panther/category.do?categoryAcc=GO:0006461)  [protein complex biogenesis](http://www.pantherdb.org/panther/category.do?categoryAcc=GO:0070271) |
| [HUMAN\|Ensembl=ENSG00000260245\|UniProtKB=P53396](http://www.pantherdb.org/genes/gene.do?acc=HUMAN%7CEnsembl=ENSG00000260245%7CUniProtKB=P53396) | P53396 | ATP-citrate synthase  [ACLY](http://www.pantherdb.org/genes/gene.do?acc=HUMAN%7CEnsembl=ENSG00000260245%7CUniProtKB=P53396)  [ortholog](http://www.pantherdb.org/genes/gene.do?acc=HUMAN%7CEnsembl=ENSG00000260245%7CUniProtKB=P53396#orthologs) | [generation of precursor metabolites and energy](http://www.pantherdb.org/panther/category.do?categoryAcc=GO:0006091)  [coenzyme metabolic process](http://www.pantherdb.org/panther/category.do?categoryAcc=GO:0006732)  [carbohydrate metabolic process](http://www.pantherdb.org/panther/category.do?categoryAcc=GO:0005975)  [tricarboxylic acid cycle](http://www.pantherdb.org/panther/category.do?categoryAcc=GO:0006099)  [lipid metabolic process](http://www.pantherdb.org/panther/category.do?categoryAcc=GO:0006629) |
| [HUMAN\|Ensembl=ENSG00000124207\|UniProtKB=P55060](http://www.pantherdb.org/genes/gene.do?acc=HUMAN%7CEnsembl=ENSG00000124207%7CUniProtKB=P55060) | P55060 | Exportin-2  [CSE1L](http://www.pantherdb.org/genes/gene.do?acc=HUMAN%7CEnsembl=ENSG00000124207%7CUniProtKB=P55060)  [ortholog](http://www.pantherdb.org/genes/gene.do?acc=HUMAN%7CEnsembl=ENSG00000124207%7CUniProtKB=P55060#orthologs) | [metabolic process](http://www.pantherdb.org/panther/category.do?categoryAcc=GO:0008152)  [intracellular protein transport](http://www.pantherdb.org/panther/category.do?categoryAcc=GO:0006886)  [nuclear transport](http://www.pantherdb.org/panther/category.do?categoryAcc=GO:0051169) |
| [HUMAN\|Ensembl=ENSG00000136026\|UniProtKB=Q07065](http://www.pantherdb.org/genes/gene.do?acc=HUMAN%7CEnsembl=ENSG00000136026%7CUniProtKB=Q07065) | Q07065 | Cytoskeleton-associated protein 4  [CKAP4](http://www.pantherdb.org/genes/gene.do?acc=HUMAN%7CEnsembl=ENSG00000136026%7CUniProtKB=Q07065)  [ortholog](http://www.pantherdb.org/genes/gene.do?acc=HUMAN%7CEnsembl=ENSG00000136026%7CUniProtKB=Q07065#orthologs) | [DNA replication](http://www.pantherdb.org/panther/category.do?categoryAcc=GO:0006260)  [DNA repair](http://www.pantherdb.org/panther/category.do?categoryAcc=GO:0006281)  [mitosis](http://www.pantherdb.org/panther/category.do?categoryAcc=GO:0007067)  [meiosis](http://www.pantherdb.org/panther/category.do?categoryAcc=GO:0007126)  [chromosome segregation](http://www.pantherdb.org/panther/category.do?categoryAcc=GO:0007059)  [chromatin organization](http://www.pantherdb.org/panther/category.do?categoryAcc=GO:0006325) |
| [HUMAN\|Ensembl=ENSG00000187840\|UniProtKB=Q13541](http://www.pantherdb.org/genes/gene.do?acc=HUMAN%7CEnsembl=ENSG00000187840%7CUniProtKB=Q13541) | Q13541 | Eukaryotic translation initiation factor 4E-binding protein 1  [EIF4EBP1](http://www.pantherdb.org/genes/gene.do?acc=HUMAN%7CEnsembl=ENSG00000187840%7CUniProtKB=Q13541)  [ortholog](http://www.pantherdb.org/genes/gene.do?acc=HUMAN%7CEnsembl=ENSG00000187840%7CUniProtKB=Q13541#orthologs) | [translation](http://www.pantherdb.org/panther/category.do?categoryAcc=GO:0006412) |
| [HUMAN\|Ensembl=ENSG00000263344\|UniProtKB=Q15056](http://www.pantherdb.org/genes/gene.do?acc=HUMAN%7CEnsembl=ENSG00000263344%7CUniProtKB=Q15056) | Q15056 | Eukaryotic translation initiation factor 4H  [EIF4H](http://www.pantherdb.org/genes/gene.do?acc=HUMAN%7CEnsembl=ENSG00000263344%7CUniProtKB=Q15056)  [ortholog](http://www.pantherdb.org/genes/gene.do?acc=HUMAN%7CEnsembl=ENSG00000263344%7CUniProtKB=Q15056#orthologs) | [translation](http://www.pantherdb.org/panther/category.do?categoryAcc=GO:0006412)  [regulation of translation](http://www.pantherdb.org/panther/category.do?categoryAcc=GO:0006417) |
| [HUMAN\|Ensembl=ENSG00000143870\|UniProtKB=Q15084](http://www.pantherdb.org/genes/gene.do?acc=HUMAN%7CEnsembl=ENSG00000143870%7CUniProtKB=Q15084) | Q15084 | Protein disulfide-isomerase A6  [PDIA6](http://www.pantherdb.org/genes/gene.do?acc=HUMAN%7CEnsembl=ENSG00000143870%7CUniProtKB=Q15084)  [ortholog](http://www.pantherdb.org/genes/gene.do?acc=HUMAN%7CEnsembl=ENSG00000143870%7CUniProtKB=Q15084#orthologs) | [protein folding](http://www.pantherdb.org/panther/category.do?categoryAcc=GO:0006457)  [cellular protein modification process](http://www.pantherdb.org/panther/category.do?categoryAcc=GO:0006464) |
| [HUMAN\|Ensembl=ENSG00000262812\|UniProtKB=Q9Y4L1](http://www.pantherdb.org/genes/gene.do?acc=HUMAN%7CEnsembl=ENSG00000262812%7CUniProtKB=Q9Y4L1) | Q9Y4L1 | Hypoxia up-regulated protein 1  [HYOU1](http://www.pantherdb.org/genes/gene.do?acc=HUMAN%7CEnsembl=ENSG00000262812%7CUniProtKB=Q9Y4L1)  [ortholog](http://www.pantherdb.org/genes/gene.do?acc=HUMAN%7CEnsembl=ENSG00000262812%7CUniProtKB=Q9Y4L1#orthologs) | [protein folding](http://www.pantherdb.org/panther/category.do?categoryAcc=GO:0006457)  [protein complex assembly](http://www.pantherdb.org/panther/category.do?categoryAcc=GO:0006461)  [response to stress](http://www.pantherdb.org/panther/category.do?categoryAcc=GO:0006950)  [protein complex biogenesis](http://www.pantherdb.org/panther/category.do?categoryAcc=GO:0070271) |
| [HUMAN\|Ensembl=ENSG00000223639\|UniProtKB=O00299](http://www.pantherdb.org/genes/gene.do?acc=HUMAN%7CEnsembl=ENSG00000223639%7CUniProtKB=O00299) | O00299 | Chloride intracellular channel protein 1  [CLIC1](http://www.pantherdb.org/genes/gene.do?acc=HUMAN%7CEnsembl=ENSG00000223639%7CUniProtKB=O00299)  [ortholog](http://www.pantherdb.org/genes/gene.do?acc=HUMAN%7CEnsembl=ENSG00000223639%7CUniProtKB=O00299#orthologs) | [immune system process](http://www.pantherdb.org/panther/category.do?categoryAcc=GO:0002376)  [translation](http://www.pantherdb.org/panther/category.do?categoryAcc=GO:0006412)  [cell communication](http://www.pantherdb.org/panther/category.do?categoryAcc=GO:0007154)  [response to toxic substance](http://www.pantherdb.org/panther/category.do?categoryAcc=GO:0009636)  [anion transport](http://www.pantherdb.org/panther/category.do?categoryAcc=GO:0006820)  [regulation of translation](http://www.pantherdb.org/panther/category.do?categoryAcc=GO:0006417) |
| [HUMAN\|Ensembl=ENSG00000136628\|UniProtKB=P07814](http://www.pantherdb.org/genes/gene.do?acc=HUMAN%7CEnsembl=ENSG00000136628%7CUniProtKB=P07814) | P07814 | Bifunctional glutamate/proline--tRNA ligase  [EPRS](http://www.pantherdb.org/genes/gene.do?acc=HUMAN%7CEnsembl=ENSG00000136628%7CUniProtKB=P07814)  [ortholog](http://www.pantherdb.org/genes/gene.do?acc=HUMAN%7CEnsembl=ENSG00000136628%7CUniProtKB=P07814#orthologs) | [translation](http://www.pantherdb.org/panther/category.do?categoryAcc=GO:0006412) |
| [HUMAN\|Ensembl=ENSG00000160752\|UniProtKB=P14324](http://www.pantherdb.org/genes/gene.do?acc=HUMAN%7CEnsembl=ENSG00000160752%7CUniProtKB=P14324) | P14324 | Farnesyl pyrophosphate synthase  [FDPS](http://www.pantherdb.org/genes/gene.do?acc=HUMAN%7CEnsembl=ENSG00000160752%7CUniProtKB=P14324)  [ortholog](http://www.pantherdb.org/genes/gene.do?acc=HUMAN%7CEnsembl=ENSG00000160752%7CUniProtKB=P14324#orthologs) | [cholesterol metabolic process](http://www.pantherdb.org/panther/category.do?categoryAcc=GO:0008203) |
| [HUMAN\|Ensembl=ENSG00000166825\|UniProtKB=P15144](http://www.pantherdb.org/genes/gene.do?acc=HUMAN%7CEnsembl=ENSG00000166825%7CUniProtKB=P15144) | P15144 | Aminopeptidase N  [ANPEP](http://www.pantherdb.org/genes/gene.do?acc=HUMAN%7CEnsembl=ENSG00000166825%7CUniProtKB=P15144)  [ortholog](http://www.pantherdb.org/genes/gene.do?acc=HUMAN%7CEnsembl=ENSG00000166825%7CUniProtKB=P15144#orthologs) | [proteolysis](http://www.pantherdb.org/panther/category.do?categoryAcc=GO:0006508) |
| [HUMAN\|Ensembl=ENSG00000153113\|UniProtKB=P20810](http://www.pantherdb.org/genes/gene.do?acc=HUMAN%7CEnsembl=ENSG00000153113%7CUniProtKB=P20810) | P20810 | Calpastatin  [CAST](http://www.pantherdb.org/genes/gene.do?acc=HUMAN%7CEnsembl=ENSG00000153113%7CUniProtKB=P20810)  [ortholog](http://www.pantherdb.org/genes/gene.do?acc=HUMAN%7CEnsembl=ENSG00000153113%7CUniProtKB=P20810#orthologs) | [proteolysis](http://www.pantherdb.org/panther/category.do?categoryAcc=GO:0006508)  [regulation of catalytic activity](http://www.pantherdb.org/panther/category.do?categoryAcc=GO:0050790) |
| [HUMAN\|Ensembl=ENSG00000117592\|UniProtKB=P30041](http://www.pantherdb.org/genes/gene.do?acc=HUMAN%7CEnsembl=ENSG00000117592%7CUniProtKB=P30041) | P30041 | Peroxiredoxin-6  [PRDX6](http://www.pantherdb.org/genes/gene.do?acc=HUMAN%7CEnsembl=ENSG00000117592%7CUniProtKB=P30041)  [ortholog](http://www.pantherdb.org/genes/gene.do?acc=HUMAN%7CEnsembl=ENSG00000117592%7CUniProtKB=P30041#orthologs) | [metabolic process](http://www.pantherdb.org/panther/category.do?categoryAcc=GO:0008152) |
| [HUMAN\|Ensembl=ENSG00000156261\|UniProtKB=P50990](http://www.pantherdb.org/genes/gene.do?acc=HUMAN%7CEnsembl=ENSG00000156261%7CUniProtKB=P50990) | P50990 | T-complex protein 1 subunit theta  [CCT8](http://www.pantherdb.org/genes/gene.do?acc=HUMAN%7CEnsembl=ENSG00000156261%7CUniProtKB=P50990)  [ortholog](http://www.pantherdb.org/genes/gene.do?acc=HUMAN%7CEnsembl=ENSG00000156261%7CUniProtKB=P50990#orthologs) | [protein folding](http://www.pantherdb.org/panther/category.do?categoryAcc=GO:0006457)  [protein complex assembly](http://www.pantherdb.org/panther/category.do?categoryAcc=GO:0006461)  [protein complex biogenesis](http://www.pantherdb.org/panther/category.do?categoryAcc=GO:0070271) |
| [HUMAN\|Ensembl=ENSG00000172531\|UniProtKB=P62136](http://www.pantherdb.org/genes/gene.do?acc=HUMAN%7CEnsembl=ENSG00000172531%7CUniProtKB=P62136) | P62136 | Serine/threonine-protein phosphatase PP1-alpha catalytic subunit  [PPP1CA](http://www.pantherdb.org/genes/gene.do?acc=HUMAN%7CEnsembl=ENSG00000172531%7CUniProtKB=P62136)  [ortholog](http://www.pantherdb.org/genes/gene.do?acc=HUMAN%7CEnsembl=ENSG00000172531%7CUniProtKB=P62136#orthologs) | [immune system process](http://www.pantherdb.org/panther/category.do?categoryAcc=GO:0002376)  [apoptotic process](http://www.pantherdb.org/panther/category.do?categoryAcc=GO:0006915)  [glycogen metabolic process](http://www.pantherdb.org/panther/category.do?categoryAcc=GO:0005977)  [transcription from RNA polymerase II promoter](http://www.pantherdb.org/panther/category.do?categoryAcc=GO:0006366)  [mRNA processing](http://www.pantherdb.org/panther/category.do?categoryAcc=GO:0006397)  [protein phosphorylation](http://www.pantherdb.org/panther/category.do?categoryAcc=GO:0006468)  [mitosis](http://www.pantherdb.org/panther/category.do?categoryAcc=GO:0007067)  [meiosis](http://www.pantherdb.org/panther/category.do?categoryAcc=GO:0007126)  [cell communication](http://www.pantherdb.org/panther/category.do?categoryAcc=GO:0007154)  [apoptotic process](http://www.pantherdb.org/panther/category.do?categoryAcc=GO:0006915)  [response to stress](http://www.pantherdb.org/panther/category.do?categoryAcc=GO:0006950)  [regulation of carbohydrate metabolic process](http://www.pantherdb.org/panther/category.do?categoryAcc=GO:0006109)  [regulation of nucleobase-containing compound metabolic process](http://www.pantherdb.org/panther/category.do?categoryAcc=GO:0019219) |
|  |  |  |  |
|  |  |  |  |
| [HUMAN\|Ensembl=ENSG00000196262\|UniProtKB=P62937](http://www.pantherdb.org/genes/gene.do?acc=HUMAN%7CEnsembl=ENSG00000196262%7CUniProtKB=P62937) | P62937 | Peptidyl-prolyl cis-trans isomerase A  [PPIA](http://www.pantherdb.org/genes/gene.do?acc=HUMAN%7CEnsembl=ENSG00000196262%7CUniProtKB=P62937)  [ortholog](http://www.pantherdb.org/genes/gene.do?acc=HUMAN%7CEnsembl=ENSG00000196262%7CUniProtKB=P62937#orthologs) | [immune system process](http://www.pantherdb.org/panther/category.do?categoryAcc=GO:0002376)  [protein folding](http://www.pantherdb.org/panther/category.do?categoryAcc=GO:0006457)  [intracellular protein transport](http://www.pantherdb.org/panther/category.do?categoryAcc=GO:0006886)  [nuclear transport](http://www.pantherdb.org/panther/category.do?categoryAcc=GO:0051169) |
| [HUMAN\|Ensembl=ENSG00000135624\|UniProtKB=Q99832](http://www.pantherdb.org/genes/gene.do?acc=HUMAN%7CEnsembl=ENSG00000135624%7CUniProtKB=Q99832) | Q99832 | T-complex protein 1 subunit eta  [CCT7](http://www.pantherdb.org/genes/gene.do?acc=HUMAN%7CEnsembl=ENSG00000135624%7CUniProtKB=Q99832)  [ortholog](http://www.pantherdb.org/genes/gene.do?acc=HUMAN%7CEnsembl=ENSG00000135624%7CUniProtKB=Q99832#orthologs) | [protein folding](http://www.pantherdb.org/panther/category.do?categoryAcc=GO:0006457)  [protein complex assembly](http://www.pantherdb.org/panther/category.do?categoryAcc=GO:0006461)  [protein complex biogenesis](http://www.pantherdb.org/panther/category.do?categoryAcc=GO:0070271) |
| [HUMAN\|Ensembl=ENSG00000116161\|UniProtKB=Q9HB71](http://www.pantherdb.org/genes/gene.do?acc=HUMAN%7CEnsembl=ENSG00000116161%7CUniProtKB=Q9HB71) | Q9HB71 | Calcyclin-binding protein  [CACYBP](http://www.pantherdb.org/genes/gene.do?acc=HUMAN%7CEnsembl=ENSG00000116161%7CUniProtKB=Q9HB71)  [ortholog](http://www.pantherdb.org/genes/gene.do?acc=HUMAN%7CEnsembl=ENSG00000116161%7CUniProtKB=Q9HB71#orthologs) | [proteolysis](http://www.pantherdb.org/panther/category.do?categoryAcc=GO:0006508) |
| [HUMAN\|Ensembl=ENSG00000178896\|UniProtKB=Q9NPD3](http://www.pantherdb.org/genes/gene.do?acc=HUMAN%7CEnsembl=ENSG00000178896%7CUniProtKB=Q9NPD3) | Q9NPD3 | Exosome complex component RRP41  [EXOSC4](http://www.pantherdb.org/genes/gene.do?acc=HUMAN%7CEnsembl=ENSG00000178896%7CUniProtKB=Q9NPD3)  [ortholog](http://www.pantherdb.org/genes/gene.do?acc=HUMAN%7CEnsembl=ENSG00000178896%7CUniProtKB=Q9NPD3#orthologs) | [tRNA metabolic process](http://www.pantherdb.org/panther/category.do?categoryAcc=GO:0006399)  [RNA catabolic process](http://www.pantherdb.org/panther/category.do?categoryAcc=GO:0006401)  [rRNA metabolic process](http://www.pantherdb.org/panther/category.do?categoryAcc=GO:0016072) |
| [HUMAN\|Ensembl=ENSG00000092621\|UniProtKB=O43175](http://www.pantherdb.org/genes/gene.do?acc=HUMAN%7CEnsembl=ENSG00000092621%7CUniProtKB=O43175) | O43175 | D-3-phosphoglycerate dehydrogenase  [PHGDH](http://www.pantherdb.org/genes/gene.do?acc=HUMAN%7CEnsembl=ENSG00000092621%7CUniProtKB=O43175)  [ortholog](http://www.pantherdb.org/genes/gene.do?acc=HUMAN%7CEnsembl=ENSG00000092621%7CUniProtKB=O43175#orthologs) | [carbohydrate metabolic process](http://www.pantherdb.org/panther/category.do?categoryAcc=GO:0005975)  [cellular amino acid biosynthetic process](http://www.pantherdb.org/panther/category.do?categoryAcc=GO:0008652) |
| [HUMAN\|Ensembl=ENSG00000110955\|UniProtKB=P06576](http://www.pantherdb.org/genes/gene.do?acc=HUMAN%7CEnsembl=ENSG00000110955%7CUniProtKB=P06576) | P06576 | ATP synthase subunit beta, mitochondrial  [ATP5B](http://www.pantherdb.org/genes/gene.do?acc=HUMAN%7CEnsembl=ENSG00000110955%7CUniProtKB=P06576)  [ortholog](http://www.pantherdb.org/genes/gene.do?acc=HUMAN%7CEnsembl=ENSG00000110955%7CUniProtKB=P06576#orthologs) | [respiratory electron transport chain](http://www.pantherdb.org/panther/category.do?categoryAcc=GO:0022904)  [purine nucleobase metabolic process](http://www.pantherdb.org/panther/category.do?categoryAcc=GO:0006144)  [cation transport](http://www.pantherdb.org/panther/category.do?categoryAcc=GO:0006812) |
| [HUMAN\|Ensembl=ENSG00000170445\|UniProtKB=P12081](http://www.pantherdb.org/genes/gene.do?acc=HUMAN%7CEnsembl=ENSG00000170445%7CUniProtKB=P12081) | P12081 | Histidine--tRNA ligase, cytoplasmic  [HARS](http://www.pantherdb.org/genes/gene.do?acc=HUMAN%7CEnsembl=ENSG00000170445%7CUniProtKB=P12081)  [ortholog](http://www.pantherdb.org/genes/gene.do?acc=HUMAN%7CEnsembl=ENSG00000170445%7CUniProtKB=P12081#orthologs) | [translation](http://www.pantherdb.org/panther/category.do?categoryAcc=GO:0006412) |
| [HUMAN\|Ensembl=ENSG00000166598\|UniProtKB=P14625](http://www.pantherdb.org/genes/gene.do?acc=HUMAN%7CEnsembl=ENSG00000166598%7CUniProtKB=P14625) | P14625 | Endoplasmin  [HSP90B1](http://www.pantherdb.org/genes/gene.do?acc=HUMAN%7CEnsembl=ENSG00000166598%7CUniProtKB=P14625)  [ortholog](http://www.pantherdb.org/genes/gene.do?acc=HUMAN%7CEnsembl=ENSG00000166598%7CUniProtKB=P14625#orthologs) | [protein folding](http://www.pantherdb.org/panther/category.do?categoryAcc=GO:0006457)  [response to stress](http://www.pantherdb.org/panther/category.do?categoryAcc=GO:0006950) |
| [HUMAN\|Ensembl=ENSG00000168374\|UniProtKB=P18085](http://www.pantherdb.org/genes/gene.do?acc=HUMAN%7CEnsembl=ENSG00000168374%7CUniProtKB=P18085) | P18085 | ADP-ribosylation factor 4  [ARF4](http://www.pantherdb.org/genes/gene.do?acc=HUMAN%7CEnsembl=ENSG00000168374%7CUniProtKB=P18085)  [ortholog](http://www.pantherdb.org/genes/gene.do?acc=HUMAN%7CEnsembl=ENSG00000168374%7CUniProtKB=P18085#orthologs) | [metabolic process](http://www.pantherdb.org/panther/category.do?categoryAcc=GO:0008152)  [cell communication](http://www.pantherdb.org/panther/category.do?categoryAcc=GO:0007154)  [intracellular protein transport](http://www.pantherdb.org/panther/category.do?categoryAcc=GO:0006886)  [vesicle-mediated transport](http://www.pantherdb.org/panther/category.do?categoryAcc=GO:0016192) |
| [HUMAN\|Ensembl=ENSG00000159335\|UniProtKB=P20962](http://www.pantherdb.org/genes/gene.do?acc=HUMAN%7CEnsembl=ENSG00000159335%7CUniProtKB=P20962) | P20962 | Parathymosin  [PTMS](http://www.pantherdb.org/genes/gene.do?acc=HUMAN%7CEnsembl=ENSG00000159335%7CUniProtKB=P20962)  [ortholog](http://www.pantherdb.org/genes/gene.do?acc=HUMAN%7CEnsembl=ENSG00000159335%7CUniProtKB=P20962#orthologs) | [nucleobase-containing compound metabolic process](http://www.pantherdb.org/panther/category.do?categoryAcc=GO:0006139) |
| [HUMAN\|Ensembl=ENSG00000226589\|UniProtKB=P26640](http://www.pantherdb.org/genes/gene.do?acc=HUMAN%7CEnsembl=ENSG00000226589%7CUniProtKB=P26640) | P26640 | Valine--tRNA ligase  [VARS](http://www.pantherdb.org/genes/gene.do?acc=HUMAN%7CEnsembl=ENSG00000226589%7CUniProtKB=P26640)  [ortholog](http://www.pantherdb.org/genes/gene.do?acc=HUMAN%7CEnsembl=ENSG00000226589%7CUniProtKB=P26640#orthologs) | [translation](http://www.pantherdb.org/panther/category.do?categoryAcc=GO:0006412) |
| [HUMAN\|Ensembl=ENSG00000146731\|UniProtKB=P40227](http://www.pantherdb.org/genes/gene.do?acc=HUMAN%7CEnsembl=ENSG00000146731%7CUniProtKB=P40227) | P40227 | T-complex protein 1 subunit zeta  [CCT6A](http://www.pantherdb.org/genes/gene.do?acc=HUMAN%7CEnsembl=ENSG00000146731%7CUniProtKB=P40227)  [ortholog](http://www.pantherdb.org/genes/gene.do?acc=HUMAN%7CEnsembl=ENSG00000146731%7CUniProtKB=P40227#orthologs) | [protein folding](http://www.pantherdb.org/panther/category.do?categoryAcc=GO:0006457)  [protein complex assembly](http://www.pantherdb.org/panther/category.do?categoryAcc=GO:0006461)  [protein complex biogenesis](http://www.pantherdb.org/panther/category.do?categoryAcc=GO:0070271) |
| [HUMAN\|Ensembl=ENSG00000142657\|UniProtKB=P52209](http://www.pantherdb.org/genes/gene.do?acc=HUMAN%7CEnsembl=ENSG00000142657%7CUniProtKB=P52209) | P52209 | 6-phosphogluconate dehydrogenase, decarboxylating  [PGD](http://www.pantherdb.org/genes/gene.do?acc=HUMAN%7CEnsembl=ENSG00000142657%7CUniProtKB=P52209)  [ortholog](http://www.pantherdb.org/genes/gene.do?acc=HUMAN%7CEnsembl=ENSG00000142657%7CUniProtKB=P52209#orthologs) | [pentose-phosphate shunt](http://www.pantherdb.org/panther/category.do?categoryAcc=GO:0006098) |
| [HUMAN\|Ensembl=ENSG00000087191\|UniProtKB=P62195](http://www.pantherdb.org/genes/gene.do?acc=HUMAN%7CEnsembl=ENSG00000087191%7CUniProtKB=P62195) | P62195 | 26S protease regulatory subunit 8  [PSMC5](http://www.pantherdb.org/genes/gene.do?acc=HUMAN%7CEnsembl=ENSG00000087191%7CUniProtKB=P62195)  [ortholog](http://www.pantherdb.org/genes/gene.do?acc=HUMAN%7CEnsembl=ENSG00000087191%7CUniProtKB=P62195#orthologs) | [proteolysis](http://www.pantherdb.org/panther/category.do?categoryAcc=GO:0006508) |
| [HUMAN\|Ensembl=ENSG00000088832\|UniProtKB=P62942](http://www.pantherdb.org/genes/gene.do?acc=HUMAN%7CEnsembl=ENSG00000088832%7CUniProtKB=P62942) | P62942 | Peptidyl-prolyl cis-trans isomerase FKBP1A  [FKBP1A](http://www.pantherdb.org/genes/gene.do?acc=HUMAN%7CEnsembl=ENSG00000088832%7CUniProtKB=P62942)  [ortholog](http://www.pantherdb.org/genes/gene.do?acc=HUMAN%7CEnsembl=ENSG00000088832%7CUniProtKB=P62942#orthologs) | [cellular protein modification process](http://www.pantherdb.org/panther/category.do?categoryAcc=GO:0006464)  [cellular process](http://www.pantherdb.org/panther/category.do?categoryAcc=GO:0009987) |
| [HUMAN\|Ensembl=ENSG00000092010\|UniProtKB=Q06323](http://www.pantherdb.org/genes/gene.do?acc=HUMAN%7CEnsembl=ENSG00000092010%7CUniProtKB=Q06323) | Q06323 | Proteasome activator complex subunit 1  [PSME1](http://www.pantherdb.org/genes/gene.do?acc=HUMAN%7CEnsembl=ENSG00000092010%7CUniProtKB=Q06323)  [ortholog](http://www.pantherdb.org/genes/gene.do?acc=HUMAN%7CEnsembl=ENSG00000092010%7CUniProtKB=Q06323#orthologs) | [proteolysis](http://www.pantherdb.org/panther/category.do?categoryAcc=GO:0006508) |
| [HUMAN\|Ensembl=ENSG00000099810\|UniProtKB=Q13126](http://www.pantherdb.org/genes/gene.do?acc=HUMAN%7CEnsembl=ENSG00000099810%7CUniProtKB=Q13126) | Q13126 | S-methyl-5'-thioadenosine phosphorylase  [MTAP](http://www.pantherdb.org/genes/gene.do?acc=HUMAN%7CEnsembl=ENSG00000099810%7CUniProtKB=Q13126)  [ortholog](http://www.pantherdb.org/genes/gene.do?acc=HUMAN%7CEnsembl=ENSG00000099810%7CUniProtKB=Q13126#orthologs) | [purine nucleobase metabolic process](http://www.pantherdb.org/panther/category.do?categoryAcc=GO:0006144) |
| [HUMAN\|Ensembl=ENSG00000090621\|UniProtKB=Q13310](http://www.pantherdb.org/genes/gene.do?acc=HUMAN%7CEnsembl=ENSG00000090621%7CUniProtKB=Q13310) | Q13310 | Polyadenylate-binding protein 4  [PABPC4](http://www.pantherdb.org/genes/gene.do?acc=HUMAN%7CEnsembl=ENSG00000090621%7CUniProtKB=Q13310)  [ortholog](http://www.pantherdb.org/genes/gene.do?acc=HUMAN%7CEnsembl=ENSG00000090621%7CUniProtKB=Q13310#orthologs) | [DNA replication](http://www.pantherdb.org/panther/category.do?categoryAcc=GO:0006260)  [RNA splicing, via transesterification reactions](http://www.pantherdb.org/panther/category.do?categoryAcc=GO:0000375)  [transcription from RNA polymerase II promoter](http://www.pantherdb.org/panther/category.do?categoryAcc=GO:0006366)  [mRNA splicing, via spliceosome](http://www.pantherdb.org/panther/category.do?categoryAcc=GO:0000398)  [mRNA polyadenylation](http://www.pantherdb.org/panther/category.do?categoryAcc=GO:0006378)  [RNA splicing, via transesterification reactions](http://www.pantherdb.org/panther/category.do?categoryAcc=GO:0000375)  [protein metabolic process](http://www.pantherdb.org/panther/category.do?categoryAcc=GO:0019538)  [cell cycle](http://www.pantherdb.org/panther/category.do?categoryAcc=GO:0007049)  [ectoderm development](http://www.pantherdb.org/panther/category.do?categoryAcc=GO:0007398)  [nervous system development](http://www.pantherdb.org/panther/category.do?categoryAcc=GO:0007399) |
| [HUMAN\|Ensembl=ENSG00000084623\|UniProtKB=Q13347](http://www.pantherdb.org/genes/gene.do?acc=HUMAN%7CEnsembl=ENSG00000084623%7CUniProtKB=Q13347) | Q13347 | Eukaryotic translation initiation factor 3 subunit I  [EIF3I](http://www.pantherdb.org/genes/gene.do?acc=HUMAN%7CEnsembl=ENSG00000084623%7CUniProtKB=Q13347)  [ortholog](http://www.pantherdb.org/genes/gene.do?acc=HUMAN%7CEnsembl=ENSG00000084623%7CUniProtKB=Q13347#orthologs) | [translation](http://www.pantherdb.org/panther/category.do?categoryAcc=GO:0006412)  [regulation of translation](http://www.pantherdb.org/panther/category.do?categoryAcc=GO:0006417) |
| [HUMAN\|Ensembl=ENSG00000038274\|UniProtKB=Q9NZL9](http://www.pantherdb.org/genes/gene.do?acc=HUMAN%7CEnsembl=ENSG00000038274%7CUniProtKB=Q9NZL9) | Q9NZL9 | Methionine adenosyltransferase 2 subunit beta  [MAT2B](http://www.pantherdb.org/genes/gene.do?acc=HUMAN%7CEnsembl=ENSG00000038274%7CUniProtKB=Q9NZL9)  [ortholog](http://www.pantherdb.org/genes/gene.do?acc=HUMAN%7CEnsembl=ENSG00000038274%7CUniProtKB=Q9NZL9#orthologs) | [carbohydrate metabolic process](http://www.pantherdb.org/panther/category.do?categoryAcc=GO:0005975) |
| [HUMAN\|Ensembl=ENSG00000168003\|UniProtKB=P08195](http://www.pantherdb.org/genes/gene.do?acc=HUMAN%7CEnsembl=ENSG00000168003%7CUniProtKB=P08195) | P08195 | 4F2 cell-surface antigen heavy chain  [SLC3A2](http://www.pantherdb.org/genes/gene.do?acc=HUMAN%7CEnsembl=ENSG00000168003%7CUniProtKB=P08195)  [ortholog](http://www.pantherdb.org/genes/gene.do?acc=HUMAN%7CEnsembl=ENSG00000168003%7CUniProtKB=P08195#orthologs) | [glycogen metabolic process](http://www.pantherdb.org/panther/category.do?categoryAcc=GO:0005977) |
| [HUMAN\|Ensembl=ENSG00000132646\|UniProtKB=P12004](http://www.pantherdb.org/genes/gene.do?acc=HUMAN%7CEnsembl=ENSG00000132646%7CUniProtKB=P12004) | P12004 | Proliferating cell nuclear antigen  [PCNA](http://www.pantherdb.org/genes/gene.do?acc=HUMAN%7CEnsembl=ENSG00000132646%7CUniProtKB=P12004)  [ortholog](http://www.pantherdb.org/genes/gene.do?acc=HUMAN%7CEnsembl=ENSG00000132646%7CUniProtKB=P12004#orthologs) | [DNA replication](http://www.pantherdb.org/panther/category.do?categoryAcc=GO:0006260)  [DNA repair](http://www.pantherdb.org/panther/category.do?categoryAcc=GO:0006281)  [cell cycle](http://www.pantherdb.org/panther/category.do?categoryAcc=GO:0007049)  [regulation of catalytic activity](http://www.pantherdb.org/panther/category.do?categoryAcc=GO:0050790) |
| [HUMAN\|Ensembl=ENSG00000198959\|UniProtKB=P21980](http://www.pantherdb.org/genes/gene.do?acc=HUMAN%7CEnsembl=ENSG00000198959%7CUniProtKB=P21980) | P21980 | Protein-glutamine gamma-glutamyltransferase 2  [TGM2](http://www.pantherdb.org/genes/gene.do?acc=HUMAN%7CEnsembl=ENSG00000198959%7CUniProtKB=P21980)  [ortholog](http://www.pantherdb.org/genes/gene.do?acc=HUMAN%7CEnsembl=ENSG00000198959%7CUniProtKB=P21980#orthologs) | [cellular protein modification process](http://www.pantherdb.org/panther/category.do?categoryAcc=GO:0006464) |
| [HUMAN\|Ensembl=ENSG00000101444\|UniProtKB=P23526](http://www.pantherdb.org/genes/gene.do?acc=HUMAN%7CEnsembl=ENSG00000101444%7CUniProtKB=P23526) | P23526 | Adenosylhomocysteinase  [AHCY](http://www.pantherdb.org/genes/gene.do?acc=HUMAN%7CEnsembl=ENSG00000101444%7CUniProtKB=P23526)  [ortholog](http://www.pantherdb.org/genes/gene.do?acc=HUMAN%7CEnsembl=ENSG00000101444%7CUniProtKB=P23526#orthologs) | [purine nucleobase metabolic process](http://www.pantherdb.org/panther/category.do?categoryAcc=GO:0006144) |
| [HUMAN\|Ensembl=ENSG00000138363\|UniProtKB=P31939](http://www.pantherdb.org/genes/gene.do?acc=HUMAN%7CEnsembl=ENSG00000138363%7CUniProtKB=P31939) | P31939 | Bifunctional purine biosynthesis protein PURH  [ATIC](http://www.pantherdb.org/genes/gene.do?acc=HUMAN%7CEnsembl=ENSG00000138363%7CUniProtKB=P31939)  [ortholog](http://www.pantherdb.org/genes/gene.do?acc=HUMAN%7CEnsembl=ENSG00000138363%7CUniProtKB=P31939#orthologs) | [purine nucleobase metabolic process](http://www.pantherdb.org/panther/category.do?categoryAcc=GO:0006144) |
| [HUMAN\|Ensembl=ENSG00000167085\|UniProtKB=P35232](http://www.pantherdb.org/genes/gene.do?acc=HUMAN%7CEnsembl=ENSG00000167085%7CUniProtKB=P35232) | P35232 | Prohibitin  [PHB](http://www.pantherdb.org/genes/gene.do?acc=HUMAN%7CEnsembl=ENSG00000167085%7CUniProtKB=P35232)  [ortholog](http://www.pantherdb.org/genes/gene.do?acc=HUMAN%7CEnsembl=ENSG00000167085%7CUniProtKB=P35232#orthologs) | [DNA replication](http://www.pantherdb.org/panther/category.do?categoryAcc=GO:0006260)  [cell cycle](http://www.pantherdb.org/panther/category.do?categoryAcc=GO:0007049) |
| [HUMAN\|Ensembl=ENSG00000105379\|UniProtKB=P38117](http://www.pantherdb.org/genes/gene.do?acc=HUMAN%7CEnsembl=ENSG00000105379%7CUniProtKB=P38117) | P38117 | Electron transfer flavoprotein subunit beta  [ETFB](http://www.pantherdb.org/genes/gene.do?acc=HUMAN%7CEnsembl=ENSG00000105379%7CUniProtKB=P38117)  [ortholog](http://www.pantherdb.org/genes/gene.do?acc=HUMAN%7CEnsembl=ENSG00000105379%7CUniProtKB=P38117#orthologs) | [respiratory electron transport chain](http://www.pantherdb.org/panther/category.do?categoryAcc=GO:0022904) |
| [HUMAN\|Ensembl=ENSG00000100983\|UniProtKB=P48637](http://www.pantherdb.org/genes/gene.do?acc=HUMAN%7CEnsembl=ENSG00000100983%7CUniProtKB=P48637) | P48637 | Glutathione synthetase  [GSS](http://www.pantherdb.org/genes/gene.do?acc=HUMAN%7CEnsembl=ENSG00000100983%7CUniProtKB=P48637)  [ortholog](http://www.pantherdb.org/genes/gene.do?acc=HUMAN%7CEnsembl=ENSG00000100983%7CUniProtKB=P48637#orthologs) | [sulfur compound metabolic process](http://www.pantherdb.org/panther/category.do?categoryAcc=GO:0006790) |
| [HUMAN\|Ensembl=ENSG00000004455\|UniProtKB=P54819](http://www.pantherdb.org/genes/gene.do?acc=HUMAN%7CEnsembl=ENSG00000004455%7CUniProtKB=P54819) | P54819 | Adenylate kinase 2, mitochondrial  [AK2](http://www.pantherdb.org/genes/gene.do?acc=HUMAN%7CEnsembl=ENSG00000004455%7CUniProtKB=P54819)  [ortholog](http://www.pantherdb.org/genes/gene.do?acc=HUMAN%7CEnsembl=ENSG00000004455%7CUniProtKB=P54819#orthologs) | [purine nucleobase metabolic process](http://www.pantherdb.org/panther/category.do?categoryAcc=GO:0006144)  [pyrimidine nucleobase metabolic process](http://www.pantherdb.org/panther/category.do?categoryAcc=GO:0006206) |
| [HUMAN\|Ensembl=ENSG00000004478\|UniProtKB=Q02790](http://www.pantherdb.org/genes/gene.do?acc=HUMAN%7CEnsembl=ENSG00000004478%7CUniProtKB=Q02790) | Q02790 | Peptidyl-prolyl cis-trans isomerase FKBP4  [FKBP4](http://www.pantherdb.org/genes/gene.do?acc=HUMAN%7CEnsembl=ENSG00000004478%7CUniProtKB=Q02790)  [ortholog](http://www.pantherdb.org/genes/gene.do?acc=HUMAN%7CEnsembl=ENSG00000004478%7CUniProtKB=Q02790#orthologs) | [cellular protein modification process](http://www.pantherdb.org/panther/category.do?categoryAcc=GO:0006464)  [cellular process](http://www.pantherdb.org/panther/category.do?categoryAcc=GO:0009987) |

**Supplementary Table S5⏐qPCR primer sequences used in the described studies.**

| **Transcript Target** | **Forward primer (5’-3’)** | **Reverse primer (5’-3’)** |
| --- | --- | --- |
| AXIN2 | CTCCCCACCTTGAATGAAGA | TGGCTGGTGCAAAGACATAG |
| MFN1 | CTCTCCGCCTTTACTTCTC | TCTGCCATTATGCTAAGTCTCC |
| MFN2 | CTCAAGACTATAAGCTGCGA | TCAAGCCATCTATCATGTCC |
| OPA1 | TATGACAGAACCGAAAGGGA | GAATAACCCTCAAGCTGTCCT |
| DRP1 | GAAATGCTACTGGTCCTCGT | ACGAAGAAGACAAGTCACCA |
| UBC | CTGCTCATAAGACTCGGCCT | AAGATCTGCATTGTCAAGTGACG |
| β-actin | AGAGCAAGAGAGGCATCCTC | CTCAAACATGATCTGGGTCA |
| YWHAZ | ACTTTTGGTACATTGTGGCTTCAA | CCGCCAGGACAAACCAGTAT |

**Supplementary Table S6⏐siRNA oligos used in the described studies.**

| β-catenin | CUAUCUGUCUGCUCUAGUA[dT][dT] | (Ambion, validated) |
| --- | --- | --- |
| PTEN | CGAACUGGUGUAAUGAUAU[dT][dT] | (Ambion, validated) |
| Control | Control siRNA #1 (sequence not disclosed) | (Ambion, validated) |

**References**

1 Jenei, V. *et al.* A t-butyloxycarbonyl-modified Wnt5a-derived hexapeptide functions as a potent antagonist of Wnt5a-dependent melanoma cell invasion. *Proc Natl Acad Sci U S A* **106**, 19473-19478, doi:10.1073/pnas.0909409106 (2009).

2 Gogvadze, V., Orrenius, S. & Zhivotovsky, B. Multiple pathways of cytochrome c release from mitochondria in apoptosis. *Biochim Biophys Acta* **1757**, 639-647, doi:10.1016/j.bbabio.2006.03.016 (2006).

3 Cancer Genome Atlas, N. Genomic Classification of Cutaneous Melanoma. *Cell* **161**, 1681-1696, doi:10.1016/j.cell.2015.05.044 (2015).

4 Kielhorn, E. *et al.* Tissue microarray-based analysis shows phospho-beta-catenin expression in malignant melanoma is associated with poor outcome. *Int J Cancer* **103**, 652-656, doi:10.1002/ijc.10893 (2003).
