# Supplementary figures and images for: WNT/β-catenin signaling regulates mitochondrial activity to alter the oncogenic potential of melanoma in a PTEN-dependent manner
Source: Oncogene. 2017 Jan 16;36(22):3119–36. doi: 10.1038/onc.2016.450 (PMC5467017; doi:10.1038/onc.2016.450)

**a**

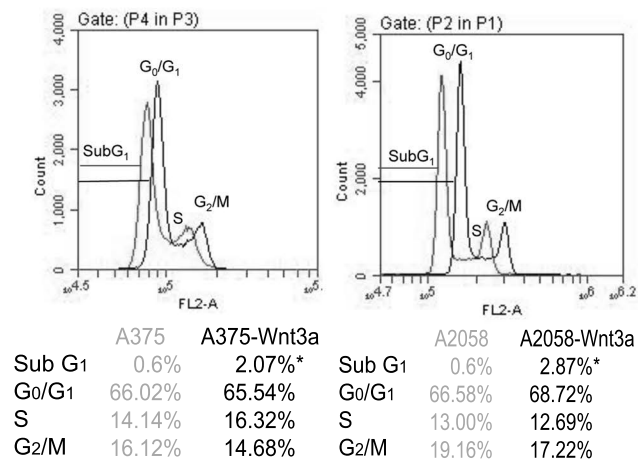

**b**

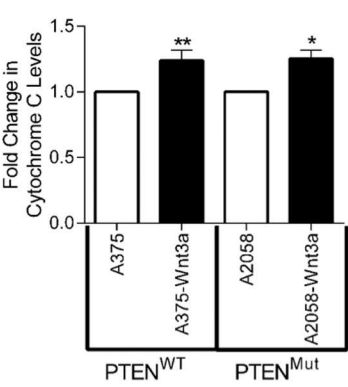

Supplement: Supplementary Figure 1 [file onc2016450x2.pdf]

**Oncogene Brown et. al. 2016 Figure 2 supplementary**

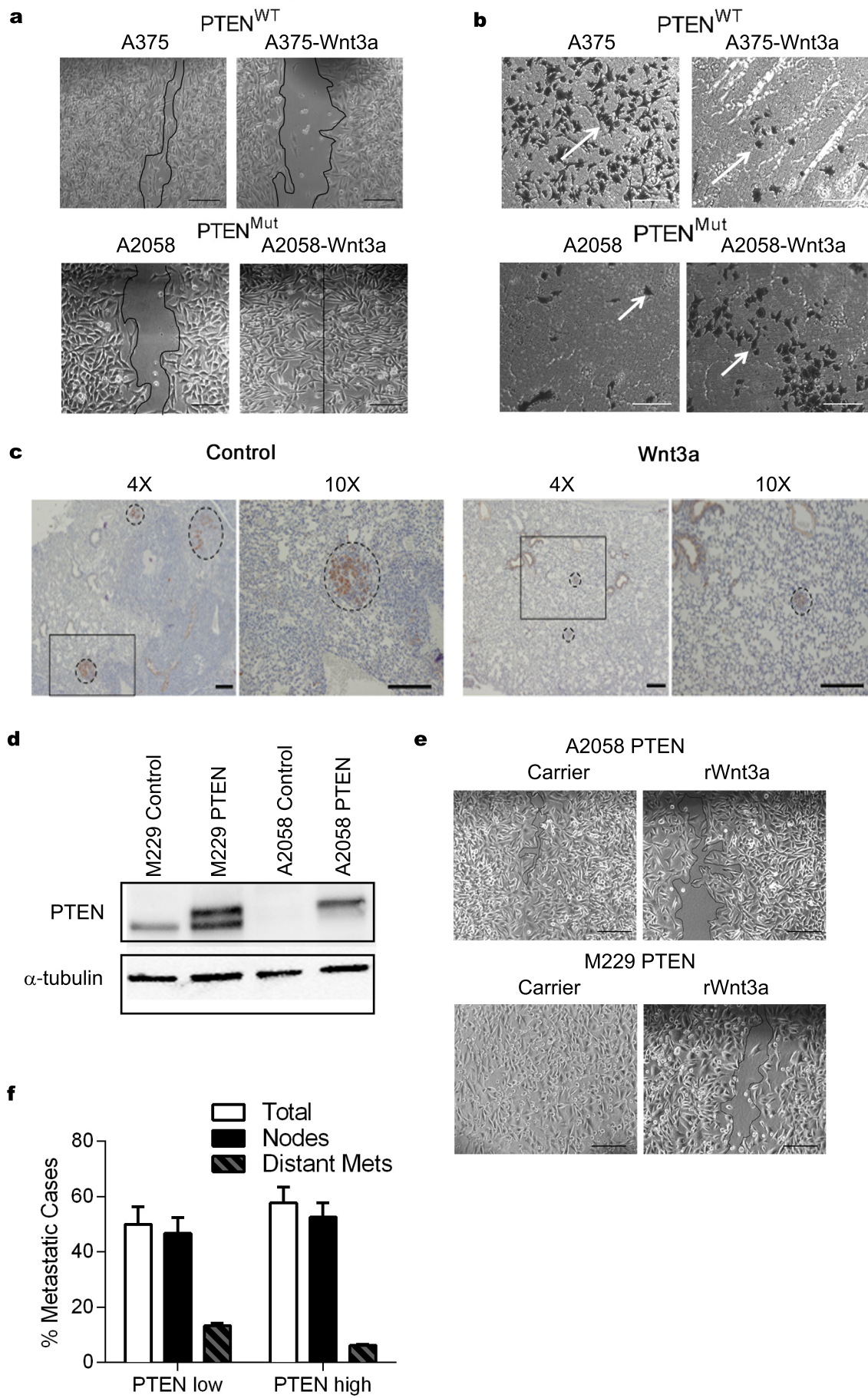

Supplement: Supplementary Figure 2 [file onc2016450x3.pdf]

**a**

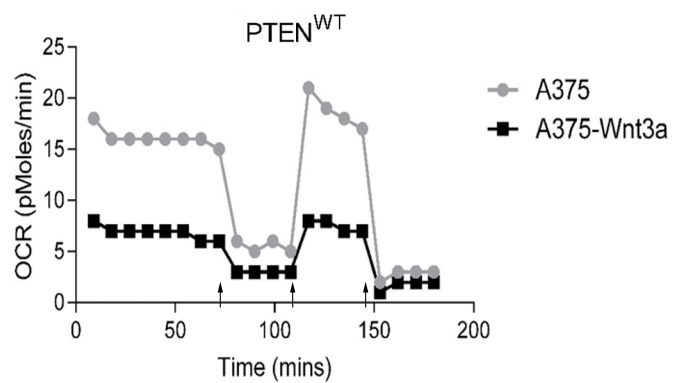

**b**

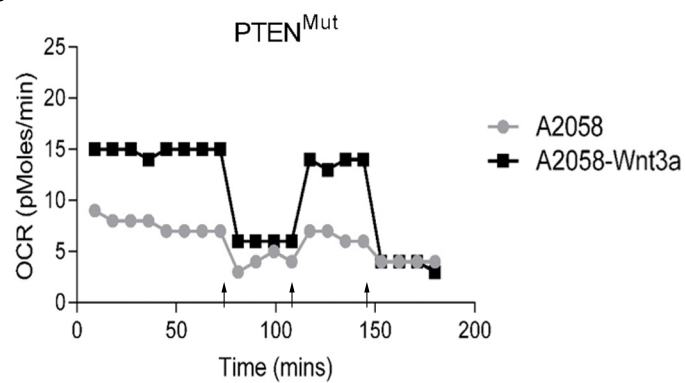

**c**

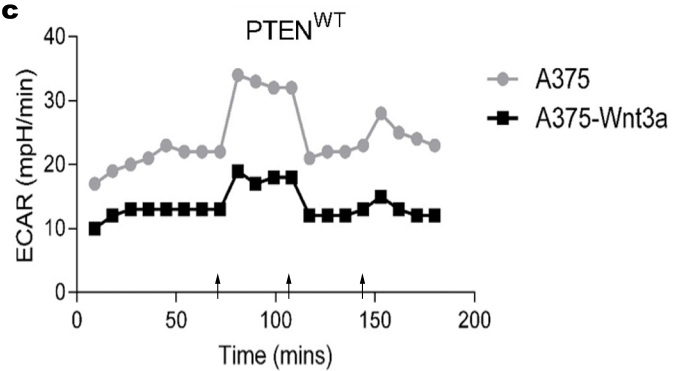

**d**

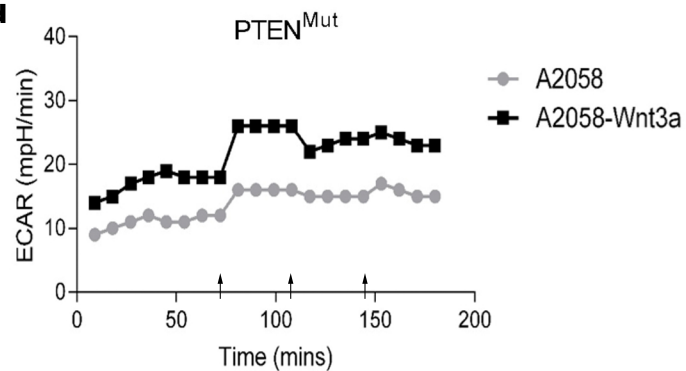

Supplement: Supplementary Figure 3 [file onc2016450x4.pdf]

**a**

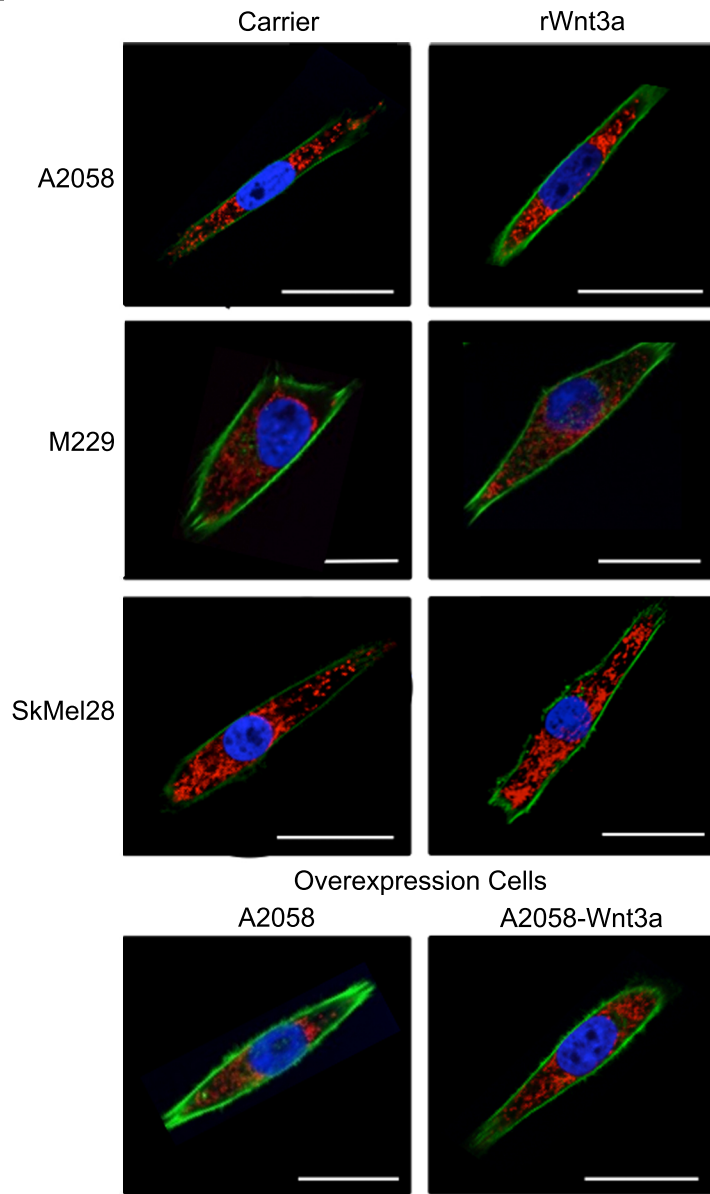

**b**

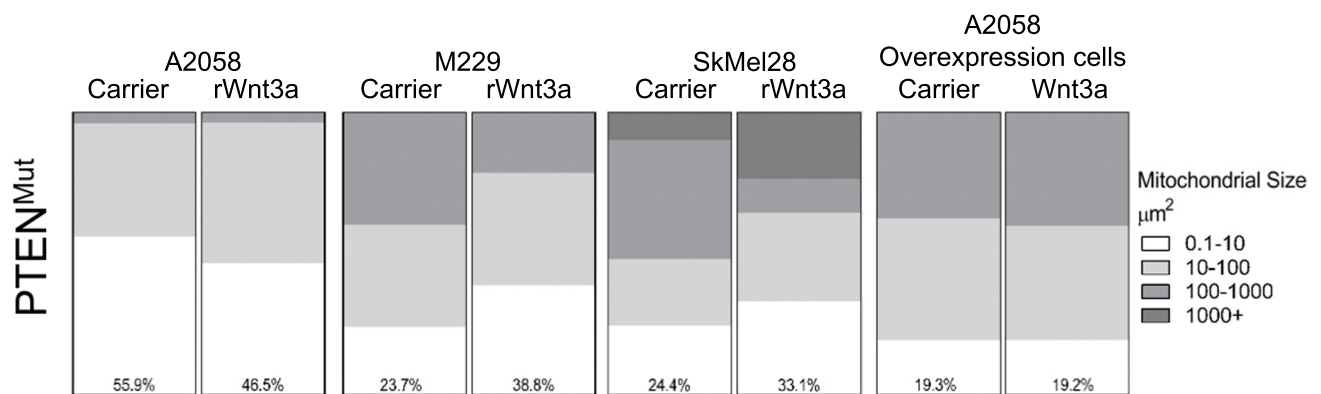

Supplement: Supplementary Figure 4 [file onc2016450x5.pdf]

**a**

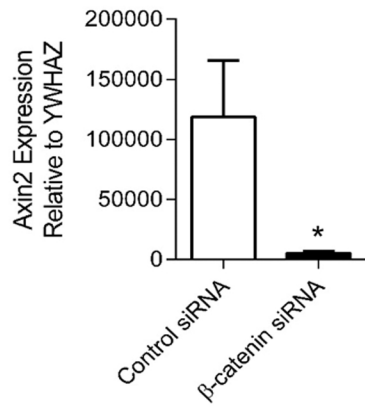

**b**

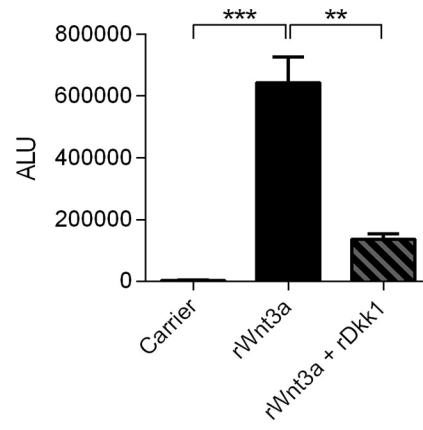

**c**

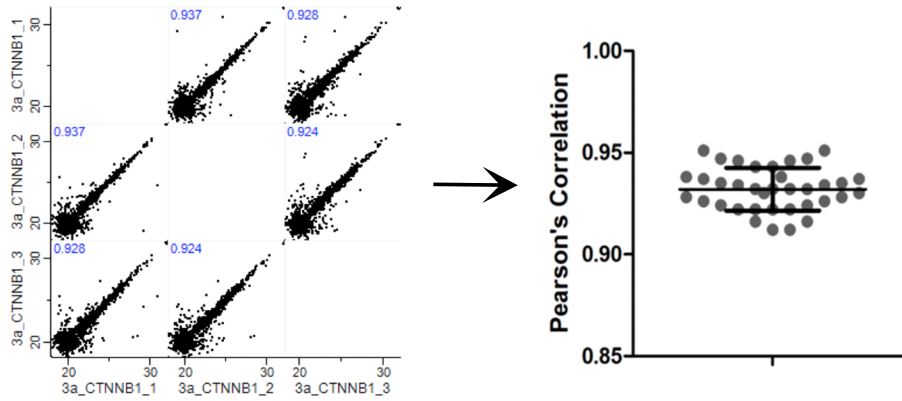

Supplement: Supplementary Figure 5 [file onc2016450x6.pdf]

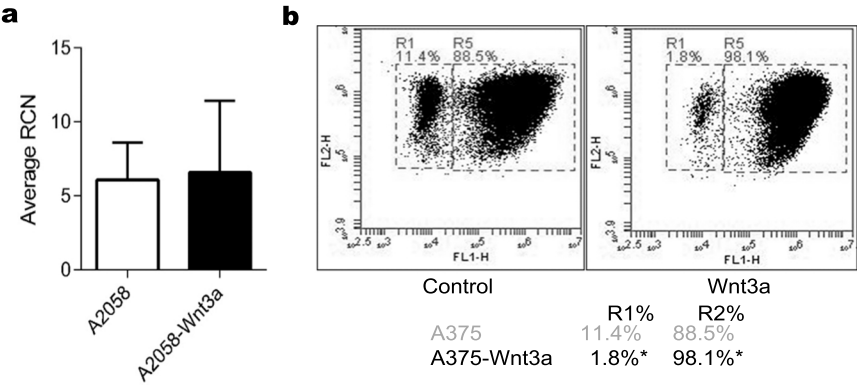

Supplement: Supplementary Figure 6 [file onc2016450x7.pdf]

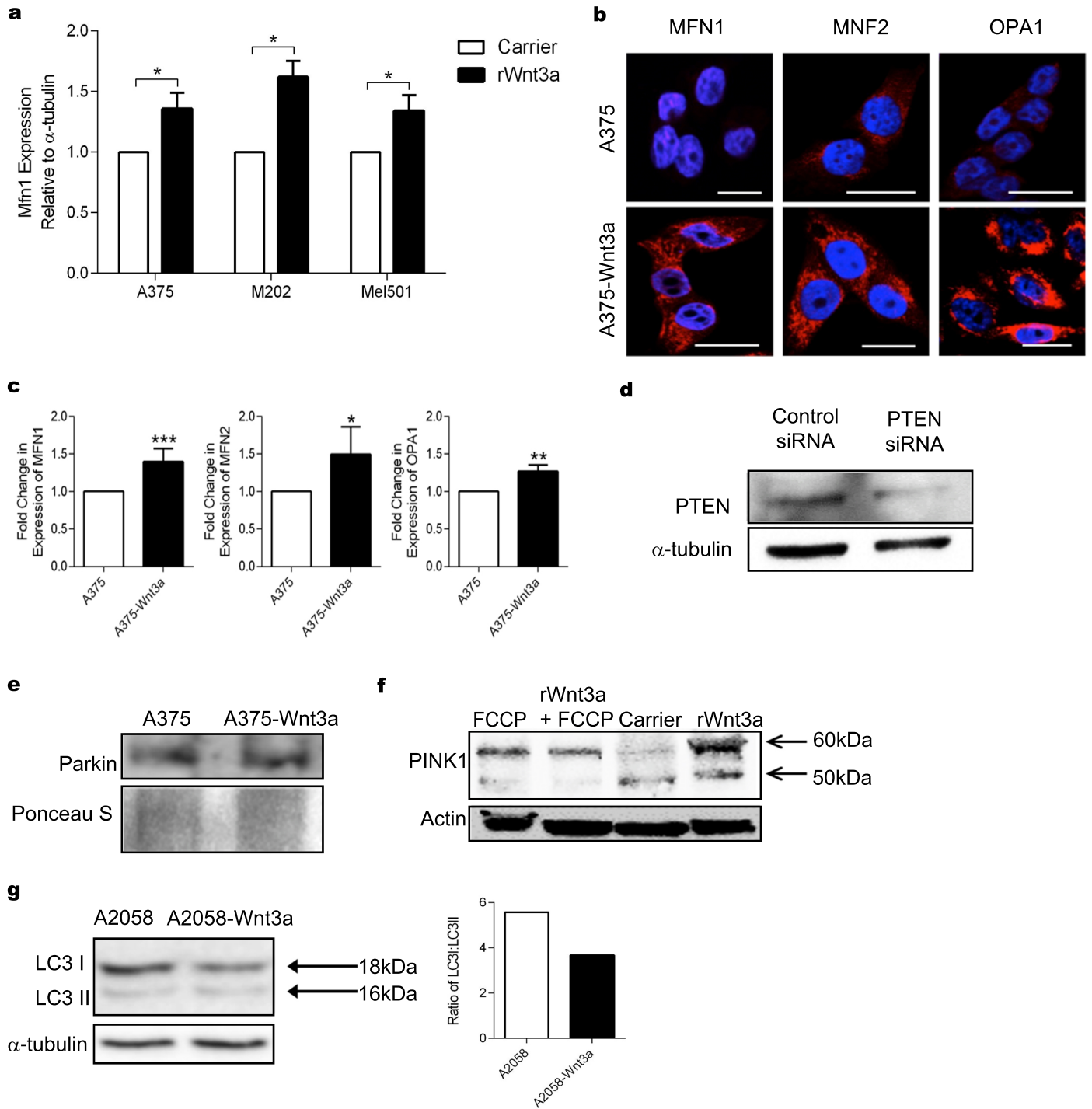

Supplement: Supplementary Figure 7 [file onc2016450x8.pdf]

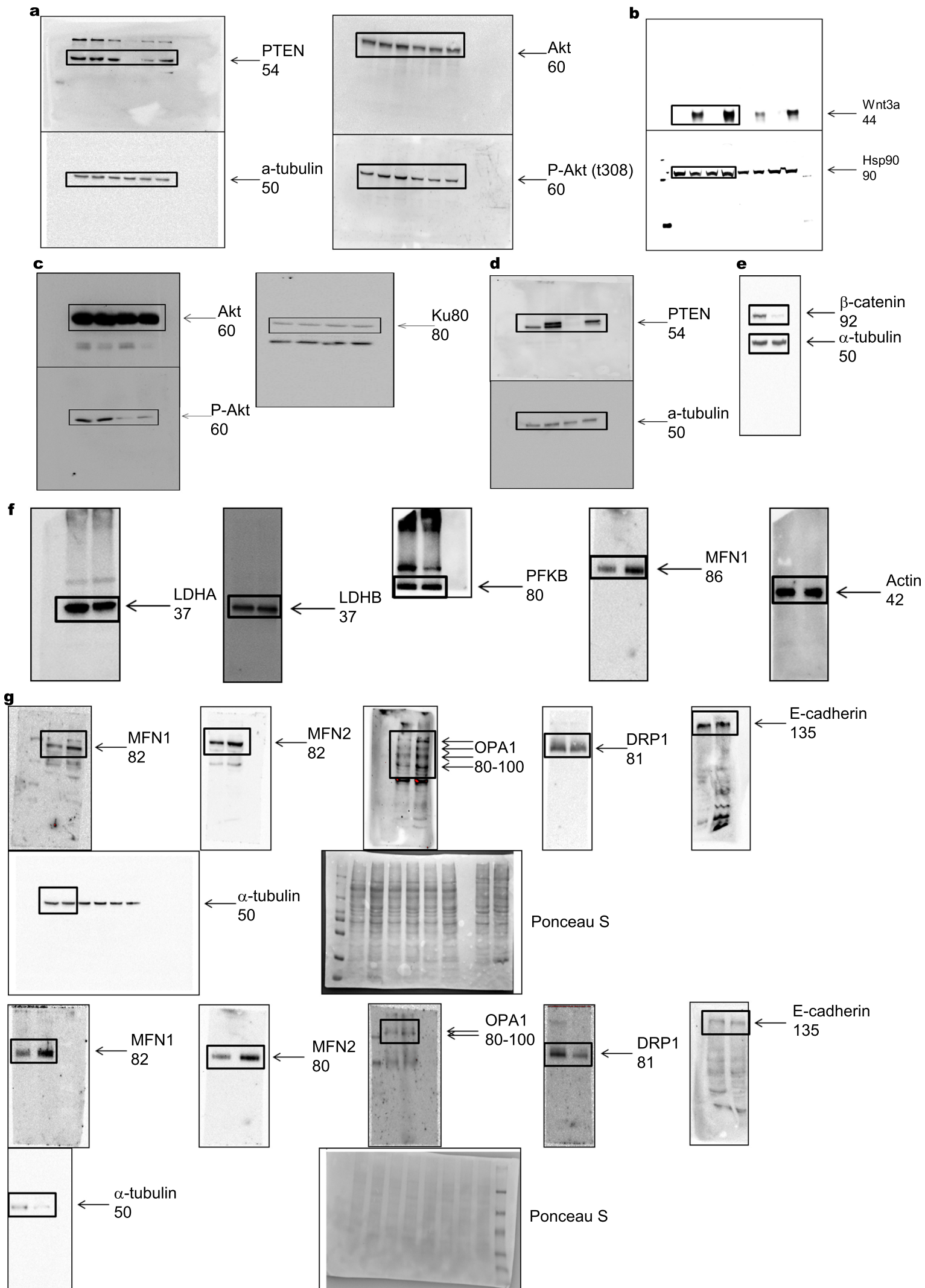

Supplement: Supplementary Figure 8i [file onc2016450x9.pdf]

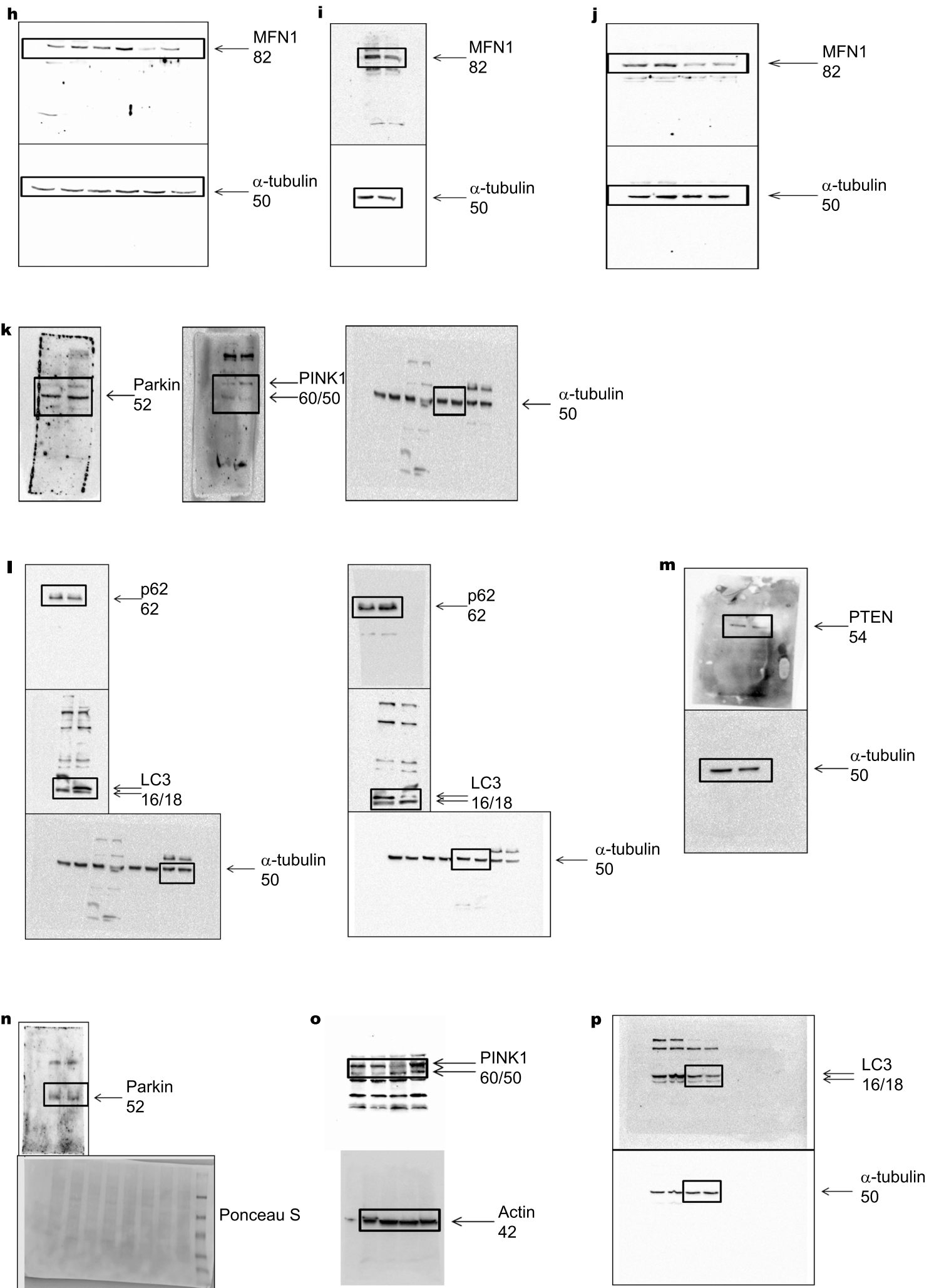

Supplement: Supplementary Figure 8ii [file onc2016450x10.pdf]
